# Beyond Partitioning: Using Force Field Science to Evaluate Electrostatics Models

**DOI:** 10.1021/acs.jctc.6c00039

**Published:** 2026-02-21

**Authors:** A. Najla Hosseini, Kristian Kříž, David van der Spoel

**Affiliations:** Department of Cell and Molecular Biology, 27106 Uppsala University, Husargatan 3, Box 596, SE-75124 Uppsala, Sweden

## Abstract

Accurate models for electrostatic and induction interactions are fundamental for computational molecular science, including drug discovery, studies of biomolecular systems and materials design. Given a precise model of the entire charge distributions, the electrostatic interaction between molecules can be calculated accurately using Coulomb’s law. Here, we evaluate partitioning methods for deriving charges from electron density as well as the popular method of fitting point charges for use in force field calculations to the electrostatic potential (ESP). For the data set used in this work, which consists of charged amino-acid side chain analogs, inorganic ions and water, the best of these methods yield a root-mean-square deviation (RMSD) of 17 kJ/mol. By combining positive point charges (PC) with Gaussian or Slater distributed negative charges, ESP-fitted models predict electrostatic interactions approximately 30% better than just point charges (RMSD 12 kJ/mol), similar to the Minimal Basis Iterative Stockholder (MBIS-S) method [ VerstraelenT., . J. Chem. Theory Comput. 2016, 12, 3894–3912.]27385073 10.1021/acs.jctc.6b00456that employs a PC and a Slater charge as well. Since interaction energies are perhaps the most important deliverable of force field calculations, it may be advantageous to train models directly to reproduce energy components from symmetry-adapted perturbation theory (SAPT) calculations, rather than taking a detour through monomer-based charge models. To this end, we employ machine learning using the Alexandria Chemistry Toolkit [ van der SpoelD., . Digit. Discovery 2025, 4, 1925–1935.] to generate parameters for multiple physics-based models that reproduce electrostatic and, optionally, induction interaction energies from SAPT calculations of compound dimers. For a nonpolarizable model combining a PC and a Gaussian distributed charge on the core, the RMSD drops to 3 kJ/mol thanks to direct training on dimer energy components. The approach outlined in this work consists of applying force field science to make apples-to-apples comparisons between models and machine learning to design physics-based force fields that yield interaction energies consistent with SAPT calculations. Together, these tools will enable rapid progress in force field development and enhance the predictive power of molecular simulations for applications in many fields of science.

## Graphical abstract

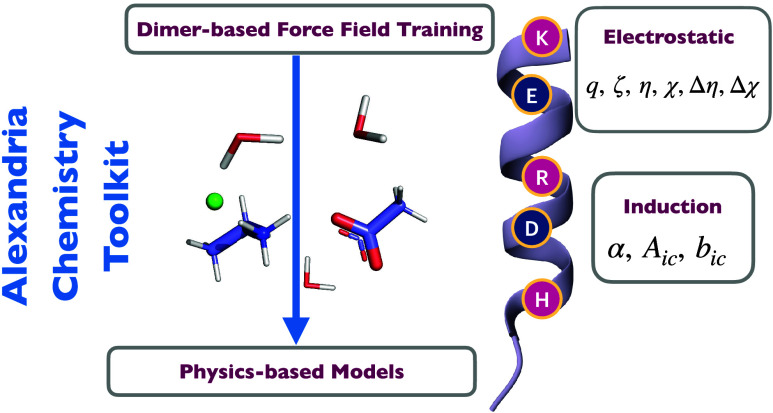

## Introduction

Interactions between monatomic ions or water and biological molecules are of paramount importance in virtually all biological processes, such as the regulation of cellular physiological functions,^1^ mechanosensing,^2^ liquid–liquid phase separation,^3^ the function of sensory receptors^4^ or, for instance, the formation of protein-nucleic acid complexes.^5^ Furthermore, cellular functions may be influenced by changes in solvent environments, including varying ionic activities between cellular and subcellular compartments.^6^ To illustrate this, Figure 1 shows interactions of Li^+^ with a glutamate receptor that mediates compound excitatory synaptic transmission in the brain;^7^ Na^+^ binding to a ruthenium anticancer agent and lysozyme;^8^ interactions with, or exporting of fluoride through the Fluc ion channel found in microorganisms to contend with hazards posed by environmental fluoride;^9^ the interaction of bromide with a potent neuron-inhibiting optogenetics tool;^10^ the voltage-dependent CLC-1 chloride channel;^11^ and the T-cell potassium channel Kv1.3 with immunoglobulin modulators.^12^ Understanding the interactions between the aqueous solvent or ions and biological macromolecules is therefore of both fundamental and applied biomedical interest. In this study, we investigate how best to derive electrostatic and induction models for studying intermolecular interactions. To this end, we use a data set of charged amino-acid side chain analogs (Table 1), water and inorganic ions, and apply machine learning using the Alexandria Chemistry Toolkit^13^ to train model parameters.

**Figure 1:**
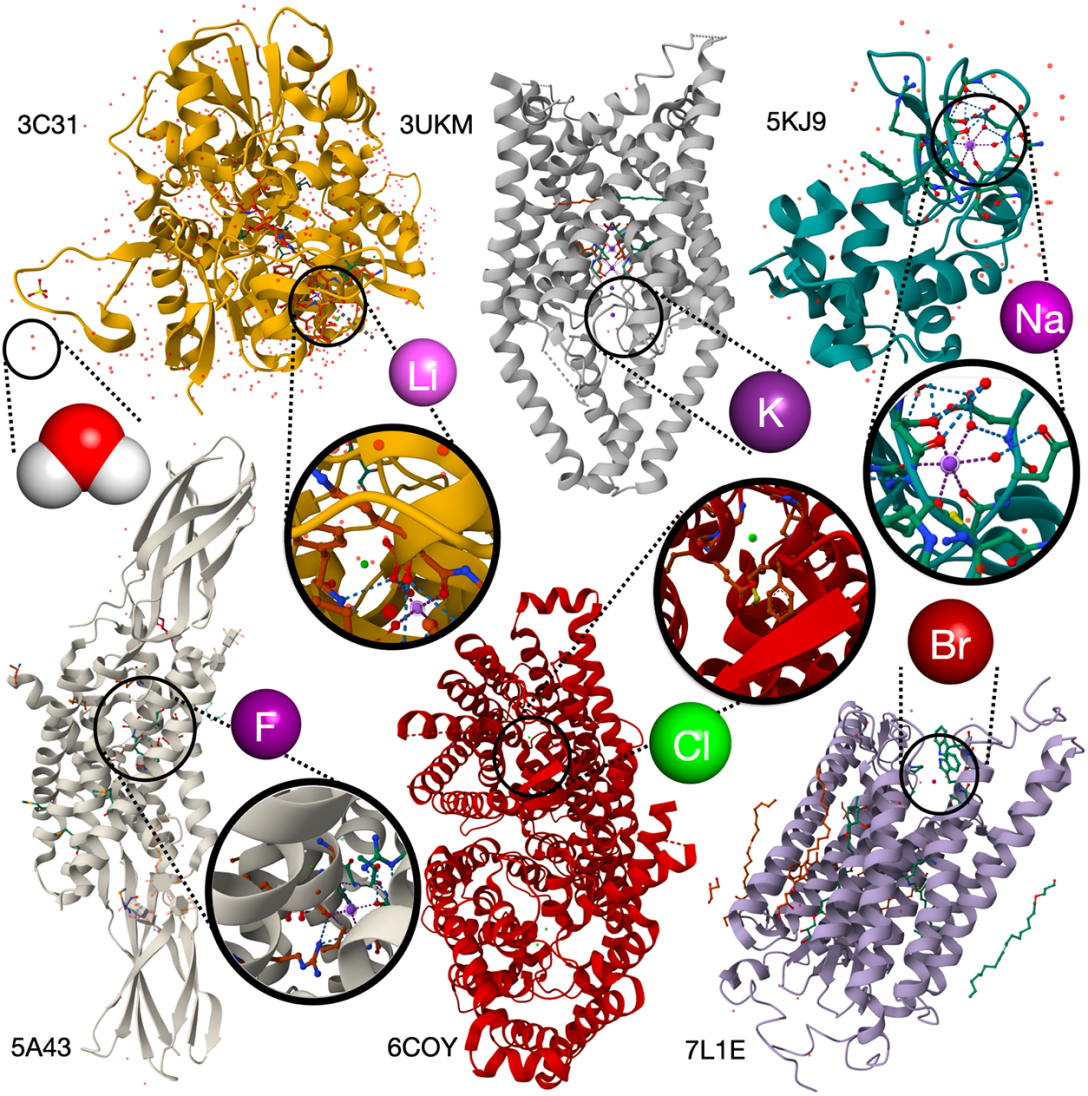
Interactions between proteins, ions (Li^+^, Na^+^, K^+^, F^–^, Cl^–^, Br^–^), and water, including Li^+^ with glutamate receptors in synaptic transmission,^7^ Na^+^ binding to ruthenium anticancer agents and lysozyme,^8^ fluoride export via Fluc channels,^9^ bromide’s role in neuron inhibition,^10^ Cl^–^ in CLC-1 channels,^11^ and K^+^ in the Kv1.3 T cell channel.^12^

**Table 1:** Side Chain Analogs for Amino Acids AA and Their Charge *q* Used in This Work

compound	AA	*q*
formate	D, E	–1
acetate	D, E	–1
propanoate	D, E	–1
butanoate	E	–1
ammonium	K	1
methylammonium	K	1
ethylammonium	K	1
imidazolium	H	1
guanidinium	R	1

Computational chemistry studies can offer profound insights into the intricate interactions between water molecules or ions and proteins or nucleic acids.^5,14,15^ For quantitative modeling of large systems and in particular studies of the dynamics of biomolecules, sufficiently simple models are needed and therefore electrostatic interactions in biological systems are most often modeled using partial (point) charges. Historically, a multitude of computational methods have been explored to determine partial charges. The first such approaches involved partitioning the electron density from quantum mechanical (QM) calculations into atomic populations without any postprocessing.^16,17^ However, the charges produced by these and related methods^18^ rely heavily on the partitioning method as well as the QM level of theory used.^19^ Later partitioning methods took information about covalent bonds into account, which led to these charges being more “reasonable”.^20,21^ A number of other partitioning methods have been presented in the past decade or so,^22,23^ but we will not comment on those here.

A particularly widely used method is based on fitting partial charges to reproduce the quantum chemical electrostatic potential Φ (ESP) generated by a compound.^24^ Typically, a few layers of grid points around the compound are used at distances from the nuclei corresponding to 1.4 to 2 times the van der Waals radius of the atoms,^25^ the rationale being that these boundaries correspond to distances for direct noncovalent interactions. Very recently, electron diffraction has been used to estimate partial charges experimentally.^26^ This offers the intriguing possibility of validating computational predictions of partial charges, in particular if a large amount of data would be collected. However, the authors noted that methods deriving partial charges from the electrostatic potential exhibit a moderate correlation with the experimental data (Pearson correlation coefficients around 0.5 for the compounds studied).

In what follows, we will first introduce the lack of information problem that hampers the use of ESP fitting, in particular for models that are more accurate than point charges. Then, we demonstrate that it is possible to reproduce electrostatic and induction energies from symmetry-adapted perturbation theory (SAPT)^27^ by training force field models using the Alexandria Chemistry Toolkit (ACT),^13^ a machine learning software designed to generate force field models from scratch. In brief, a number of physical models for charge distributions in molecules are trained to reproduce the corresponding electrostatic and induction energies from SAPT using the ACT. The accuracies of these models are compared to ESP fitting and other well-known algorithms to derive electrostatic parameters from quantum chemistry. The models we derive in this work are of an “intermediate” complexity, that is, they will be more accurate than just point charges, but less complex than the multipole-based models such as Amoeba,^28,29^ MASTIFF^30,31^ or CLIFF.^32^ To model charge and/or exchange anisotropy, we instead rely on virtual-sites on atoms,^33^ on the bisector of the water molecule^34^ or on lone-pairs or σ-holes.^35^

## Theory

The electrostatic potential Φ_QM_(**r**) at position **r** due to a charge distribution ρ_QM_(**r**_*j*_) centered at the position **r**_*j*_ of particle *j* from quantum mechanics is given by

1 $$\Phi_{\mathrm{QM}}\left(r\right)=\frac{1}{4\pi \varepsilon_{0}}\int_{-\infty }^{\infty }\frac{\rho_{\mathrm{QM}}\left(r_{j}\right)}{∥r_{j}-r∥}d^{3}r_{j}$$

where ε_0_ is the permittivity of vacuum. Molecular models for electrostatics can be derived by fitting the charges *q*_*j*_ such that

2 $$\Phi_{\mathrm{MM}}\left(r\right)=\frac{1}{4\pi \varepsilon_{0}}\sum_{j=1}^{N}\frac{q_{j}}{∥r_{j}-r∥}$$

approximates Φ_QM_(**r**). For this purpose, point charges (PC, eq 2),^24^ Gaussian-distributed charges (GC)^36^ or electric multipoles^28,31^ have been used. Recent variants of the ESP fitting method use improved atomic radii for the quantum chemical ESP calculation,^37^ apply averaging of charges generated using different dielectric media^38^ or add virtual sites to better reproduce the ESP.^39^ In yet another study, different sets of ESP partial charges were used for the same compounds in water and phospholipid membranes, to improve the description of membrane transfer.^40^ These examples show that there still are efforts to apply and to improve the ESP fitting method for determining partial charges. However, it has been known for a long time that ESP fitting can cause artifacts, for instance, due to buried atoms in larger compounds becoming an error sink for the fit,^19^ leading to carbon atoms with charges of more than 2 e (electron) or less than −2e. To alleviate these issues somewhat, different variants such as restrained ESP (RESP) were introduced.^25^

Although the community is aware that there are issues with transferability of ESP charges,^38,40^ it seems that the fact that the information from Φ_QM_(**r**) used in ESP fitting is incomplete, may have been overlooked. The Poisson equation gives the relation between the electrostatic potential, Φ­(**r**) and the charge distribution ρ­(**r**) that generates it

3 $$\nabla^{2}\Phi \left(r\right)=-\frac{\rho \left(r\right)}{\varepsilon_{0}}$$

where ∇ is the gradient operator. Equation 3 informs us that the relation between Φ­(**r**) and ρ­(**r**) is local in space. If *V*_Φ_ is the volume sampled by the ESP grid points, the charge density ρ­(**r** ∈*V*_Φ_) can be determined from Φ­(**r**∈*V*_Φ_) but ρ­(**r**∉*V*_Φ_) becomes an extrapolation, the quality of which outside of the fitting region depends on the mathematical model used for ρ. This is particularly cumbersome when considering that the electrostatic interaction *E*_el_ between two charge distributions ρ_*i*_(**r**_*i*_) and ρ_*j*_(**r**_*j*_) is given by

4 $$E_{\mathrm{el}}\left(r_{\mathrm{ij}}\right)=\frac{1}{4\pi \varepsilon_{0}}\int_{-\infty }^{\infty }\frac{\rho_{i}\left(r_{i}\right)\rho_{j}\left(r_{j}\right)}{∥r_{i}-r_{j}∥}d^{3}r_{i}d^{3}r_{j}$$

where it is important to note that the integration is over **R**3, i.e., the entire space. Fitting a charge model to an ESP grid in a limited volume *V*_Φ_ will yield a charge distribution ρ­(**r**∉*V*_Φ_) of unknown accuracy, which in turn will propagate to the calculations of electrostatic interaction energies in an unpredictable fashion (eq 4). It is well-established that ESP charges depend strongly on the specific volume *V*_Φ_ used for fitting^19^ and as we argue above, there is no guarantee that charge distributions derived from ESP fits on grid points in a limited volume *V*_Φ_ will reproduce electrostatic interactions faithfully, since these depend on an accurate charge distribution everywhere (eq 4). It is obvious that a lack of data in any method, including the dimer-based training of electrostatics proposed in this work, may lead to poor models. However, in contrast to ESP fitting,^19^ the force field science tools in the ACT^13^ make it straightforward to incrementally improve the accuracy of models by selectively adding more data points and by introducing physics that the model lacks.^41^

## Methods

### Electrostatic Potential for Inorganic Ions

To investigate whether it is feasible to design accurate charge distribution models that reproduce the ESP from quantum chemistry, we used monatomic ions that can be treated analytically. Since atoms consist of a positively charged nucleus surrounded by an electron cloud, it seems reasonable^33,42^ that models of the electrostatic potential can be improved by combining a point charge core, representing the nucleus, with one or two either Gaussian (P+G, P+G+G) or Slater distributed charges (P+1S, P+1S+2S), represent the electron cloud. Analytical expressions for the Slater functions were taken from Hentschke^43^ as described previously.^36^

The electrostatic potentials of Li^+^, Na^+^, K^+^, F^–^, Cl^–^, and Br^–^ were computed at distances from 0 to 4.5 Å in increments of 0.1 Å at the Hartree–Fock (HF) level of theory with the aug-cc-pVTZ basis set^44^ using the Psi4 software,^45^ version 1.9.1. The ESP calculation included points at a close distance from the nucleus in an attempt to include more data than in the standard way (ref 25 and see below). The HF level of theory was chosen since it corresponds to the one used in SAPT0 for electrostatics.^46,47^ Analytical fits of the four models mentioned above were implemented using the Scientific Python library.^48^

### Database Generation

The database for this work includes 94 dimers of charged amino acid side chain analogs (Table 1), water, and inorganic ions (Table S1). First, we minimized dimer structures using GAFF,^49^ and then scans were performed along the vector defining the shortest atomic distance. In addition, randomly oriented dimers were generated at distances close to the sum of van der Waals radii of the closest atoms. Symmetry-adapted perturbation theory calculations^27^ were performed using the SAPT2+(CCD)­δMP2 method with an augmented triple-ζ basis set^44^ using Psi4 version 1.9.1 ^45^ as recommended by Parker et al.^46^ to calculate electrostatic and induction energies for all of the dimers (Table S1). Of all the structures generated, 14371 dimer structures were selected based on their distances (shortest distance between atoms should be less than 6 Å) and energies (exchange energy less than 0.04 hartree). Of these, 9379 structures were used for training, and 4992 points as a test set (Table S1, data are available on GitHub^50^).

In addition, electrostatic potentials were computed for the side chain analogs (Table 1), water and inorganic ions in the “traditional” manner. That is, four layers of grid points were generated at distances corresponding to 1.4, 1.6, 1.8, and 2.0 times the van der Waals radius of the nearest atom^25^ and the electrostatic potential was computed at the MP2/aug-cc-pVTZ level of theory^44,51^ using Psi4.^45^ This level of theory was chosen since it performed well in a comparison of methods for fitting charges to the electrostatic potential.^38^ These calculations were used to investigate the effect of including screened charges to the model, by performing training of charges and distributions widths for models consisting of a point charge and either a Gaussian or Slater-distributed charges to the electrostatic potential.

To compare machine-learned models against existing schemes, the Gaussian software^52^ was used to generate partial charges for the Mulliken,^17^ Hirshfeld,^20^ ESP,^24^ and CM5 ^21^ models, at the MP2 level of theory^51^ with the aug-cc-pVTZ basis set.^44^ For K^+^, the def2-TZVPP basis set^53^ was used. BCC^54^ charges were generated using Antechamber,^55,56^ and RESP charges^25^ were generated using Antechamber based on the calculation of the electrostatic potential using the Gaussian software.^52^ Finally, ESP charges for about 5100 compounds were taken from the Alexandria Library,^57^ one of the few QM data sets for which ESP grids are available.^58^

A development version of the Psi4 package^45^ was used to calculate effective charges, core charges and 1S Slater distribution widths for the Minimal Basis Iterative Stockholder (MBIS) method.^59^ Monomer structures (Table 1) were optimized at the MP2 level of theory with the aug-cc-pVTZ basis set before performing the MBIS calculations. For those, the CCSD method^60^ was used because MBIS analysis of in particular anions is sensitive to the method used.^61^ Two variants of MBIS were evaluated, MBIS with effective charges on atoms, and MBIS-S which combines a positive point charge core with a 1S Slater-distributed negative virtual site, identical to the PC+SV3* model (Table 2) derived in this work. For the MBIS-S method, charges and valence widths σ were averaged over chemically identical atoms (i.e., hydrogen in a methyl group) as is common for force field calculations. Calculations using Slater functions in the ACT are based on the wave function^36^

5 $$\Psi \left(r,n\right)=\sqrt{\frac{{\left(2\zeta \right)}^{\left(2n+1\right)}}{4\pi \left(2n\right)!}}r^{n-1}e^{-\zeta r}$$

which needs to be squared to be able to compare it to the electron density equation used for MBIS.^59^ By also substituting *n* = 1, the electron density is obtained

6 $$\rho \left(r\right)=\frac{\zeta^{3}}{\pi }e^{-2\zeta r}$$

In other words, to use MBIS valence widths σ (see ref 59) in the ACT, they have to be multiplied by two, that is

7 $$\zeta =\frac{1}{2\sigma }$$

**Table 2:** Models Generated by ACT in This Work^a^

model	charges	VS	polar.	water	target	parameters
PC+GV3x	P+G	*x*	-	3P	ESP	*q*_V_, ζ_V_
PC+SV3x	P+S	*x*	-	3P	ESP	*q*_V_, ζ_V_
PC+GV4x	P+G	*x*	-	4P	ESP	*q*_V_, ζ_V_, *r*_OM_
PC+SV4x	P+S	*x*	-	4P	ESP	*q*_V_, ζ_V_, *r*_OM_
PC	P	-	-	3P	SAPT	χ, η, Δχ, Δη
GC	G	-	-	3P	SAPT	χ, η, Δχ, Δη, ζ
SC	S	-	-	3P	SAPT	χ, η, Δχ, Δη, ζ
PC+GV4	P+G	*x*	-	4P	SAPT	χ, η, Δχ, Δη, *q*_V_, ζ_V_, *r*_OM_
PC+SV4	P+S	*x*	-	4P	SAPT	χ, η, Δχ, Δη, *q*_V_, ζ_V_, *r*_OM_
PC+GS4	P+G	-	*x*	4P	SAPT	χ, η, Δχ, Δη, *q*_*S*_, ζ_*S*_, *r*_OM_ α, A_ic_, b_IC_

a Charges either Point (P), Gaussian (G), Slater (S) or a combination. Virtual sites (VS) were added at the atomic position to all atoms where indicated. Water models were either 3P (TIP3P-like) or 4P (TIP4P-like). Training either targeted the electrostatic potential (ESP) of monomeric compounds or symmetry-adapted perturbation theory (SAPT) interaction energy components of dimers. The last column shows what parameters were trained: *q*_x_ and ζ_x_ are charge and distribution width on virtual sites (*x* = V) or shells (*x* = S), rOM is the distance between the water oxygen and the virtual site on the bisector in TIP4P-like models, χ, η, Δχ and Δη are parameters of the split-charge equilibration algorithm (see SI), α is the polarizability volume and AIC and bIC are parameters of the Born-Mayer function (eq 8).

### Training of Force Field Parameters

The open source ACT software^13,62^ was used to train the parameters η (atomic hardness), χ (electronegativity), Δ*η*(bond hardness), and Δχ­(difference in electronegativity between bonded atoms) of the SQE algorithm^63,64^ (for details, see Section S3). In addition, when e.g., Gaussian distributed charges are used,^36^ the distribution widths ζ are trained, and for the case of four-site water models the position of the virtual site on the bisector is trained as well. Where appropriate, the magnitude of the charge on shells and virtual sites and the atomic polarizabilities α were part of the training, as described above. Table 2 lists features of the models derived in this work. For the nonpolarizable PC, GC and SC models, a three particle (3P) water model was generated whereas for the PC+GV4 and the polarizable PC+GS4 model, we added a virtual site to the bisector (4P), to make it comparable to TIP4P-Ew^65^ and SWM4-NDP.^66^

In the polarizable model PC+GS4 an additional attractive potential was included to represent the higher order induction effects as proposed by McDaniel and Schmidt,^67^ according to

8 $$V_{\mathrm{ic}}\left(r\right)=-A_{\mathrm{ic}}e^{-b_{\mathrm{ic}}r}$$

where ic indicates “induction correction” and *A* and *b* are positive parameters. The original implementation^67^ combined this potential with a Born-Mayer potential for the exchange, and the same *b*_ic_. In this manner, the induction correction term just modifies the repulsion. However, it is not obvious whether the combination of polarization and this purely attractive term accurately represents the induction energy^13^ and this requires further studies, in particular since polarizability is not necessarily isotropic and constant.^68–70^ In the present implementation, the term in eq 8 effectively becomes an “error sink” that may reduce the root-mean-square error in training, but without clear physical interpretation, leading to potential overfitting.^71^

A hybrid Genetic/Monte Carlo algorithm (GA/MC) was used with a population size of 512 for the polarizable model and 256 for nonpolarizable models. Twenty generations were used in the GA and 40 MC iterations were used per generation at a “temperature” of 0.01.^62^ A penalty function with a force constant of 20,000/*e* ^2^ was used to prevent “unchemical” charges, such as hydrogen atoms with a negative charge. Using this penalty term speeds up convergence of the training. Table 3 lists what training targets were used: either “ESP” (electrostatic potential), “Elec” (SAPT electrostatics), “Elec+Induc” (the sum of electrostatic and induction energies) or “Elec,Induc” which embodies simultaneous training of parameters on both electrostatic and induction, without summing the energies. The latter strategy will lead to a compromise where both energy components are reproduced well, but not as well as when training on “Elec” only. The resulting charges and ζ after training for the models are given in Tables S2–S17.

**Table 3:** Root Mean Square Deviation (RMSD) and Mean Signed Error (MSE), Both in kJ/mol of Electrostatic Energies (Elec) and the Sum of Electrostatics and Induction (Elec+Induc) for Popular Charge Models Compared to ACT Models Based on ESP or SAPT (Table S6)­^a^

	training	#P	Elec		Elec+Induc	
model	target		RMSD	MSE	RMSD	MSE
Existing Charge Models						
Mulliken^17^		45	26 (28)	9.3 (8)	43 (52)	26 (32)
Hirshfeld^20^		45	25 (24)	13 (11)	44 (50)	29 (34)
ESP^73^		45	17 (17)	6.3 (5.7)	36 (45)	23 (29)
CM5 ^21^		45	18 (19)	7.8 (6.6)	38 (46)	24 (30)
BCC^54^		45	18 (17)	4.1 (4)	35 (44)	21 (28)
RESP^25^		45	17 (17)	6.4 (5.8)	37 (45)	23 (29)
MBIS^59^		45	18 (17)	3 (3)	35 (43)	19 (27)
MBIS-S^59^		135	12 (8.6)	–1.5 (−1.1)	26 (36)	15 (22)
Nonpolarizable ACT Monomer-Based Models						
PC+GV3x	ESP	135	**13** (23)	**1.1** (−2.3)	29 (42)	18 (21)
PC+GV4x	ESP	137	**11** (18)	**1.4** (−0.4)	29 (42)	18 (23)
PC+SV3x	ESP	135	**13** (12)	**4.4** (4.1)	33 (43)	21 (28)
PC+SV4x	ESP	137	**16** (16)	**5.7** (4.8)	36 (44)	22 (28)
Nonpolarizable ACT Dimer-Based Models						
PC	Elec	66	**17** (17)	**5.6** (5)	36 (45)	22 (29)
PC	Elec+Induc	66	21 (23)	1 (1)	**34** (45)	**17** (25)
GC	Elec	85	**14** (17)	**3** (3.8)	32 (46)	19 (27)
GC	Elec+Induc	85	22 (26)	–4.4 (−3.8)	**27** (39)	**12** (20)
SC	Elec	85	**16** (18)	**4.6** (4.4)	34 (44)	21 (28)
SC	Elec+Induc	85	23 (27)	–3.2 (−1.7)	**29** (41)	**13** (22)
PC+GV4	Elec	106	**3** (4.5)	**0.1** (−0.9)	23 (33)	17 (23)
PC+GV4	Elec+Induc	106	22 (30)	–14 (−17)	**7.8** (14)	**2.8** (6.3)
PC+SV4	Elec	106	**3.6** (4.8)	**-0.4** (−1)	23 (34)	16 (23)
PC+SV4	Elec+Induc	106	23 (30)	–15 (−18)	**7.3** (14)	**1.5** (5.2)
Polarizable ACT Dimer-Based Models						
PC+GS4	Elec,Induc	156	**3.3** (5.2)	**0.2** (−0.9)	**6.1** (12)	**2.1** (2.5)
PC+GS4	Elec+Induc	156	11 (12)	3.2 (2.9)	**4.8** (11)	**0.9** (1.4)

a Values in brackets are for the test set (Table S6) and #P indicates the number of parameters in the model. Values corresponding to the training targets are indicated in **bold font**.

## Results

In what follows, we first analyze the accuracy of electrostatic energy predictions that can be obtained by fitting charge models to the electrostatic potential of monomeric compounds. We then demonstrate the power of machine learning for deriving accurate models using dimer energies from SAPT. Finally, we provide a detailed breakdown of how training on dimers can be used to derive and evaluate models of increasing precision.

### Analytical Models to Reproduce ESP for Inorganic Ions

To investigate the theoretical accuracy of models for the ESP, we turn to the example of monatomic ions, for which analytical treatments are possible (see Methods section). Figure 2A shows that a single PC is not sufficient to reproduce the ESP for halide ions. A comparable, albeit smaller, discrepancy is observed for alkali ions (Figure 2B). Figure S1 shows the residual ESP, that is ESP from quantum chemistry (QC) minus that of four analytical models, fitted to either the QC ESP from 0 to 4.5 Å (ESP_0_) or from 2 to 4.5 Å (ESP_vdw_, see Methods section) The most complex of these models, consisting of a PC with 1S+2S Slater distributed charges, had the lowest fitting RMSD from the QC ESP (Tables S18 and S19). Based on the monomer fits to QC ESP, electrostatic energies were computed as a function of distance for ion-pairs (Figures S2–S10). As expected, the ESP_0_ model produces somewhat better electrostatic interactions at short distances compared to SAPT than the ESP_*vdw*_ model, while ESP_*vdw*_ does better on distances it was trained on. However, most energy curves are both qualitatively and quantitatively incorrect as compared to SAPT. Interaction energies based on point charges are plotted for comparison in Figures S2–S10 as well, but they have very high RMSD with respect to SAPT. Tables S20 and S21 list electrostatic interaction energies for ion-pairs at their respective minimum energy distance from SAPT for the ESP_0_ and ESP_*vdw*_ fits, respectively. The most complex ESP_vdw_ model yields a RMSD of 8 kJ/mol with respect to SAPT0 (see Methods section) and a mean signed error (MSE) of 2 kJ/mol, whereas the ESP_0_ model basically reverts to the accuracy of interacting PCs, both with an RMSD of 35 kJ/mol and a MSE of ≈14 kJ/mol. Although more data are used in the case of ESP_0_, the fit is more challenging because of the large range of energies, and the resulting model yields less accurate energies at the distance corresponding to the energy minimum (Tables S20 and S21). Finally, Figure S11 shows residual plots of the electrostatic interaction energy for one of the ion-pair models fitted to ESP, highlighting the enormous deviations from reference energies.

**Figure 2:**
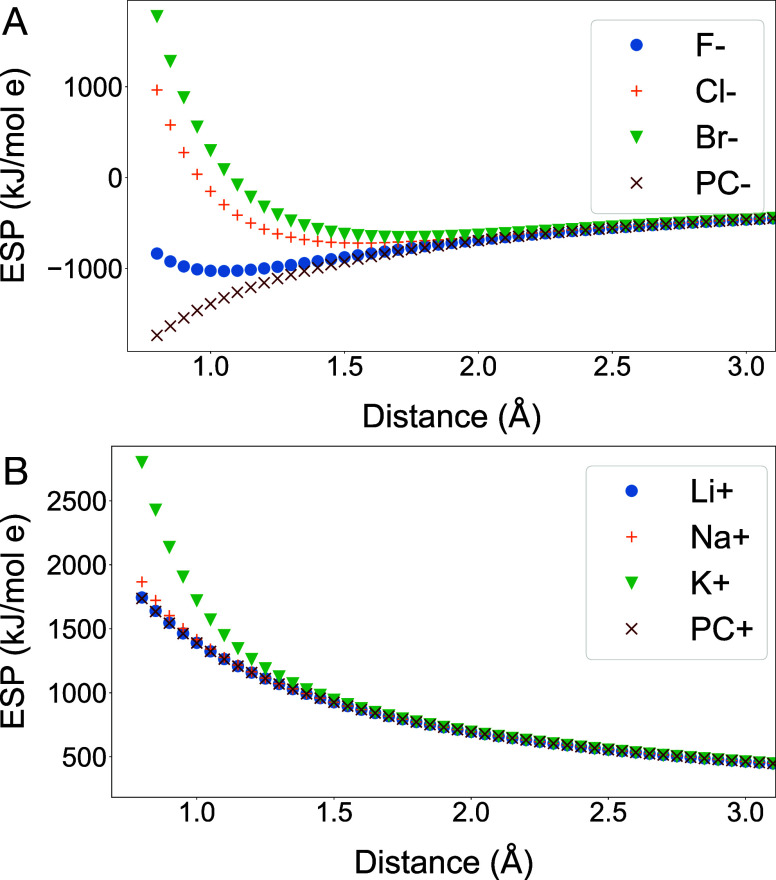
Electrostatic potential (ESP) based on HF/aug-cc-pVTZ calculations (see Methods section) as a function of distance from (A) halide ions and (B) alkali ions. PC denotes the ESP due to a point charge demonstrating that a PC yields a quantitatively (and for anions even qualitatively) incorrect ESP.

### Accuracy of Charge Models from ESP Fitting

To establish how accurately point charges reproduce the molecular electrostatic potential, we used all 5100 compounds from the Alexandria library^57^ (see Methods section). Figure 3 shows the distribution of root-mean-square deviations of point-charge based ESPs from the Hartree–Fock level of theory. The average root-mean-square deviation (RMSD) for the compounds in Figure 3 is 5.7 kJ/mol e, corresponding to an average RMSD in the electrostatic interaction energy with a point charge of 5.7 kJ/mol. Note that this error estimate holds for particles in the distance range used for fitting. At shorter range, the error is likely significantly larger, due to the fact that point charges do not approximate atomic charge distributions well.^33^

**Figure 3:**
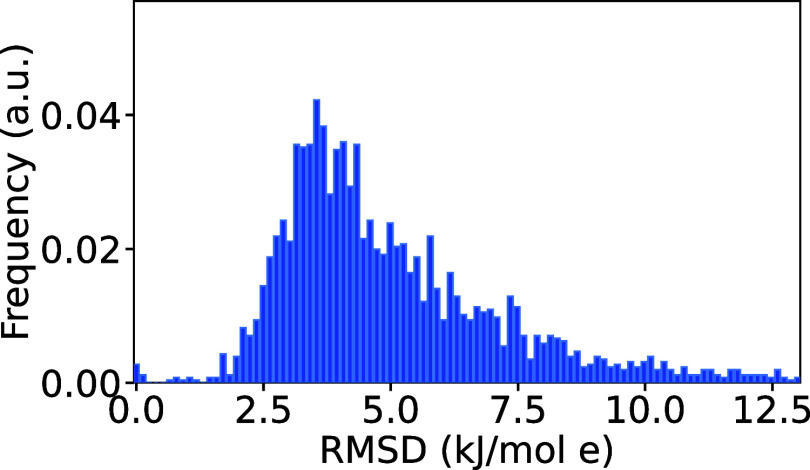
Distribution of the root-mean-square deviation from the fit of point charges to the electrostatic potentials (ESP) in the Alexandria Library.^57^ In brief, the ESP was computed at the HF/6–311G** level of theory for 5100 compounds using the Gaussian software,^72^ and charge fits to ESP were performed using Gaussian as well. The RMSD values were taken directly from the output files and the average RMSD is 5.7 kJ/mol e.

The method of fitting charges to the electrostatic potential can be applied to models including virtual sites or shells.^36^ Here, we used the point charge (PC) + Gaussian virtual site^33^ or PC + 1S Slater virtual site (Table 2). The distribution widths ζ and the charges *q*_V_ on the virtual sites were trained using the ACT (see Methods section), and at each generation in the training algorithm the charges were refitted to the ESP. In this manner, the total deviation from the ESP was minimized with respect to ζ and *q*_V_. The RMSD from ESP for compounds used in this work (Table 1) is given in Table S22 for all models considered. As could be expected, the models combining a PC and a distributed charge yield somewhat lower RMSD than just point charges, and models with TIP4P-like water are more accurate than with TIP3P-like water (except for PC+SV4x, due to interaction between anions, see Figure S14). To evaluate these models, interaction energies were computed for our complete data set of dimers (Table S14). By including a more realistic description of the chemistry of the atoms, the RMSD for these models is reduced by about 30% compared to an ESP based point charges.

The results for ESP-based methods in Table 3 demonstrate that electrostatic interactions are not reproduced well, even though it is possible to make the models somewhat more accurate by adding distributed charges. The deviations from SAPT electrostatics are largest at short distances (Figures S2–S10), which obviously are important for quantitatively determining, for instance, ligand-binding energies. In fact, point-charge models for lithium salts yield qualitatively incorrect electrostatic energies but a PC+GV model trained on the ESP does not fare much better (Figure S11). Likewise, Tables S19 and S18 show large deviations from SAPT electrostatic energies from ESP-derived models, using two different ranges of ESP grid points. Only the most complex model, consisting of a point charge combined with a 1S and a 2S Slater charge, performs significantly better than a single point charge. In summary, there are serious theoretical and practical issues with using ESP fitting, and therefore we present an alternative route for building electrostatics models in what follows.

### Machine Learning to Reproduce Electrostatic and Induction Interactions

Since predictions of electrostatic interactions based on monomer properties using either ESP (Figures S2–S10 and Tables S20,S21) or indeed most other methods^59^ do not yield very good accuracy, we have used machine learning to train physics-based models for electrostatics and induction. For this purpose, we developed the Alexandria Chemistry Toolkit (ACT), which can use a number of algorithms to efficiently derive force field components from scratch.^13^ Here, parameters for the SQE model,^63,64,74^ which generates charge distributions, are trained to reproduce electrostatic energies from SAPT2+(CCD)-δMP2 calculations for six different models (Table 2). The SQE models generated in this manner are, in principle, transferable to compounds containing the same chemical moieties as those present in the training set. After training, no further quantum chemistry calculations are needed to support new compounds, and the transferability of the resulting parameters can be evaluated directly during training using the test set (Table S1). The large SAPT data set presented in this work (see Methods section) can be used both for training and evaluation of models, and we will start with the latter.

#### Accuracy of Electrostatic Interactions of Existing Models

Table 3 gives the deviation from SAPT electrostatic energies for more or less widely used, monomer-based models for our data set (see Methods section). Perhaps not surprisingly, the ESP, CM5, BCC, and RESP methods yield lower RMSDs compared to SAPT than either Mulliken or Hirshfeld. However, a root-mean-square deviation of ≈17 kJ/mol for the ESP model (Table 3) remains large. Indeed, all point charge (PC) models fail to reproduce strong interactions between halide ions and water, acetate or ethylammonium (Table S1, Figures S12 and S13) due to the fact that a PC is a poor representation of the electron density around anions (Figures 2A). The Minimal Basis Iterative Stockholder (MBIS) method^59^ was applied to obtain two further sets of charges, using the MBIS model point charges or point charges plus Slater valence widths (MBIS-S model, see Methods section). The MBIS charges produce a similar deviation from the SAPT reference as the ESP or CM5 methods. MBIS-S has about a 30% lower RMSD than MBIS and the other single charge model (Table 3). The lower RMSD from the MBIS-S model manifests itself in particular for anions (Figure S13), supporting the notion that point charges are a poor model for anions (Figure 2A). This limitation can, however, can be alleviated by modeling a negatively charged atom by a point charge and a distributed charge.^33,59^

#### Accuracy of Models Trained on Dimer Energy Components

The finding that monomer-based models yield limited accuracy for electrostatic interaction energy raises the question whether it is possible to train nonpolarizable models to better reproduce electrostatic and induction energies using the ACT. Table 3 gives the RMSD and MSE for a model with PCs, and similar models with Gaussian (GC) or Slater (SC) distributed charges. These models were trained to reproduce either only the electrostatic energies (Elec) or the sum of the electrostatic and induction energy components (Elec+Induc) from SAPT. The electrostatic interactions with the PC model, based on machine-learned SQE parameters, trained to reproduce electrostatics interactions, show a RMSD of 17 kJ/mol (Training set), similar to ESP, CM5, BCC, RESP, and MBIS. The GC model performs somewhat better, with an RMSD of 14 kJ/mol and somewhat higher for the test set. Interestingly, replacing the Gaussian charges by Slater charges makes the RMSD somewhat higher at 16 kJ/mol. In two further nonpolarizable models (PC+GV4, PC+SV4), virtual sites (see Table 2) were added with either a Gaussian- or a Slater-distributed charge.^33,59^ These models have an 80% lower RMSD for electrostatics than the PC model, demonstrating that there is room for substantial improvement in nonpolarizable models (Figure S1). Here too, the Slater-based model has somewhat higher RMSD than the Gaussian-based model. The monomer-based MBIS-S and the ESP-trained models with two charges per atom have a RMSD that is four times higher than the dimer-based models that share the same computational complexity. These results demonstrate, first, that machine learning using the ACT is an effective strategy to parametrize charge models of varying complexity to reproduce interaction energy components and, second, that models trained on dimer energies or energy components are much more accurate than models trained on monomers.

Figures S14–S15 show heatmaps corresponding to the RMSD from the SAPT electrostatic energies for the models derived here. The electrostatic interaction energies predicted by our PC+GV4 and PC+GS4 models align much closer with the SAPT electrostatic energies than interactions based on ESP^24^ (Figure S12) or MBIS-S^59^ (Figure S13). The information in these heatmaps can be used to pinpoint issues, incomplete physics, or lack of data in the models, yielding a route to systematic improvement of force fields.^75^ For reference, per-dimer RMSDs are tabulated in Table S1. Charges and ζ for a subset of the models are given in Tables S2–S17. Reassuringly, the 1S Slater distribution widths for the MBIS-S model are quite similar to those derived by training the very similar PC+SV4 model on dimer data. However, except for hydrogen atoms, the charges in the PC+SV4 are quite a bit lower than MBIS-S.

#### Including Induction and Polarization in Models

To model the condensed phase, nonpolarizable models need to incorporate the effect of induction implicitly.^76^ Table 3 therefore also compares the sum of electrostatic and induction energy components from SAPT with the PC-based energies. In this comparison, all established models sport a RMSD of more than 35 kJ/mol, and they systematically overestimate the sum of electrostatic and induction energies (large and positive MSE). Including the induction energy in the training of the SQE parameters (Elec+Induc, Table 3) that govern the partial charges leads to a high RMSD for the electrostatic energies compared to SAPT because charges become significantly larger in absolute terms in that case (Tables S2–S17). Despite the larger charges, the electrostatic interactions are underestimated for these models (as demonstrated by a negative MSE in Table 3). To make effective nonpolarizable models, it is therefore needed to train the complete model, including exchange and dispersion at once.^13^

In a final step, the virtual sites in the PC+GV4 model were made polarizable, generating the model PC+GS4 (Table 2). In contrast, when the model was trained to reproduce the sum of the terms, the RMSD goes down for the sum, but up for the components due to compensation of errors between the two terms. In all cases, inspection of the RMSDs from the test sets suggests some overfitting occurred. For further fine-tuning, Table S1 lists the RMSD values with respect to SAPT for selected methods in Table 3, separately for each compound dimer studied (see also Figures S12–S15). Notably, transitioning from PC to PC+GV4 or PC+GS4 yields electrostatic energies that are more consistent with SAPT, particularly for side-chains, ion–water and water–ion interactions, as well as ion pairs.

### Comparison with Other Models

#### Water and Inorganic Ions

A comparison of electrostatic interaction energies from SAPT with some water–ion force field models is given in Figure 4. In particular, the TIP4P-Ew^65^ in combination with point charges for ions, as well as MBIS-S and SWM4-NDP^66^ in combination with CHARMM Drude polarizable ion parameters,^77^ are compared to the PC+GV4 and PC+GS4 models for electrostatics derived here using ACT. The new models exhibit RMSDs that are significantly lower than those of the previously published models (Table S23). Interestingly, the nonpolarizable TIP4P-Ew/PC model predicts cation-water electrostatic interactions that are too strong, likely due to the effective charges on water, and anion-water interactions that are too weak due to the PC description of halides (Figure 2A). The water ion electrostatics modeled using the SWM4-NDP water model and the CHARMM Drude polarizable ion model are too weak for all dimers except water-Li^+^, which maybe related to the fact that the SAPT electrostatic energies for dimers involving Li^+^ are qualitatively different from other ions. The use of a Gaussian virtual site in addition to a point charge (or a shell particle for PC+GS4) makes the ACT models more accurate than the existing models (Table S23) as explained in detail previously.^33^

**Figure 4:**
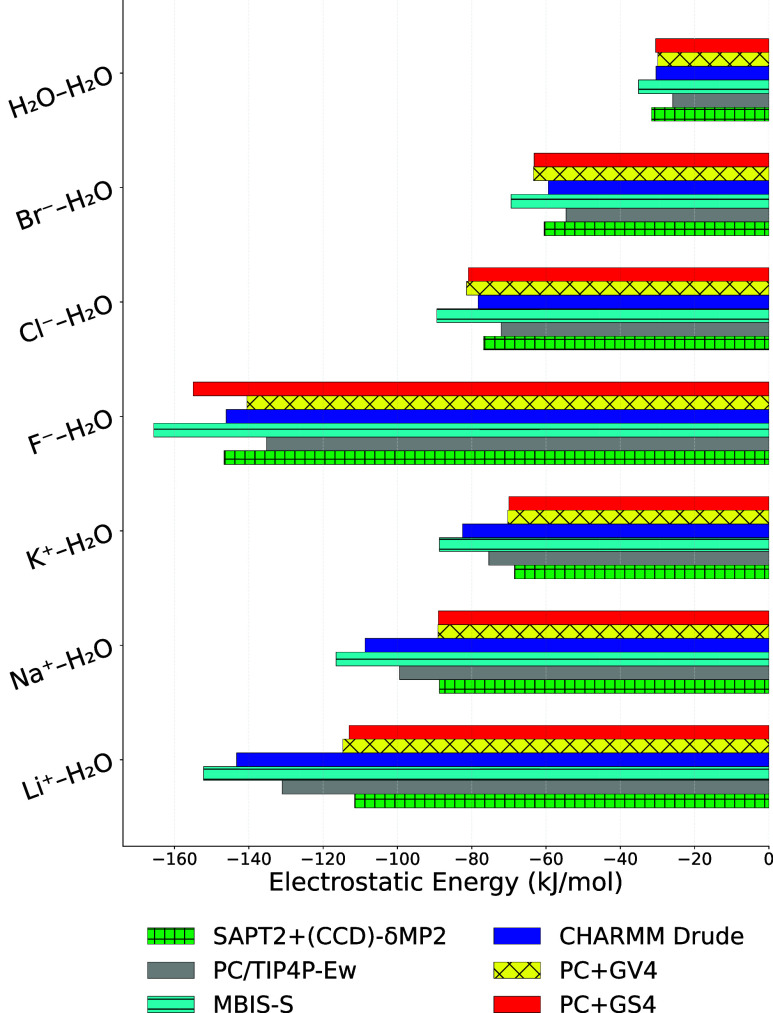
Electrostatic energy close to the minimum energy distances for ion–water electrostatic interactions from SAPT and various models from Table 3. Numerical data and statistics is given in Table S23.

In Figure 5, SAPT electrostatic energies for interacting ions at the minimum energy distance are compared to point charges, MBIS-S, the empirical polarizable model of Walz et al.,^78^ and the models PC+GV4 and PC+GS4. The charge distributions generated by the ions in the Walz et al. model yield a weaker electrostatic interaction energy than PCs, not stronger as it should be according to the SAPT reference calculations. Although the Walz et al. model was successful in predicting properties such as structure^79^ and melting points^80^ of alkali-halide crystals as well as conductivity of molten salts,^81,82^ the two new models are more accurate at predicting electrostatic interaction energies. The main reason for this difference is that the Walz et al. model employs identical GC distribution widths ζ for core and shell particles and that the model incorporates a significant amount of error compensation between energy terms.

**Figure 5:**
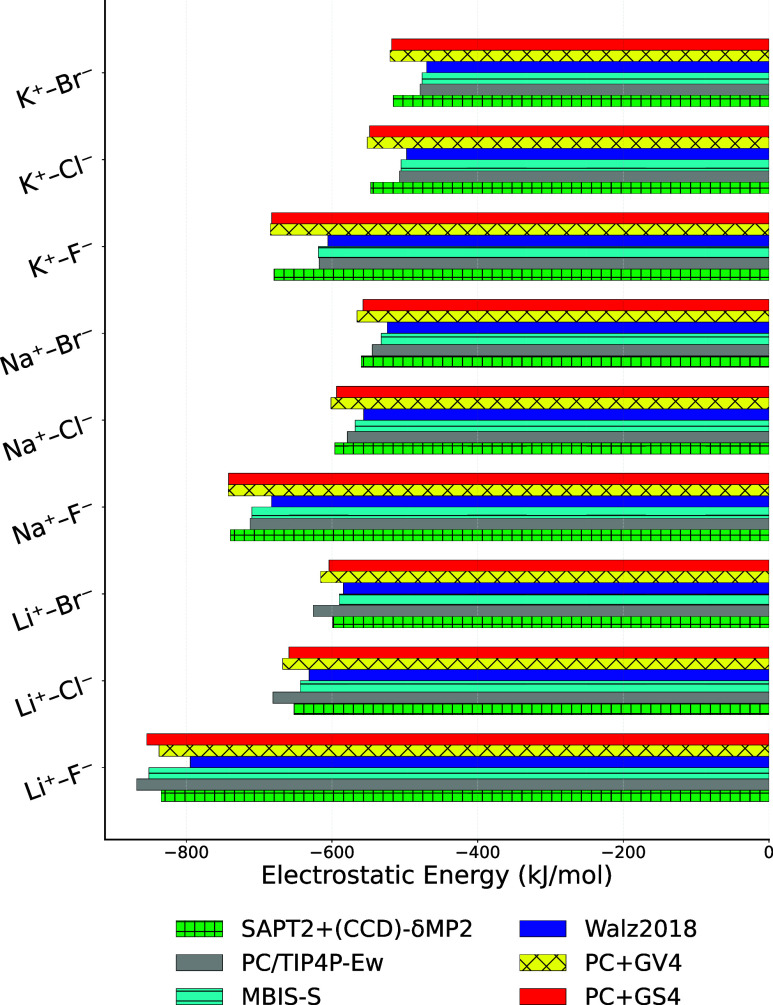
Electrostatic energy close to the minimum energy distances for ion–ion interactions from SAPT and various models from Table 3. Numerical data and statistics is given in Table S24.

We then proceeded to calculate the induction energies for the PC+GS4 model and for SWM4-NDP in combination with CHARMM Drude polarizable ion parameters for interactions between water and inorganic ions. Figure 6 shows the residuals of the induction energy predicted by these models compared to SAPT2+(CCD)­δMP2. Using dimer-based parameters for polarizability and charges, the PC+GS4 model achieves induction energies somewhat closer to QM, although there still is room for improvement (Figure 7 and Table. S25). It should be noted that the PC+GS4 model incorporates an induction correction term (eq 8 ^67^), which may contribute to more accurate induction as well.

**Figure 6:**
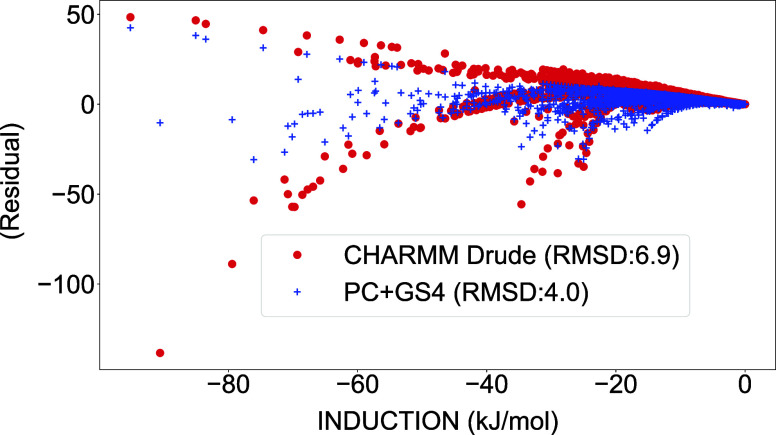
Residual plot of induction energies from the CHARMM Drude polarizable water^66^ and ion^77^ models and the PC+GS4 model, with respect to the SAPT2+(CCD)­δMP2/aug-cc-pVTZ level of theory.

**Figure 7:**
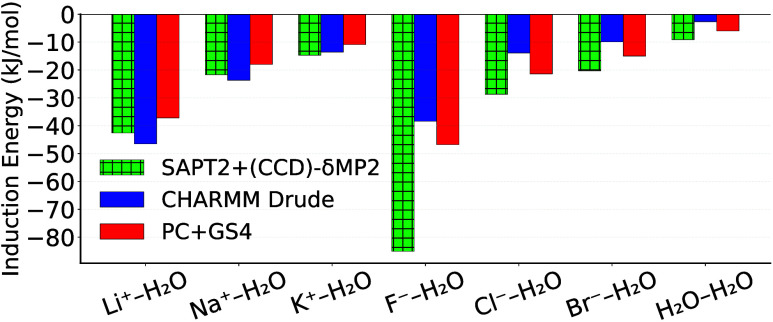
Induction energy for ion–water interactions at their respective energy minima from SAPT, the CHARMM Drude polarizable water^66^ and ion models^77^ and the PC+GS4 model. For statistics, see Table S25.

#### Interactions with Amino Acid Side-Chain Analogs

Finally, we return to the interactions of protein side chain analogs and ions or water (Figure 1). Table S26 gives electrostatic energies at relatively short distances (see Methods section). Here, the electrostatic interaction energies from the PC+GV4 and PC+GS4 models are compared to that based on charges generated by either the Bond Charge Correction algorithm (BCC^54^), the restrained electrostatic potential (RESP^25^) algorithm, both of which are used routinely for the parametrization of GAFF models,^49^ as well as the MBIS-S model.^59^ The PC+GV4 and PC+GS4 models are considerably more accurate than the existing models: they have lower RMSD values as well as mean signed errors (MSE) close to zero. The MBIS-S model yields a RMSD that lies between those obtained for RESP and BCC, and the dimer-derived ACT models with, interestingly, a MSE close to zero as well.

## Discussion

The goal of designing intermolecular potentials is to accurately predict interactions, for instance in drug design^83^ or materials design.^84^ This requires physical models that faithfully reproduce reference data, such as those obtained from SAPT, from databases of quantum chemical calculations^58^ and, ultimately, from experiments. In this work, we focus on electrostatic and induction interactions that govern biological systems,^1–4,7–11^ and we designed a data set consisting of charged amino acid side chain analogs, water, and inorganic ions (Table S1) to base the design of new models on. We demonstrated that the widely used method for determining partial charges by fitting to the ESP^24^ is hampered by a lack of information, leading to interaction energies that are quantitatively and qualitatively incorrect (Figures S2–S10). In fact, most methods to determine partial point charges based on monomeric compounds have been shown to yield predictions of electrostatic interaction energies that are not sufficiently accurate.^59^ This may apply to some extent to advanced models, such as the Gaussian electrostatic model,^85^ the AMOEBA force field^29^ or the component-based force field (CLIFF^32^). The latter model is based on MBIS, with 1S Slater charges and a multipole expansion. Seeing that MBIS-S yields a much larger RMSD for electrostatic interactions for our (admittedly harsh) data set (Table 3) than our models trained on dimers, it seems unlikely that including multipoles on atoms will bring the RMSD down to levels comparable with, e.g., the PC+GV4 model.

The results in Tables 3 and S26 reinvigorate the notion that by transitioning from PC to GC, or even better the PC+GV4 model, nonpolarizable empirical force fields can be made more accurate at a low to moderate computational cost. Interestingly, the point charge + Slater-distributed charge model (PC+SV4), which is used in a number of models,^31,32^ does not perform better than the PC+GV4 model in our analysis (Table 3), despite being more computationally costly. Nevertheless, force fields based on point-charges and a simple van der Waals potential can produce condensed-phase properties of substances reasonably well, and software tools are available that can produce Lennard-Jones plus point-charges models with relative ease.^86,87^ However, such models rely on heavy compensation of errors between energy terms,^13,88^ limiting the transferability of those force fields.^28^ Indeed, point charges do not provide an accurate description of short-range interactions (Figure S1) and do not reproduce the ESP accurately either (Figure 3). It is interesting to note, however, that due to advancements in experimental techniques for measuring electrostatic potentials, the ESP could still be valuable for validating theoretical models.^89^

The BioFragment Database (BFDb) is a data set created from fragments of high-resolution Protein Data Bank structures, for which energies were computed at the SAPT2+/aug-cc-pVDZ level of theory.^90^ Burns et al. compared the total SAPT energies to empirical force fields for amino acid fragments, and found a mean absolute error of 23 kJ/mol for backbone interactions and 11 kJ/mol for side chain interactions. They also found that GAFF severely overestimates interactions involving anions (RMSD > 80 kJ/mol).^90^ These numbers are in line with our findings, since it is difficult in particular to incorporate induction in PC models (Table 3). Since Burns et al. compare total energies predicted by GAFF to SAPT, compensation of errors between the energy components is included in the RMSD values reported. However, compensating, e.g., Coulomb energy by exchange, leads to incorrect forces as these energy components have inherently different distance dependencies.^28^

Owing to the capability of SAPT^27,46,47^ and similar techniques^91^ to provide component-based energies, several research groups have undertaken efforts to develop force fields by training on SAPT components. For instance, the aforementioned MASTIFF model^30,31^ was trained in part on SAPT energies, and Amoeba force fields were evaluated against it.^29^ In addition, Schriber et al. used the SAPT-based BFDb^90^ to validate their component-based force field, CLIFF, which combines physics-based equations for intermolecular interaction energies with machine-learning models to enable automatic parametrization.^32^ This model applies a monomer-based description of electrostatics, but some damping parameters are trained on SAPT dimers. The authors note that their model is limited to neutral dimers, and extending it to charged systems would require major changes to the parametrization, machine learning models, and possibly the functional forms. In contrast, the results presented in this work show that accurate models for electrostatic interactions can be derived purely from dimer-based energies for charged amino acid analogs, inorganic ions, and water (Table 3). Parameters for induction can be trained in the same manner, but more work is needed to improve the models (Figure 6). The electrostatic parameters (charges, Gaussian/Slater distribution widths, positioning of virtual sites) strongly influence the induction term in the force field calculation. This implies that it is beneficial to train the electrostatic parameters in the force field at the same time as the parameters related to induction. It would be interesting to implement such a training scheme for models including atomic multipoles as well.^29,59,92^

The ESP fitting method is hampered by a lack of information, since the ESP at short distances is ignored in practice when fitting charges. As a result, ESP fitted charges do not reproduce electrostatic interactions well for the data set used here. Although much more detailed monomer-based models exist,^29,59,92^ monomer-based methods to derive electrostatic models in general are indirect. We have shown here that deriving models directly from only dimer electrostatics and induction energies from SAPT is a feasible alternative that produces electrostatic energies with chemical accuracy (RMSD < 1 kcal/mol). It is also possible to train models on the induction energies from SAPT by including explicit polarization (Table 3). Although dimer training is also limited by the available data and the physical model used, SAPT provides direct information about interaction energies, the prediction of which is the main purpose of force field calculations. To improve the electrostatics and induction components in force fields beyond what is described here, it may be necessary to involve a more detailed description of the physics, including charge transfer and fluctuations^41^ and accurate induction models.^67,70,93,94^ Developing a complete force field is beyond the scope of this work, but it can be mentioned that anisotropy,^30,32,35,70^ and perhaps many-body effects^31,95^ need to be considered when developing dispersion and exchange models. The tools for systematic force field development^75^ in the ACT can aid in this endeavor by generating new force fields based on different physical models from scratch.^13^

## Supplementary Material



## Data Availability

All scripts, algorithms, and force field files for widely used models, PC, GC, SC, PC+GV, PC+SV and PC+GS models, SAPT calculations, and HF QM calculations to reproduce all the data and plots are available on GitHub.^50^

## Notes

Figures of electrostatic potentials, electrostatic energies from different charge models, SAPT electrostatic energies, and tables including the comparison of electrostatic energies between SAPT2+(CCD)­δMP2, widely used models, and PC, GC, SC, PC+GV4, PC+SV4 and PC+GS4 models, selection data for training, and partial charges and distribution widths from training are available in tables (PDF)
