# Supplementary material for: Beyond Partitioning: Using Force Field Science to Evaluate Electrostatics Models
Source: J Chem Theory Comput. 2026 Feb 21;22(5):2220–31. doi: 10.1021/acs.jctc.6c00039 (PMC12980722; doi:10.1021/acs.jctc.6c00039)
Supplement: Supplementary file 1 [file ct6c00039_si_001.pdf]

Supporting Information: Beyond Partitioning: Using Force Field Science to Evaluate Electrostatics Models

A. Najla Hosseini, Kristian Kříž, and David van der Spoel*

Department of Cell and Molecular Biology, Uppsala University, Husargatan 3, Box 596, SE-75124, Uppsala, Sweden

E-mail: david.vanderspoel@icm.uu.se

Contents

List of Tables **S2**

List of Figures **S6**

S1 Charge Equilibration with Shells or Virtual Sites **S10**

S2 Algorithm for Computing Energy Components **S13**

S3 Numerical analysis of ESP fitting **S15**

S4 Charges for compounds in ACT models **S23**

S5 Interaction energies from ESP-based models **S56**

S6 Interaction energies from ACT-models **S59**

References **S65**

List of Tables

S1 Root mean square deviation (kJ/mol) from SAPT2+(CCD)δMP2 electrostatics per compound dimer for some of the ACT models and widely-used models. N is the number of conformations of each dimer used. Compound dimers used in training are printed in **bold font**. S17

S2 Partial charges q (e) and screening widths ζ (1/nm) for ammonium from ESP, MBIS-S and ACT models. First line, atom, second line (X) corresponds to shell (PC+GS4) or virtual site (other models). S24

S3 Partial charges q (e) and screening widths ζ (1/nm) for methylammonium from ESP, MBIS-S and ACT models. First line, atom, second line (X) corresponds to shell (PC+GS4) or virtual site (other models). S25

S4 Partial charges q (e) and screening widths ζ (1/nm) for ethylammonium from ESP, MBIS-S and ACT models. First line, atom, second line (X) corresponds to shell (PC+GS4) or virtual site (other models). S26

S5 Partial charges q (e) and screening widths ζ (1/nm) for formate from ESP, MBIS-S and ACT models. First line, atom, second line (X) corresponds to shell (PC+GS4) or virtual site (other models). S27

S6 Partial charges q (e) and screening widths ζ (1/nm) for acetate from ESP, MBIS-S and ACT models. First line, atom, second line (X) corresponds to shell (PC+GS4) or virtual site (other models). S28

S7 Partial charges q (e) and screening widths ζ (1/nm) for propanoate from ESP, MBIS-S and ACT models. First line, atom, second line (X) corresponds to shell (PC+GS4) or virtual site (other models). S29

S8 Partial charges q (e) and screening widths ζ (1/nm) for butanoate from ESP, MBIS-S and ACT models. First line, atom, second line (X) corresponds to shell (PC+GS4) or virtual site (other models). S30
S9 Partial charges q (e) and screening widths ζ (1/nm) for guanidinium from ESP, MBIS-S and ACT models. First line, atom, second line (X) corresponds to shell (PC+GS4) or virtual site (other models). S31
S10 Partial charges q (e) and screening widths ζ (1/nm) for imidazolium from ESP, MBIS-S and ACT models. First line, atom, second line (X) corresponds to shell (PC+GS4) or virtual site (other models). S32
S11 Partial charges q (e) and screening widths ζ (1/nm) for water from ESP, MBIS-S and ACT models. First line, atom, second line (X) corresponds to shell (PC+GS4) or virtual site (other models). S33
S12 Partial charges q (e) and screening widths ζ (1/nm) for lithium-ion from ESP, MBIS-S and ACT models. First line, atom, second line (X) corresponds to shell (PC+GS4) or virtual site (other models). S34
S13 Partial charges q (e) and screening widths ζ (1/nm) for sodium-ion from ESP, MBIS-S and ACT models. First line, atom, second line (X) corresponds to shell (PC+GS4) or virtual site (other models). S35
S14 Partial charges q (e) and screening widths ζ (1/nm) for potassium-ion from ESP, MBIS-S and ACT models. First line, atom, second line (X) corresponds to shell (PC+GS4) or virtual site (other models). S36
S15 Partial charges q (e) and screening widths ζ (1/nm) for fluoride from ESP, MBIS-S and ACT models. First line, atom, second line (X) corresponds to shell (PC+GS4) or virtual site (other models). S37
S16 Partial charges q (e) and screening widths ζ (1/nm) for chloride from ESP, MBIS-S and ACT models. First line, atom, second line (X) corresponds to shell (PC+GS4) or virtual site (other models). S38

S17 Partial charges q (e) and screening widths ζ (1/nm) for bromide from ESP, MBIS-S and ACT models. First line, atom, second line (X) corresponds to shell (PC+GS4) or virtual site (other models). S39
S18 ESP parameters for ions and charge models (CM), charge on core q_c and shells q_s *i* and *ii*, respectively, distribution widths ζ in 1/Å. Charge models include a positive point charge with either one Gaussian (PC+G) or 1S Slater distributed charge (PC+1S), and a point charge with two Gaussian charges (PC+G+G), or a point charge with a 1S and a 2S Slater charge (PC+1S+2S). Root mean square error (kJ/mol e) after fitting from 0.0 to 4.5 Å. S50
S19 ESP parameters for ions and charge models (CM), charge on core q_c and shells q_s *i* and *ii*, respectively, distribution widths ζ in 1/Å. Charge models include a positive point charge with either one Gaussian (PC+G) or 1S Slater distributed charge (PC+1S), and a point charge with two Gaussian charges (PC+G+G), or a point charge with a 1S and a 2S Slater charge (PC+1S+2S). Root mean square error (kJ/mol e) after fitting from 2.0 to 4.5 Å. S51
S20 Electrostatic energies at the experimental minimum energy distance[S1] based on the SAPT0[S2] level of theory. Point charge (PC) energy follows from Coulomb's law. ESP indicates model consisting of a point charge, combined with A: a Gaussian charge, B: a 1S Slater charge, C: two Gaussian charges or D: a 1S and a 2S Slater charge fitted to the Hartree-Fock electrostatic potential from 0.0 to 4.5 Å. S56
S21 Electrostatic energies at the experimental minimum energy distance[S1] based on the SAPT0[S2] level of theory. Point charge (PC) energy follows from Coulomb's law. ESP indicates model consisting of a point charge, combined with A: a Gaussian charge, B: a 1S Slater charge, C: two Gaussian charges or D: a 1S and a 2S Slater charge fitted to the Hartree-Fock electrostatic potential from 2.0 to 4.5 Å. S57

S22 RMSD (kJ/mol e) with respect to the electrostatic potential computed at the MP2/aug-cc-pvtz level of theory (see Methods) for the side chain analogs in Table 1 and water. For description of the different models and training, see Methods. S58
S23 Water-ion electrostatic energies at distances close to their energy minimum. Distance r (Å) between ions and water oxygen/hydrogen from Experiment (ref. S3), and minimized water dimer (ref. S4). Electrostatic energies are reported in kJ/mol from the SAPT2+(CCD)-δMP2 method with an aug-cc-pVTZ basis set, TIP4P-Ew[S5] with point charges representing ions, for MBIS-S,[S6] for the CHARMM drude model of water (SWM4-NDP[S7]) with ions due to Yu *et al.*,[S8] as well as point core+Gaussian vsite (PC+GV4), and point charge + Gaussian shell (PC+GS4) derived here using ACT. S59
S24 Ion-pair electrostatic energies at distances close to their energy minimum. Distance r (Å) between ions and electrostatic energies from the SAPT2+(CCD)δMP2/aug-cc-pVTZ level of theory, for point charges (PC), for MBIS-S,[S6] for the Walz *et al.* model with a Gaussian charge distribution,[S9] and the ACT models PC+GV4 and PC+GS4 (see Methods). The RMSD and MSE were calculated with respect to the SAPT2+(CCD)δMP2 with the aug-cc-pVTZ basis set electrostatic energy. Note that this level of theory is different from Tables S18 and S19 and the results cannot be compared directly. In addition, the results in Tables S18 and S19 are from fitting models to the ESP, whereas in this table training was done on SAPT data as indicated above. S60

S25 Water-ion induction energies at distances close to their energy minimum. Distance r (Å) between ions and water oxygen/hydrogen from Experiment (ref. S3), and minimized water dimer (ref. S4). Induction energies are reported in kJ/mol from the SAPT2+(CCD)-δMP2 method with an aug-cc-pVTZ basis set, for the CHARMM drude model of water (SWM4-NDP[S7]) with ions due to Yu *et al.*,[S8] as well the point charge + Gaussian shell (PC+GS4) derived here using ACT. S60

S26 Electrostatic energy (kJ/mol) between alkali ions, halides or water (oxygen) and amino acid side chain analogs, formate (oxygen), acetate (oxygen), methylammonium (nitrogen), ethylammonium (nitrogen) from SAPT2+(CCD)δMP2/aug-cc-pVTZ, and charges determined using either RESP[S10] or BCC[S11] as well as two models generated using the ACT. S61

List of Figures

S1 Electrostatic potentials from analytical fitting to the ESP using different charge models, a positive point charge with either one Gaussian (PC+G) or 1S Slater (PC+1S) distributed charge, and a point charge with two Gaussian charges (PC+G+G), or a point charge with a 1S and a 2S Slater charge (PC+1S+2S), from 2.0 to 4.5 Å (left) and 0.0 to 4.5 Å (right), minus the ESP from quantum chemistry. Note different units on y-axis. S16

S2 Electrostatic energies from SAPT0 with the aug-cc-pVTZ basis set and **four different charge models** based on fitting the ESP from 2.0 to 4.5 Å (left) and 0.0 to 4.5 Å (right) for LiF. Note that units on the y-axis may differ between plots. RMSD is computed for the points plotted, but it should be noted the range of points does not correspond to the range used for fitting, for those numbers please see Tables S1 and S2. . . . . S40

S3 Electrostatic energies from SAPT0 with the aug-cc-pVTZ basis set and **four different charge models** based on fitting the ESP from 2.0 to 4.5 Å (left) and 0.0 to 4.5 Å (right) for LiCl. Note that units on the y-axis may differ between plots. RMSD is computed for the points plotted, but it should be noted the range of points does not correspond to the range used for fitting, for those numbers please see Tables S1 and S2. . . . . S41

S4 Electrostatic energies from SAPT0 with the aug-cc-pVTZ basis set and **four different charge models** based on fitting the ESP from 2.0 to 4.5 Å (left) and 0.0 to 4.5 Å (right) for LiBr. Note that units on the y-axis may differ between plots. RMSD is computed for the points plotted, but it should be noted the range of points does not correspond to the range used for fitting, for those numbers please see Tables S1 and S2. . . . . S42

S5 Electrostatic energies from SAPT0 with the aug-cc-pVTZ basis set and **four different charge models** based on fitting the ESP from 2.0 to 4.5 Å (left) and 0.0 to 4.5 Å (right) for NaF. Note that units on the y-axis may differ between plots. RMSD is computed for the points plotted, but it should be noted the range of points does not correspond to the range used for fitting, for those numbers please see Tables S1 and S2. . . . . S43

S6 Electrostatic energies from SAPT0 with the aug-cc-pVTZ basis set and **four different charge models** based on fitting the ESP from 2.0 to 4.5 Å (left) and 0.0 to 4.5 Å (right) for NaCl. Note that units on the y-axis may differ between plots. RMSD is computed for the points plotted, but it should be noted the range of points does not correspond to the range used for fitting, for those numbers please see Tables S1 and S2. . . . . S44

S7 Electrostatic energies from SAPT0 with the aug-cc-pVTZ basis set and **four different charge models** based on fitting the ESP from 2.0 to 4.5 Å (left) and 0.0 to 4.5 Å (right) for NaBr. Note that units on the y-axis may differ between plots. RMSD is computed for the points plotted, but it should be noted the range of points does not correspond to the range used for fitting, for those numbers please see Tables S1 and S2. . . . . S45
S8 Electrostatic energies from SAPT0 with the aug-cc-pVTZ basis set and **four different charge models** based on fitting the ESP from 2.0 to 4.5 Å (left) and 0.0 to 4.5 Å (right) for KF. Note that units on the y-axis may differ between plots. RMSD is computed for the points plotted, but it should be noted the range of points does not correspond to the range used for fitting, for those numbers please see Tables S1 and S2. . . . . S46
S9 Electrostatic energies from SAPT0 with the aug-cc-pVTZ basis set and **four different charge models** based on fitting the ESP from 2.0 to 4.5 Å (left) and 0.0 to 4.5 Å (right) for KCl. Note that units on the y-axis may differ between plots. RMSD is computed for the points plotted, but it should be noted the range of points does not correspond to the range used for fitting, for those numbers please see Tables S1 and S2. . . . . S47
S10 Electrostatic energies from SAPT0 with the aug-cc-pVTZ basis set and **four different charge models** based on fitting the ESP from 2.0 to 4.5 Å (left) and 0.0 to 4.5 Å (right) for KBr. Note that units on the y-axis may differ between plots. RMSD is computed for the points plotted, but it should be noted the range of points does not correspond to the range used for fitting, for those numbers please see Tables S1 and S2. . . . . S48
S11 Residual electrostatic interactions computed using point charges and using the P+G model fitted to the ESP in the 2.0–4.5 Å range with respect to SAPT0 for nine ion-pairs. S49

S12 Heatmaps for root mean square deviation of electrostatic energies from SAPT results for existing models. S52
S13 Heatmaps for root mean square deviation of electrostatic energies from SAPT results for existing models and ACT models. S53
S14 Heatmaps for root mean square deviation of electrostatic energies from SAPT results for ACT models derived from ESP fitting of charges and ζ. S54
S15 Heatmaps for root mean square deviation of electrostatic energies from SAPT results for ACT models derived by training on dimer energies. S55
S16 Residual plots for electrostatic interactions of existing models with respect to the SAPT2+(CCD)δMP2 for all compounds. A) Mulliken,[S27] B) Hirshfeld,[S28] C) CM5,[S29] D) BCC,[S11] E) ESP[S30] and F) RESP.[S10] S62
S17 Residual plots for electrostatic interactions of MBIS and MBIS-S models as well as ACT-trained on ESP with respect to the SAPT2+(CCD)δMP2 for all compounds. A) MBIS,[S6] B) MBIS-S,[S6] C-F models derived in this work based on ESP fitting (see Table 2). S63
S18 Residual plots for electrostatic interactions of ACT-trained models with respect to the SAPT2+(CCD)δMP2 for all compounds. A-F model derived in this work based on training the split-charge equilibration method[S16,S17] on dimer energies (see Table 2). S64

S1 Charge Equilibration with Shells or Virtual Sites

Among the approaches to modeling the charge-dependent component of a force field, those rooted in the chemical potential equalization principle are especially notable, as this principle stems directly from density functional theory.[S12] The first computational implementation of the chemical potential equalization principle was the electronegativity equalization method (EEM).[S13,S14] However, due to limitations of this model, Chelli *et al.* proposed the atom-atom charge transfer (AACT) model.[S15] Later, Nistor and co-workers combined the EEM and AACT approaches into a single framework, the split-charge equilibration (SQE) model, which fulfills the essential criteria for a successful charge-transfer potential.[S16,S17] The ACT implements both the EEM and the SQE as algorithms for determining partial charges.

In brief, EEM minimizes an empirical model of the intramolecular electrostatic energy (computed from the atomic electronegativity χ_i and atomic hardness η_i) with respect to the atomic partial charges q_i, where the i denote the atoms. This method comprises a second-order expansion of the molecular energy E_{EEM} in terms of the partial charges q_i:

$$E_{\text{EEM}}(q_1, q_2, \ldots, q_N) = \sum_{i=1}^{N} \left[\chi_i q_i + \frac{1}{2}\eta_i q_i^2 + \frac{1}{2} \sum_{\substack{l=1 \\ l \neq i}}^{N} q_i q_l J_{il} \right], \tag{S1}$$

where N is the number of atoms, χ_i are the atomic electronegativities, η_i the atomic hardness, and J_{il} the Coulomb interaction between atoms. The factor $\frac{1}{2}$ before the Coulomb matrix avoids double counting.

In this work, the SQE method is used, which addresses a shortcoming of the EEM, namely that molecules tend to become over-polarized.[S16] For more background, we refer to the recent review on charge flow models by Jensen.[S18]

Verstraelen and co-workers proposed the following variant of the molecular energy:

$$E_{\text{SQE}} = E_{\text{EEM}} + \sum_{i,j}^{M} \left(\frac{1}{2}\Delta\eta_{ij} p_{ij}^2 + \Delta\chi_{ij}(q_i - q_j) \right) \tag{S2}$$

where p_{ij} corresponds to the (intramolecular) charge transfer over bonds, $\Delta\eta_{ij}$ is the bond hardness, and $\Delta\chi_{ij}$ is the bond electronegativity correction. Therefore, the charge variables q_i are replaced by charge-transfer variables p_{ij} which are related by

$$q_i = \frac{q_{\text{tot}}}{N} + \sum_{\substack{i,j \\ \text{bonds}}} p_{ij}, \tag{S3}$$

where q_{tot} is the net charge on the compound and $p_{ij} = -p_{ji}$. Although it is trivial to determine the partial charges q_i from the charge transfer p_{ij}, the reverse is not necessarily true. As outlined by Chen *et al.*,[S19] the problem can be solved by expressing the energy in terms of the charge transfer variables. By substituting $J_{ii} = \eta_i$ in Eq. S1, inserting Eq. S1 into Eq. S2 and introducing M_x as the number of bonds for species x, we obtain:

$$\begin{aligned} E_{\text{SQE}} = \sum_{n=1}^{N} \Bigg[&\left(\frac{q_{\text{tot}}}{N} + \sum_{m=1}^{M_n} p_{nm}\right)\left(\chi_n + \frac{1}{2}\eta_n\left(\frac{q_{\text{tot}}}{N} + \sum_{m=1}^{M_n} p_{nm}\right)\right. \\ &\left.+ \frac{1}{2}\sum_{\substack{l=1 \\ l\neq n}}^{N}\left(\frac{q_{\text{tot}}}{N} + \sum_{m=1}^{M_l} p_{lm}\right) J_{nl}\right)\Bigg] \\ &+ \sum_{i,j}^{M}\left[\frac{1}{2}\zeta_{ij}p_{ij}^2 + \Delta\chi_{ij}\left(\left(\frac{q_{\text{tot}}}{N} + \sum_{m=1}^{M_i} p_{im}\right) - \left(\frac{q_{\text{tot}}}{N} + \sum_{m=1}^{M_j} p_{jm}\right)\right)\right]. \end{aligned} \tag{S4}$$

The next step is to determine the values of p_{ij} that minimize E_{SQE}. Since all summations run over atoms i, j, k, l, we take the derivative with respect to p_{ij} and equate it to zero:

$$\begin{aligned} 0 &= \frac{\partial E_{\text{SQE}}}{\partial p_{ij}} \\ &= \left(\chi_i - \chi_j + \frac{q_{\text{tot}}}{N}(\eta_i - \eta_j) + \sum_{k=1}^{M_i}\Delta\chi_{ik} - \sum_{k=1}^{M_j}\Delta\chi_{jk}\right) \\ &\quad + \frac{1}{2}\left(\sum_{l=1}^{N} J_{il}\left(\frac{q_{\text{tot}}}{N} + \sum_{m=1}^{M_l} p_{lm}\right) - \sum_{l=1}^{N} J_{li}\left(\frac{q_{\text{tot}}}{N} + \sum_{k=1}^{M_i} p_{ik}\right)\right) + p_{ij}\zeta_{ij}, \end{aligned} \tag{S5}$$

using the identity of the Coulomb-matrix elements ($J_{ij} = J_{ji}$), the terms involving the atomic

hardness η_x are incorporated into the diagonals of the J_{xy} matrix, excluding the contribution from the total charge q_{tot}. Note that the q_{tot} terms in the two sums cancel. The first term in Eq. S5 is the difference in electronegativity between atoms i and j sharing the bond plus the correction; the second term represents the difference in electrostatic potentials at the atoms, and the third term accounts for the interaction between i and j times the charge transfer. This results in a coupled set of equations, written in matrix form as

$$\mathbf{M}\,\mathbf{P} = \mathbf{R}, \tag{S6}$$

where $\mathbf{M}$ is a square matrix of dimension equal to the number of bonds, $\mathbf{P}$ is the vector of the charge transfers for all bonds, and $\mathbf{R}$ is the right-hand side of the equations. The matrix elements are given by

$$M_{ij,kl} = J_{ik} - J_{il} - J_{jk} + J_{jl} + \delta_{ij,kl}\Delta\eta_{ij} \tag{S7}$$

where $\delta_{ij,kl}$ is one if bond ij is identical to kl and zero otherwise. The right-hand side is defined by the electronegativity terms according to

$$R_{ij} = \chi_j - \chi_i + \sum_{k=1}^{M_j} \Delta\chi_{jk} - \sum_{k=1}^{M_i} \Delta\chi_{ik} + \frac{q_{\text{tot}}}{N}\left(\sum_{l=1}^{N} J_{jl} - \sum_{l=1}^{N} J_{il}\right). \tag{S8}$$

The charges of the shells (and virtual sites) are treated as constant in this algorithm, meaning that q$_{tot}$ becomes the sum of the charges of the shells and virtual sites and the total charge of the compound. During force field training, all of these charges can be modified alongside the SQE parameters. As noted in the paper describing the ACT software,[S20] the SQE algorithm may not be flexible enough to reproduce optimal charge distributions, and other algorithms[S18,S21] may need to be implemented in future versions of the software.

S2 Algorithm for Computing Energy Components

The components of the interaction energy of a dimer can be computed from the difference between the dimer and the monomers A and B:[S22]

$$E_x^{inter}(AB) = E_x^{total}(AB) - (E_x(A) + E_x(B)) \tag{S9}$$

where x denotes electrostatics (or exchange or dispersion) and $total$ indicates that the energy includes both the intra- and intermolecular interactions. To compute the electrostatics and induction energy components of a dimer in the gas phase, the ACT first computes the relaxed energy of the two monomers A and B, that is, the energy of the shell particles is minimized with respect to their positions, yielding $E_x(A)$ and $E_x(B)$. The rationale for this is that SAPT computes electrostatic energies between unperturbed "frozen" monomers based on the response and relaxation of monomer Hartree-Fock (HF) orbitals in the electric field of the interacting partner.[S23] Then, the energies of the dimer AB are computed in three steps:

1. the electrostatic interaction energy is computed with shells located in the relaxed monomer positions, yielding $E_{elec}^{total}(AB)$,

2. the shells of compound A are allowed to relax (further) in the electric field from compound B, while shells of compound B remain at their monomer positions, and vice versa, yielding the second-order relaxation $E_{induc}^{inter(2)}$ (see ref. S24 for details),

3. the shells are allowed to relax completely, yielding the total E_{induc} from which the higher-order terms, named $E_{induc}^{inter(3)}$ here for convenience, can be derived by subtracting $E_{induc}^{inter(2)}$. According to ref. S24, parameters of models corresponding to the higher order terms, including, potentially, charge transfer, can be trained to reproduce the δHF contribution to the SAPT induction energy. Here, we have added the exponential term proposed by McDaniel and Schmidt (Eqn. 8, main text), which is also used in the MASTIFF model[S25,S26] as well, for this purpose.

This then yields the terms below:

$$E_{elec}^{inter}(AB) = E_{elec}^{total}(AB) - (E_{ei}(A) + E_{ei}(B)) \quad \text{(S10)}$$

$$E_{induc}^{inter(2)}(AB) = \left(E_{ei}^{total}\|_A + E_{ei}^{total}\|_B\right) - 2E_{elec}^{total}(AB) \quad \text{(S11)}$$

$$E_{induc}^{inter(3)}(AB) = E_{ei}^{total}(AB) - E_{induc}^{(2)}(AB) - E_{elec}^{total}(AB) \quad \text{(S12)}$$

where ei is short for $elec + induc$ and the notation $\|_A$ or $\|_B$ indicates that the shells of compound A or B are kept fixed in the relaxed monomer conformation. To summarize, Eqn. S10 directly corresponds to the electrostatics term in SAPT, while the induction term consists of two parts: a second-order term $E_{induc}^{inter(2)}(AB)$ (Eqn. S11) and a third-and-higher-order term $E_{induc}^{inter(3)}(AB)$ (Eqn. S12), the sum of which corresponds to the total induction from SAPT.

S3 Numerical analysis of ESP fitting

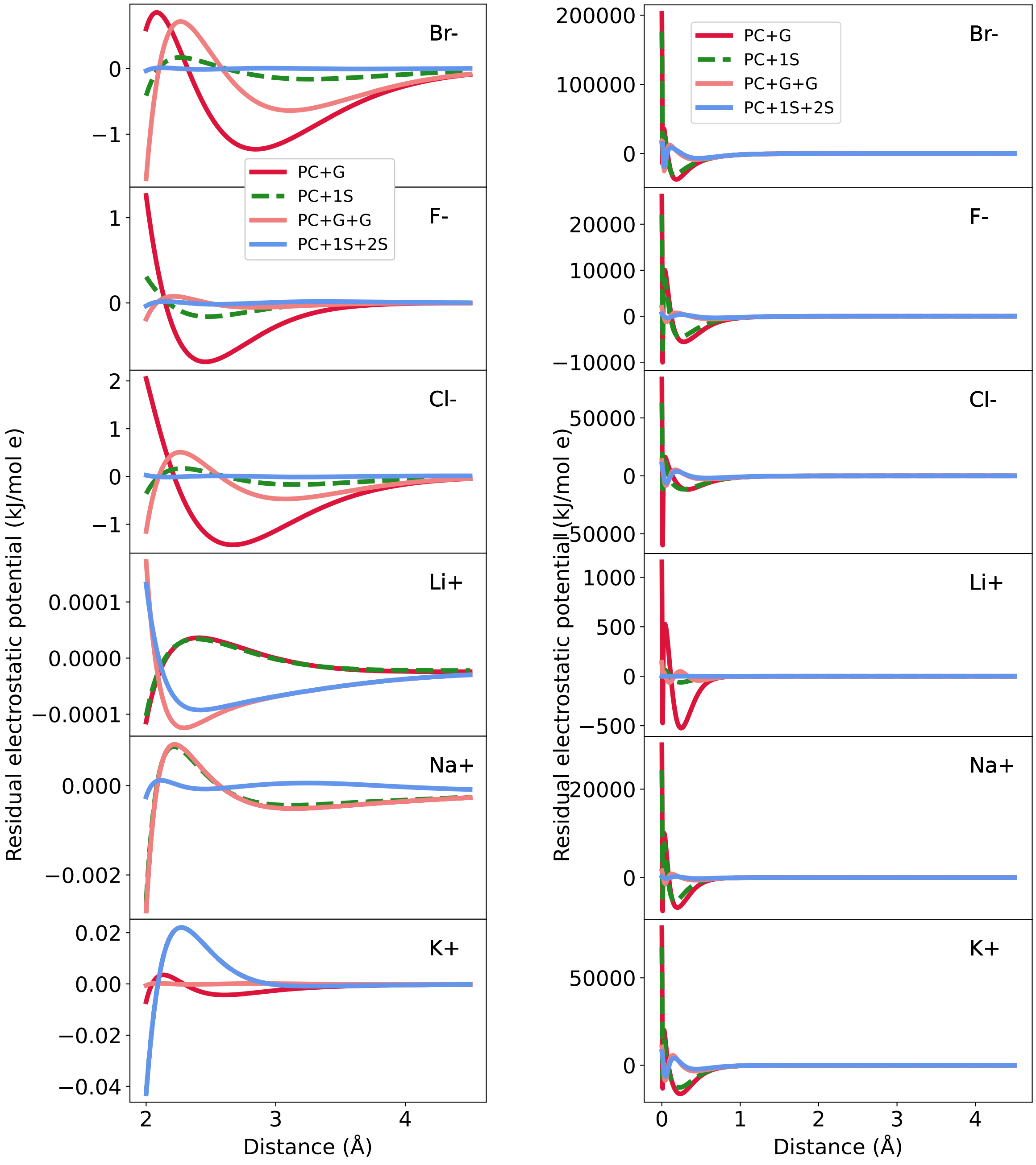

Figure S1: Electrostatic potentials from analytical fitting to the ESP using different charge models, a positive point charge with either one Gaussian (PC+G) or 1S Slater (PC+1S) distributed charge, and a point charge with two Gaussian charges (PC+G+G), or a point charge with a 1S and a 2S Slater charge (PC+1S+2S), from 2.0 to 4.5 Å (left) and 0.0 to 4.5 Å (right), minus the ESP from quantum chemistry. Note different units on y-axis.

Table S1: Root mean square deviation (kJ/mol) from SAPT2+(CCD)δMP2 electrostatics per compound dimer for some of the ACT models and widely-used models. N is the number of conformations of each dimer used. Compound dimers used in training are printed in **bold font**.

Dimer	N	ESP	BCC	RESP	MBIS-S	PC+GV4x	PC+SV4x	PC	GC	SC	PC+GV4	PC+SV4	PC+GS4
acetate-acetate	176	13.8	14.5	13.8	6.8	8.6	8.2	15.3	13.1	9.3	2.5	3.6	2.1
**acetate-bromide**	89	48.2	47.6	47.9	5.7	34.4	18.0	49.2	26.9	43.8	2.3	3.0	1.6
**acetate-chloride**	125	48.5	47.5	48.5	7.1	28.4	27.9	47.0	28.4	42.0	2.9	5.4	2.5
acetate-fluoride	163	53.8	54.6	53.6	23.4	43.9	33.9	56.2	31.1	50.8	3.3	6.4	2.9
acetate-lithium	311	3.7	5.3	3.6	4.5	5.1	4.5	7.9	40.7	22.1	5.2	3.2	3.2
**acetate-potassium**	251	18.9	16.9	19.0	16.7	15.6	19.9	16.4	21.4	19.1	2.3	2.9	2.7
acetate-sodium	279	6.8	5.9	6.8	7.0	7.8	8.2	6.9	20.7	16.3	3.7	3.5	3.7
acetate-water	251	14.7	12.7	14.7	11.0	5.2	7.2	13.6	14.1	15.0	2.5	3.2	2.8
**ammonium-acetate**	180	11.8	12.5	11.8	6.1	11.6	12.2	14.3	12.2	15.1	3.0	4.0	2.9
**ammonium-ammonium**	176	2.6	3.5	2.7	1.2	2.6	2.6	4.3	11.1	13.2	2.3	2.8	2.2
ammonium-bromide	286	14.6	14.6	14.6	5.3	19.3	15.3	14.4	12.7	10.2	4.2	4.7	3.4
ammonium-chloride	232	18.9	18.4	18.9	6.5	29.3	22.8	20.6	18.8	13.8	4.5	6.3	4.2
ammonium-ethylammonium	76	8.1	9.3	8.1	6.1	7.8	7.5	7.7	18.9	24.1	7.0	4.4	11.4

**ammonium-fluoride**	190	18.7	18.6	18.6	4.5	17.7	18.0	17.0	8.6	10.3	3.8	4.1	3.5
**ammonium-formate**	184	19.6	22.3	19.6	11.9	19.6	18.6	22.6	13.8	14.4	7.6	8.3	7.6
ammonium-lithium	120	0.2	0.7	0.3	0.2	0.2	0.2	2.4	1.4	2.4	1.4	1.7	1.2
**ammonium-methylammonium**	69	4.1	3.6	4.2	5.3	3.5	2.3	3.6	9.8	10.4	1.6	1.4	3.0
**ammonium-potassium**	83	0.1	0.5	0.1	0.1	0.1	0.1	1.3	0.7	2.4	0.6	0.4	0.6
ammonium-sodium	75	0.1	0.6	0.1	0.1	0.1	0.1	1.2	0.7	2.1	0.7	0.7	0.6
**ammonium-water**	306	10.4	10.7	10.4	13.2	7.4	7.8	11.3	10.5	10.8	3.1	3.2	3.2
**bromide-bromide**	53	22.1	22.1	22.1	4.7	7.3	6.8	22.1	16.1	22.1	4.3	1.9	2.9
**bromide-chloride**	55	17.6	17.6	17.6	2.1	0.8	6.8	17.6	11.2	17.6	1.7	2.3	1.0
**bromide-fluoride**	61	13.3	13.3	13.3	4.9	6.1	5.3	13.3	8.9	13.2	0.9	1.4	0.9
**chloride-chloride**	58	19.2	19.2	19.2	2.1	5.5	10.1	19.2	11.2	19.2	1.5	2.1	0.9
**chloride-fluoride**	65	14.8	14.8	14.8	5.7	13.3	7.8	14.8	9.0	14.7	0.8	1.3	0.8
**ethylammonium-acetate**	96	26.2	23.1	26.6	5.2	26.4	23.9	25.2	15.9	18.3	5.0	8.1	5.7
ethylammonium-bromide	182	15.0	15.1	15.7	7.7	18.9	16.7	14.8	11.1	8.8	3.2	3.9	4.4
**ethylammonium-chloride**	204	20.5	20.9	20.2	6.6	14.9	19.3	20.2	11.4	11.6	2.6	2.9	3.6
ethylammonium-ethylammonium	29	1.4	2.0	1.6	4.6	1.8	1.6	5.0	9.0	10.1	3.2	3.2	4.1
ethylammonium-fluoride	189	24.9	27.4	25.9	10.6	33.8	22.5	26.9	13.7	16.4	5.7	5.3	8.5
**ethylammonium-formate**	151	23.2	24.3	24.8	12.2	19.6	20.4	20.3	11.1	12.8	4.7	7.2	5.4
**ethylammonium-lithium**	85	1.5	2.7	1.6	6.0	1.5	1.4	2.6	5.0	7.8	3.9	3.2	5.2

**ethylammonium-methylammonium**	43	1.8	2.0	1.8	3.8	1.1	1.9	4.7	4.0	10.2	1.7	1.7	2.7
ethylammonium-potassium	58	1.8	1.4	1.9	2.2	1.7	1.9	2.8	3.4	4.4	3.5	3.1	2.9
**ethylammonium-sodium**	37	0.8	1.3	0.9	2.3	0.8	0.9	3.6	2.3	4.6	2.3	1.9	2.9
**ethylammonium-water**	211	17.6	17.8	17.8	15.4	11.9	14.5	16.8	13.0	16.5	3.2	2.7	2.9
**fluoride-fluoride**	73	14.6	14.6	14.6	9.9	28.0	8.0	14.6	11.5	14.3	0.8	1.1	1.2
**formate-acetate**	75	8.5	11.6	8.5	6.6	5.2	4.7	11.0	8.3	9.3	2.8	2.9	2.7
formate-bromide	44	21.3	23.7	21.3	5.1	4.4	6.6	21.1	16.6	21.9	3.2	4.9	4.9
**formate-chloride**	86	41.1	45.3	41.1	6.5	2.7	18.9	40.1	30.2	40.2	4.5	5.3	6.2
**formate-fluoride**	93	34.8	40.2	34.8	20.4	12.9	17.4	37.5	32.8	40.6	4.6	6.1	4.3
formate-formate	132	19.0	19.2	19.0	8.4	5.1	7.1	18.7	18.6	23.4	4.5	5.8	5.6
formate-lithium	264	8.2	19.0	8.2	14.9	11.5	9.4	10.7	10.7	23.1	11.8	12.9	13.8
**formate-potassium**	214	22.0	20.5	22.0	20.7	16.8	23.9	23.0	24.0	21.4	4.3	4.9	4.1
**formate-sodium**	253	12.8	17.0	12.8	15.3	12.7	14.8	11.5	12.7	9.7	5.4	5.9	5.6
**formate-water**	179	20.7	21.6	20.7	12.8	3.2	8.1	20.4	17.8	20.0	2.4	3.5	3.3
**guanidinium-acetate**	146	7.7	5.9	7.7	2.6	5.6	4.7	6.7	8.9	7.0	2.3	3.4	3.4
**guanidinium-bromide**	60	8.3	6.6	8.3	1.2	4.9	4.5	7.5	1.7	3.0	0.7	2.0	3.8
**guanidinium-chloride**	60	16.5	11.7	16.5	2.1	4.5	7.8	10.9	4.7	4.7	1.0	3.0	4.3
guanidinium-fluoride	60	9.0	6.1	8.9	3.8	3.4	5.5	8.2	3.9	12.5	0.6	4.3	6.3
**guanidinium-formate**	220	13.7	12.0	13.7	4.0	6.5	6.9	11.0	7.8	7.8	3.3	5.1	4.7

**guanidinium-water**	160	5.6	5.6	5.6	6.4	1.8	1.9	5.7	4.4	6.3	1.4	2.2	2.1
**imidazolium-acetate**	140	9.7	10.4	9.6	4.0	7.5	7.5	7.9	7.0	7.9	2.8	3.4	3.1
**imidazolium-bromide**	121	14.2	15.4	14.2	4.1	8.8	8.4	10.6	6.3	7.5	1.8	2.6	3.2
**imidazolium-chloride**	120	12.0	13.8	12.0	4.0	7.2	8.2	8.2	4.6	5.1	1.8	2.2	2.6
**imidazolium-formate**	120	6.0	8.6	6.0	4.9	3.3	3.9	6.5	4.4	3.8	2.9	3.4	2.6
**imidazolium-imidazolium**	4	2.3	3.9	2.3	3.7	1.3	0.9	4.6	3.8	6.2	1.1	2.6	2.2
**imidazolium-water**	200	7.2	7.4	7.2	6.7	4.6	4.1	7.0	5.7	7.0	2.0	2.7	3.4
lithium-bromide	82	17.0	17.0	17.0	7.2	11.0	6.6	17.0	8.8	14.7	6.4	4.3	4.0
lithium-chloride	85	14.5	14.5	14.5	5.7	15.2	3.6	14.5	13.8	10.6	6.0	4.4	3.7
lithium-fluoride	90	9.4	9.4	9.4	5.1	14.3	2.5	9.4	3.3	2.2	4.2	3.9	7.0
**lithium-lithium**	81	0.0	0.0	0.0	0.0	0.0	0.0	0.0	0.0	1.4	0.2	2.0	0.0
lithium-potassium	81	0.2	0.2	0.2	0.2	0.2	0.2	0.2	0.2	1.5	2.1	1.9	1.4
lithium-sodium	81	0.1	0.1	0.1	0.1	0.1	0.1	0.1	0.1	1.3	0.2	0.4	0.1
**methylammonium-acetate**	121	17.9	12.7	18.1	5.8	17.8	10.4	12.7	11.0	9.6	3.0	5.7	3.1
methylammonium-bromide	190	16.5	16.0	16.5	5.0	9.7	7.7	15.9	11.3	14.1	2.5	3.1	3.4
**methylammonium-chloride**	192	16.8	17.3	16.8	6.0	13.8	10.5	17.7	11.7	14.9	2.4	2.9	3.4
methylammonium-fluoride	194	20.7	21.4	20.7	7.0	34.3	13.1	22.0	11.0	20.0	4.9	4.5	6.9
**methylammonium-formate**	88	23.0	23.8	23.0	11.2	20.9	3.2	20.0	13.4	11.1	3.3	5.8	3.2
**methylammonium-lithium**	105	2.5	2.7	2.5	4.4	2.6	2.7	3.5	8.1	9.2	2.0	1.9	3.6

**methylammonium-methylammonium**	141	2.5	4.1	2.4	2.1	1.9	1.5	5.2	5.7	9.2	1.7	1.5	2.9
**methylammonium-potassium**	72	0.9	1.3	0.9	2.0	1.0	1.0	1.2	2.2	2.2	1.0	0.8	1.2
methylammonium-sodium	105	0.8	1.3	0.8	1.6	0.8	0.8	1.9	4.0	3.1	0.8	0.9	1.5
**methylammonium-water**	205	12.2	12.2	12.2	16.3	10.4	4.2	11.4	10.8	11.7	4.1	3.0	3.9
**potassium-bromide**	70	23.9	23.9	23.9	25.9	13.4	27.5	23.9	29.5	24.2	2.7	1.7	2.0
**potassium-butanoate**	20	1.6	4.0	1.5	3.9	2.0	2.2	4.0	5.7	5.3	6.2	7.5	5.6
**potassium-chloride**	72	21.9	21.9	21.9	23.0	8.8	24.9	21.9	27.8	22.3	1.9	1.8	1.5
**potassium-fluoride**	79	21.8	21.8	21.8	21.4	4.3	24.0	21.8	23.2	22.8	1.6	1.6	1.2
**potassium-potassium**	71	2.3	2.3	2.3	2.3	2.3	2.3	2.3	2.3	2.1	0.6	3.4	1.0
**potassium-propanoate**	20	1.4	5.1	2.0	5.3	3.3	3.0	3.9	4.8	5.3	5.7	4.7	5.2
**sodium-bromide**	77	9.8	9.8	9.8	17.9	14.5	16.9	9.8	21.8	10.5	2.0	1.8	5.6
**sodium-butanoate**	20	1.7	4.1	1.8	4.2	2.2	2.4	3.8	6.5	8.0	10.4	10.7	6.0
**sodium-chloride**	78	9.7	9.7	9.7	15.8	12.3	15.6	9.7	22.8	11.0	1.7	1.8	4.5
**sodium-fluoride**	86	10.9	10.9	10.9	12.1	8.5	15.5	10.9	16.2	14.1	2.3	2.5	2.4
**sodium-potassium**	81	3.0	3.0	3.0	3.1	3.0	3.0	3.0	3.0	1.7	0.3	4.7	1.7
sodium-propanoate	20	1.7	4.5	2.9	5.2	3.7	3.6	4.1	7.8	5.8	4.4	6.5	5.2
**sodium-sodium**	81	0.6	0.6	0.6	0.6	0.6	0.6	0.6	0.6	0.8	0.5	1.6	0.4
**water-bromide**	482	22.4	23.0	22.4	8.2	2.9	5.7	22.4	19.2	22.6	1.6	1.9	1.9
**water-chloride**	529	21.5	21.6	21.5	8.4	7.8	7.8	21.3	18.1	21.6	1.6	2.0	1.7

water-fluoride	591	18.8	18.0	18.8	9.8	23.8	6.7	18.4	14.1	19.3	3.1	2.9	3.0
**water-lithium**	563	7.8	11.2	7.8	17.9	4.7	37.2	8.0	11.6	8.2	2.8	2.8	2.6
**water-potassium**	456	7.5	8.8	7.5	13.1	3.4	15.4	7.5	8.5	7.5	1.8	2.8	1.7
**water-sodium**	493	11.0	11.9	11.0	16.4	5.4	26.3	10.8	13.5	11.2	3.3	3.3	3.5
water-water	546	10.9	8.0	10.9	5.6	3.0	31.3	10.1	11.2	11.4	2.5	2.5	2.6

S4 Charges for compounds in ACT models

Table S2: Partial charges q (e) and screening widths ζ (1/nm) for ammonium from ESP, MBIS-S and ACT models. First line, atom, second line (X) corresponds to shell (PC+GS4) or virtual site (other models).

Particle	ESP	PC_e	PC_{ei}	MBIS-S		$PC+SV4_e$		$PC+GV4_e$		$PC+GS4_{e,i}$	
	q	q	q	q	ζ	q	ζ	q	ζ	q	ζ
hn	0.4581	0.242	0.5404	1.0		0.9502		0.9643		1.3613	
hn X				-0.5374	29.51	-0.6239	20.0	-0.6154	14.9	-1.0	17.39
n4	-0.8324	0.0319	-1.1618	5.4085		1.1375		1.111		1.0767	
n4 X				-6.259	21.27	-1.4425	18.58	-1.5069	12.59	-1.5219	17.23
hn	0.4581	0.242	0.5404	1.0		0.9502		0.9643		1.3613	
hn X				-0.5374	29.51	-0.6239	20.0	-0.6154	14.9	-1.0	17.39
hn	0.4581	0.242	0.5404	1.0		0.9502		0.9643		1.3613	
hn X				-0.5374	29.51	-0.6239	20.0	-0.6154	14.9	-1.0	17.39
hn	0.4581	0.242	0.5404	1.0		0.9502		0.9643		1.3613	
hn X				-0.5374	29.51	-0.6239	20.0	-0.6154	14.9	-1.0	17.39
Total	1	1	1	1		1		1		1	

Table S3: Partial charges q (e) and screening widths ζ (1/nm) for methylammonium from ESP, MBIS-S and ACT models. First line, atom, second line (X) corresponds to shell (PC+GS4) or virtual site (other models).

Particle	ESP	PC_e	PC_{ei}	MBIS-S		$PC+SV4_e$		$PC+GV4_e$		$PC+GS4_{e,i}$	
	q	q	q	q	ζ	q	ζ	q	ζ	q	ζ
n4	-0.3553	-1.018	-1.092	5.3942		1.2474		1.2073		1.3751	
n4 X				-6.0134	21.32	-1.4425	18.58	-1.5069	12.59	-1.5219	17.23
c3	-0.0889	0.4835	0.4917	4.3745		1.8014		1.6302		2.2686	
c3 X				-4.542	19.0	-1.8316	16.75	-1.8356	11.04	-1.9387	10.41
hn	0.3427	0.5003	0.554	1.0		0.9166		0.9467		1.2776	
hn X				-0.5734	29.1	-0.6239	20.0	-0.6154	14.9	-1.0	17.39
hn	0.3424	0.5003	0.554	1.0		0.9166		0.9467		1.2776	
hn X				-0.5734	29.1	-0.6239	20.0	-0.6154	14.9	-1.0	17.39
hn	0.3424	0.5003	0.554	1.0		0.9166		0.9467		1.2776	
hn X				-0.5734	29.1	-0.6239	20.0	-0.6154	14.9	-1.0	17.39
h1	0.1389	0.0113	-0.0206	1.0		0.6214		0.6777		0.9947	
h1 X				-0.831	25.74	-0.5056	20.0	-0.5075	13.85	-1.0	19.76
h1	0.1389	0.0113	-0.0206	1.0		0.6214		0.6777		0.9947	
h1 X				-0.831	25.74	-0.5056	20.0	-0.5075	13.85	-1.0	19.76
h1	0.1389	0.0113	-0.0206	1.0		0.6214		0.6777		0.9947	
h1 X				-0.831	25.74	-0.5056	20.0	-0.5075	13.85	-1.0	19.76
Total	1	1	1	1		1		1		1	

Table S4: Partial charges q (e) and screening widths ζ (1/nm) for ethylammonium from ESP, MBIS-S and ACT models. First line, atom, second line (X) corresponds to shell (PC+GS4) or virtual site (other models).

Particle	ESP	PC_e	PC_{ei}	MBIS-S		$PC+SV4_e$		$PC+GV4_e$		$PC+GS4_{e,i}$	
	q	q	q	q	ζ	q	ζ	q	ζ	q	ζ
n4	-0.5657	-1.0144	-1.0575	5.3908		1.3264		1.2358		1.3567	
n4 X				-6.0397	21.21	-1.4425	18.58	-1.5069	12.59	-1.5219	17.23
c3	0.1991	0.3351	0.7309	4.3634		1.4665		1.2891		2.0018	
c3 X				-4.3412	19.09	-1.8316	16.75	-1.8356	11.04	-1.9387	10.41
hn	0.3797	0.5021	0.5497	1.0		0.9055		0.9501		1.3063	
hn X				-0.5748	29.01	-0.6239	20.0	-0.6154	14.9	-1.0	17.39
hn	0.3798	0.5021	0.5497	1.0		0.9055		0.9501		1.3063	
hn X				-0.5748	29.01	-0.6239	20.0	-0.6154	14.9	-1.0	17.39
hn	0.3798	0.5021	0.5497	1.0		0.9055		0.9501		1.3063	
hn X				-0.5748	29.01	-0.6239	20.0	-0.6154	14.9	-1.0	17.39
h1	0.086	0.0697	-0.0103	1.0		0.746		0.8065		1.0851	
h1 X				-0.8559	25.67	-0.5056	20.0	-0.5075	13.85	-1.0	19.76
h1	0.086	0.0697	-0.0103	1.0		0.746		0.8065		1.0851	
h1 X				-0.8559	25.67	-0.5056	20.0	-0.5075	13.85	-1.0	19.76
c3	-0.3962	-0.8029	-1.8517	4.3678		1.5509		1.7161		1.784	
c3 X				-4.8248	18.41	-1.8316	16.75	-1.8356	11.04	-1.9387	10.41
hc	0.1416	0.2789	0.5166	1.0		0.689		0.6364		1.0559	
hc X				-0.8267	25.22	-0.5436	16.75	-0.5248	11.66	-1.0	16.1
hc	0.1683	0.2789	0.5166	1.0		0.689		0.6364		1.0559	
hc X				-0.8267	25.76	-0.5436	16.75	-0.5248	11.66	-1.0	16.1
hc	0.1416	0.2789	0.5166	1.0		0.689		0.6364		1.0559	
hc X				-0.8267	25.22	-0.5436	16.75	-0.5248	11.66	-1.0	16.1
Total	1	1	1	1		1		1		1	

Table S5: Partial charges q (e) and screening widths ζ (1/nm) for formate from ESP, MBIS-S and ACT models. First line, atom, second line (X) corresponds to shell (PC+GS4) or virtual site (other models).

Particle	ESP	PC_e	PC_{ei}	MBIS-S		$PC+SV4_e$		$PC+GV4_e$		$PC+GS4_{e,i}$	
	q	q	q	q	ζ	q	ζ	q	ζ	q	ζ
o2	-0.8027	-0.7245	-0.8521	6.3286		1.6907		1.2165		1.2978	
o2 X				-7.1557	22.62	-2.3689	14.84	-1.8885	9.51	-1.9779	9.27
o2	-0.8027	-0.7245	-0.8521	6.3286		1.6907		1.2165		1.2978	
o2 X				-7.1557	22.62	-2.3689	14.84	-1.8885	9.51	-1.9779	9.27
c2	0.8015	0.5347	0.7973	4.2856		0.8776		0.9171		1.6208	
c2 X				-3.4852	18.99	-0.4568	12.34	-0.5166	7.33	-1.1977	19.0
h2	-0.196	-0.0857	-0.0931	1.0		0.7462		0.6227		0.937	
h2 X				-1.1461	21.49	-0.8105	16.98	-0.6793	11.83	-1.0	12.48
Total	-1	-1	-1	-1		-1		-1		-1	

Table S6: Partial charges q (e) and screening widths ζ (1/nm) for acetate from ESP, MBIS-S and ACT models. First line, atom, second line (X) corresponds to shell (PC+GS4) or virtual site (other models).

Particle	ESP	PC_e	PC_{ei}	MBIS-S		$PC+SV4_e$		$PC+GV4_e$		$PC+GS4_{e,i}$	
	q	q	q	q	ζ	q	ζ	q	ζ	q	ζ
c3	-0.3681	-0.3888	-1.4011	4.3313		1.431		1.4297		1.6226	
c3 X				-5.0771	17.35	-1.8316	16.75	-1.8356	11.04	-1.9387	10.41
c2	0.9539	0.8049	2.0682	4.3188		1.3108		1.4378		2.0181	
c2 X				-3.2721	20.26	-0.4568	12.34	-0.5166	7.33	-1.1977	19.0
o2	-0.8464	-0.8199	-1.3032	6.331		1.5551		1.0481		1.1359	
o2 X				-7.204	22.61	-2.3689	14.84	-1.8885	9.51	-1.9779	9.27
o2	-0.8464	-0.8199	-1.3032	6.331		1.5551		1.0481		1.1359	
o2 X				-7.204	22.61	-2.3689	14.84	-1.8885	9.51	-1.9779	9.27
hc	0.0334	0.0746	0.3131	1.0		0.6017		0.5799		1.0599	
hc X				-0.8516	24.68	-0.5436	16.75	-0.5248	11.66	-1.0	16.1
hc	0.0402	0.0746	0.3131	1.0		0.6017		0.5799		1.0599	
hc X				-0.8516	24.56	-0.5436	16.75	-0.5248	11.66	-1.0	16.1
hc	0.0334	0.0746	0.3131	1.0		0.6017		0.5799		1.0599	
hc X				-0.8516	24.68	-0.5436	16.75	-0.5248	11.66	-1.0	16.1
Total	-1	-1	-1	-1		-1		-1		-1	

Table S7: Partial charges q (e) and screening widths ζ (1/nm) for propanoate from ESP, MBIS-S and ACT models. First line, atom, second line (X) corresponds to shell (PC+GS4) or virtual site (other models).

Particle	ESP	PC_e	PC_{ei}	MBIS-S		$PC+SV4_e$		$PC+GV4_e$		$PC+GS4_{e,i}$	
	q	q	q	q	ζ	q	ζ	q	ζ	q	ζ
o2	-0.8371	-0.7992	-1.2256	6.3311		1.6158		1.0449		1.1437	
o2 X				-7.1703	22.55	-2.3689	14.84	-1.8885	9.51	-1.9779	9.27
o2	-0.7907	-0.7992	-1.2256	6.3311		1.6158		1.0449		1.1437	
o2 X				-7.1703	22.72	-2.3689	14.84	-1.8885	9.51	-1.9779	9.27
c3	0.1604	0.0066	-0.6309	4.344		1.1393		1.0482		1.4031	
c3 X				-4.7406	18.05	-1.8316	16.75	-1.8356	11.04	-1.9387	10.41
c3	-0.1308	-0.834	-1.5763	4.3379		1.8994		1.9846		2.0724	
c3 X				-4.6961	18.01	-1.8316	16.75	-1.8356	11.04	-1.9387	10.41
c2	0.7782	0.7812	1.9349	4.3125		1.3349		1.5354		2.0329	
c2 X				-3.3937	19.78	-0.4568	12.34	-0.5166	7.33	-1.1977	19.0
hc	-0.0811	0.0124	0.2573	1.0		0.6764		0.6875		1.1234	
hc X				-0.8934	24.25	-0.5436	16.75	-0.5248	11.66	-1.0	16.1
hc	-0.0811	0.0124	0.2573	1.0		0.6764		0.6875		1.1234	
hc X				-0.8934	24.25	-0.5436	16.75	-0.5248	11.66	-1.0	16.1
hc	0.0111	0.2066	0.403	1.0		0.5393		0.5185		0.9962	
hc X				-0.8996	24.38	-0.5436	16.75	-0.5248	11.66	-1.0	16.1
hc	0.0111	0.2066	0.403	1.0		0.5393		0.5185		0.9962	
hc X				-0.8996	24.38	-0.5436	16.75	-0.5248	11.66	-1.0	16.1
hc	-0.0399	0.2066	0.403	1.0		0.5393		0.5185		0.9962	
hc X				-0.8996	23.73	-0.5436	16.75	-0.5248	11.66	-1.0	16.1
Total	-1	-1	-1	-1		-1		-1		-1	

Table S8: Partial charges q (e) and screening widths ζ (1/nm) for butanoate from ESP, MBIS-S and ACT models. First line, atom, second line (X) corresponds to shell (PC+GS4) or virtual site (other models).

Particle	ESP	PC_e	PC_{ei}	MBIS-S		$PC+SV4_e$		$PC+GV4_e$		$PC+GS4_{e,i}$	
	q	q	q	q	ζ	q	ζ	q	ζ	q	ζ
c3	-0.1919	-0.0219	-0.715	4.344		1.1068		1.0604		1.3808	
c3 X				-4.8011	17.96	-1.8316	16.75	-1.8356	11.04	-1.9387	10.41
c2	0.8338	0.7765	1.9722	4.3119		1.3382		1.587		2.0379	
c2 X				-3.3866	19.79	-0.4568	12.34	-0.5166	7.33	-1.1977	19.0
o2	-0.815	-0.7993	-1.2535	6.3308		1.6407		1.0521		1.1406	
o2 X				-7.1726	22.69	-2.3689	14.84	-1.8885	9.51	-1.9779	9.27
o2	-0.8276	-0.7993	-1.2535	6.3308		1.6407		1.0521		1.1406	
o2 X				-7.1726	22.56	-2.3689	14.84	-1.8885	9.51	-1.9779	9.27
c3	0.4492	-0.413	-0.7617	4.3509		1.635		1.6049		1.8616	
c3 X				-4.4359	18.67	-1.8316	16.75	-1.8356	11.04	-1.9387	10.41
hc	-0.0097	0.0037	0.2748	1.0		0.7206		0.7552		1.1414	
hc X				-0.8848	24.33	-0.5436	16.75	-0.5248	11.66	-1.0	16.1
hc	-0.0097	0.0037	0.2748	1.0		0.7206		0.7552		1.1414	
hc X				-0.8848	24.33	-0.5436	16.75	-0.5248	11.66	-1.0	16.1
hc	-0.0922	0.1761	0.3597	1.0		0.5548		0.5769		1.0151	
hc X				-0.9107	24.33	-0.5436	16.75	-0.5248	11.66	-1.0	16.1
hc	-0.0922	0.1761	0.3597	1.0		0.5548		0.5769		1.0151	
hc X				-0.9107	24.33	-0.5436	16.75	-0.5248	11.66	-1.0	16.1
c3	-0.4139	-0.776	-1.5138	4.3398		1.8358		1.9145		2.0227	
c3 X				-4.7615	17.95	-1.8316	16.75	-1.8356	11.04	-1.9387	10.41
hc	0.0632	0.2245	0.4188	1.0		0.5822		0.5129		1.0241	
hc X				-0.8957	24.3	-0.5436	16.75	-0.5248	11.66	-1.0	16.1
hc	0.0426	0.2245	0.4188	1.0		0.5822		0.5129		1.0241	
hc X				-0.8957	24.14	-0.5436	16.75	-0.5248	11.66	-1.0	16.1
hc	0.0632	0.2245	0.4188	1.0		0.5822		0.5129		1.0241	
hc X				-0.8957	24.3	-0.5436	16.75	-0.5248	11.66	-1.0	16.1
Total	-1	-1	-1	-1		-1		-1		-1	

Table S9: Partial charges q (e) and screening widths ζ (1/nm) for guanidinium from ESP, MBIS-S and ACT models. First line, atom, second line (X) corresponds to shell (PC+GS4) or virtual site (other models).

Particle	ESP	PC_e	PC_{ei}	MBIS-S		$PC+SV4_e$		$PC+GV4_e$		$PC+GS4_{e,i}$	
	q	q	q	q	ζ	q	ζ	q	ζ	q	ζ
c2	1.0116	0.1564	0.7702	4.3446		0.6066		1.6221		1.2883	
c2 X				-3.2536	21.0	-0.4568	12.34	-0.5166	7.33	-1.1977	19.0
n2	-0.9527	-0.1872	-0.4308	5.3799		1.1885		0.3637		1.0836	
n2 X				-6.3174	20.67	-1.553	17.03	-1.3409	10.02	-1.3483	9.21
n2	-0.9505	-0.1872	-0.4308	5.3799		1.1885		0.3637		1.0836	
n2 X				-6.3174	20.67	-1.553	17.03	-1.3409	10.02	-1.3483	9.21
n2	-0.9537	-0.1872	-0.4308	5.3799		1.1885		0.3637		1.0836	
n2 X				-6.3174	20.67	-1.553	17.03	-1.3409	10.02	-1.3483	9.21
hn	0.4736	0.2342	0.2537	1.0		0.9479		1.0863		1.2839	
hn X				-0.5464	29.28	-0.6239	20.0	-0.6154	14.9	-1.0	17.39
hn	0.4736	0.2342	0.2537	1.0		0.9479		1.0863		1.2839	
hn X				-0.5464	29.28	-0.6239	20.0	-0.6154	14.9	-1.0	17.39
hn	0.4742	0.2342	0.2537	1.0		0.9479		1.0863		1.2839	
hn X				-0.5464	29.28	-0.6239	20.0	-0.6154	14.9	-1.0	17.39
hn	0.4744	0.2342	0.2537	1.0		0.9479		1.0863		1.2839	
hn X				-0.5464	29.28	-0.6239	20.0	-0.6154	14.9	-1.0	17.39
hn	0.4749	0.2342	0.2537	1.0		0.9479		1.0863		1.2839	
hn X				-0.5464	29.28	-0.6239	20.0	-0.6154	14.9	-1.0	17.39
hn	0.4747	0.2342	0.2537	1.0		0.9479		1.0863		1.2839	
hn X				-0.5464	29.28	-0.6239	20.0	-0.6154	14.9	-1.0	17.39
Total	1	1	1	1		1		1		1	

Table S10: Partial charges q (e) and screening widths ζ (1/nm) for imidazolium from ESP, MBIS-S and ACT models. First line, atom, second line (X) corresponds to shell (PC+GS4) or virtual site (other models).

Particle	ESP	PC_e	PC_{ei}	MBIS-S		PC+SV4_e		PC+GV4_e		PC+GS$4_{e,i}$	
	q	q	q	q	ζ	q	ζ	q	ζ	q	ζ
c2	-0.0915	0.1488	0.3884	4.3615		0.4947		0.4272		1.331	
c2 X				-4.3699	19.05	-0.4568	12.34	-0.5166	7.33	-1.1977	19.0
c2	-0.0915	0.1488	0.3884	4.3615		0.4947		0.4272		1.331	
c2 X				-4.3699	19.05	-0.4568	12.34	-0.5166	7.33	-1.1977	19.0
n2	-0.0829	-0.1926	-0.5378	5.3867		1.3086		1.3676		1.2356	
n2 X				-5.7469	21.57	-1.553	17.03	-1.3409	10.02	-1.3483	9.21
c2	-0.0327	0.099	0.6771	4.359		0.514		0.2086		1.244	
c2 X				-4.051	19.59	-0.4568	12.34	-0.5166	7.33	-1.1977	19.0
n2	-0.0829	-0.1926	-0.5378	5.3867		1.3086		1.3676		1.2356	
n2 X				-5.7469	21.57	-1.553	17.03	-1.3409	10.02	-1.3483	9.21
h4	0.2245	0.0842	-0.0042	1.0		1.044		1.0013		1.0414	
h4 X				-0.7951	26.15	-0.8832	20.0	-0.7837	19.93	-1.0	12.21
h4	0.2245	0.0842	-0.0042	1.0		1.044		1.0013		1.0414	
h4 X				-0.7951	26.15	-0.8832	20.0	-0.7837	19.93	-1.0	12.21
hn	0.3527	0.3082	0.2593	1.0		1.0347		0.9423		1.356	
hn X				-0.5776	28.9	-0.6239	20.0	-0.6154	14.9	-1.0	17.39
h5	0.2271	0.2039	0.1114	1.0		0.9673		0.9573		1.1177	
h5 X				-0.8256	25.87	-0.7546	20.0	-0.6129	18.63	-1.0	12.92
hn	0.3527	0.3082	0.2593	1.0		1.0347		0.9423		1.356	
hn X				-0.5776	28.9	-0.6239	20.0	-0.6154	14.9	-1.0	17.39
Total	1	1	1	1		1		1		1	

Table S11: Partial charges q (e) and screening widths ζ (1/nm) for water from ESP, MBIS-S and ACT models. First line, atom, second line (X) corresponds to shell (PC+GS4) or virtual site (other models).

Particle	ESP	PC_e	PC_{ei}	MBIS-S		$PC+SV4_e$		$PC+GV4_e$		$PC+GS4_{e,i}$	
	q	q	q	q	ζ	q	ζ	q	ζ	q	ζ
ow	-0.6783	-0.7059	-0.86	6.3494		4.1067		1.5623		1.8556	
ow X				-7.2541	22.9	-3.7013	19.76	-1.5613	9.74	-1.6799	9.88
hw	0.3391	0.3529	0.43	1.0		1.0736		1.2167		1.5199	
hw X				-0.5476	27.19	-0.5713	19.85	-0.6982	16.94	-1.0	19.45
hw	0.3391	0.3529	0.43	1.0		1.0736		1.2167		1.5199	
hw X				-0.5476	27.19	-0.5713	19.85	-0.6982	16.94	-1.0	19.45
v3bw						-1.4101		-1.0381		-1.2155	
Total	-0	0	0	0		0		-0		-0	

Table S12: Partial charges q (e) and screening widths ζ (1/nm) for lithium-ion from ESP, MBIS-S and ACT models. First line, atom, second line (X) corresponds to shell (PC+GS4) or virtual site (other models).

Particle	ESP	PC_e	PC_{ei}	MBIS-S		PC+$SV4_e$		PC+$GV4_e$		PC+$GS4_{e,i}$	
	q	q	q	q	ζ	q	ζ	q	ζ	q	ζ
Li+	1.0	1.0	1.0	1.076		1.2401		1.5427		2.0	
Li+ X				-0.076	45.1	-0.2401	11.75	-0.5427	13.74	-1.0	18.43
Total	1	1	1	1		1		1		1	

Table S13: Partial charges q (e) and screening widths ζ (1/nm) for sodium-ion from ESP, MBIS-S and ACT models. First line, atom, second line (X) corresponds to shell (PC+GS4) or virtual site (other models).

Particle	ESP	PC_e	PC_{ei}	MBIS-S		PC+SV4_e		PC+GV4_e		PC+GS$4_{e,i}$	
	q	q	q	q	ζ	q	ζ	q	ζ	q	ζ
Na+	1.0	1.0	1.0	1.2063		2.7492		4.3237		2.2233	
Na+ X				-0.2063	37.28	-1.7492	20.0	-3.3237	19.96	-1.2233	13.92
Total	1	1	1	1		1		1		1	

Table S14: Partial charges q (e) and screening widths ζ (1/nm) for potassium-ion from ESP, MBIS-S and ACT models. First line, atom, second line (X) corresponds to shell (PC+GS4) or virtual site (other models).

Particle	ESP	PC_e	PC_{ei}	MBIS-S		$PC+SV4_e$		$PC+GV4_e$		$PC+GS4_{e,i}$	
	q	q	q	q	ζ	q	ζ	q	ζ	q	ζ
K+	1.0	1.0	1.0	1.3349		6.4606		4.8166		3.9797	
K+ X				-0.3349	23.13	-5.4606	20.0	-3.8166	12.55	-2.9797	11.77
Total	1	1	1	1		1		1		1	

Table S15: Partial charges q (e) and screening widths ζ (1/nm) for fluoride from ESP, MBIS-S and ACT models. First line, atom, second line (X) corresponds to shell (PC+GS4) or virtual site (other models).

Particle	ESP	PC_e	PC_{ei}	MBIS-S		PC+SV4_e		PC+GV4_e		PC+GS$4_{e,i}$	
	q	q	q	q	ζ	q	ζ	q	ζ	q	ζ
F-	-1.0	-1.0	-1.0	7.2242		1.9513		0.9041		1.348	
F- X				-8.2242	23.64	-2.9513	15.19	-1.9041	8.91	-2.348	9.52
Total	-1	-1	-1	-1		-1		-1		-1	

Table S16: Partial charges q (e) and screening widths ζ (1/nm) for chloride from ESP, MBIS-S and ACT models. First line, atom, second line (X) corresponds to shell (PC+GS4) or virtual site (other models).

Particle	ESP	PC_e	PC_{ei}	MBIS-S		$PC+SV4_e$		$PC+GV4_e$		$PC+GS4_{e,i}$	
	q	q	q	q	ζ	q	ζ	q	ζ	q	ζ
Cl-	-1.0	-1.0	-1.0	8.1206		5.0999		2.8315		2.3293	
Cl- X				-9.1206	16.03	-6.0999	13.97	-3.8315	8.18	-3.3293	7.86
Total	-1	-1	-1	-1		-1		-1		-1	

Table S17: Partial charges q (e) and screening widths ζ (1/nm) for bromide from ESP, MBIS-S and ACT models. First line, atom, second line (X) corresponds to shell (PC+GS4) or virtual site (other models).

Particle	ESP	PC_e	PC_{ei}	MBIS-S		PC+SV4$_e$		PC+GV4$_e$		PC+GS4$_{e,i}$	
	q	q	q	q	ζ	q	ζ	q	ζ	q	ζ
Br-	-1.0	-1.0	-1.0	8.8016		6.525		3.8415		2.9969	
Br- X				-9.8016	14.82	-7.525	13.38	-4.8415	7.87	-3.9969	7.44
Total	-1	-1	-1	-1		-1		-1		-1	

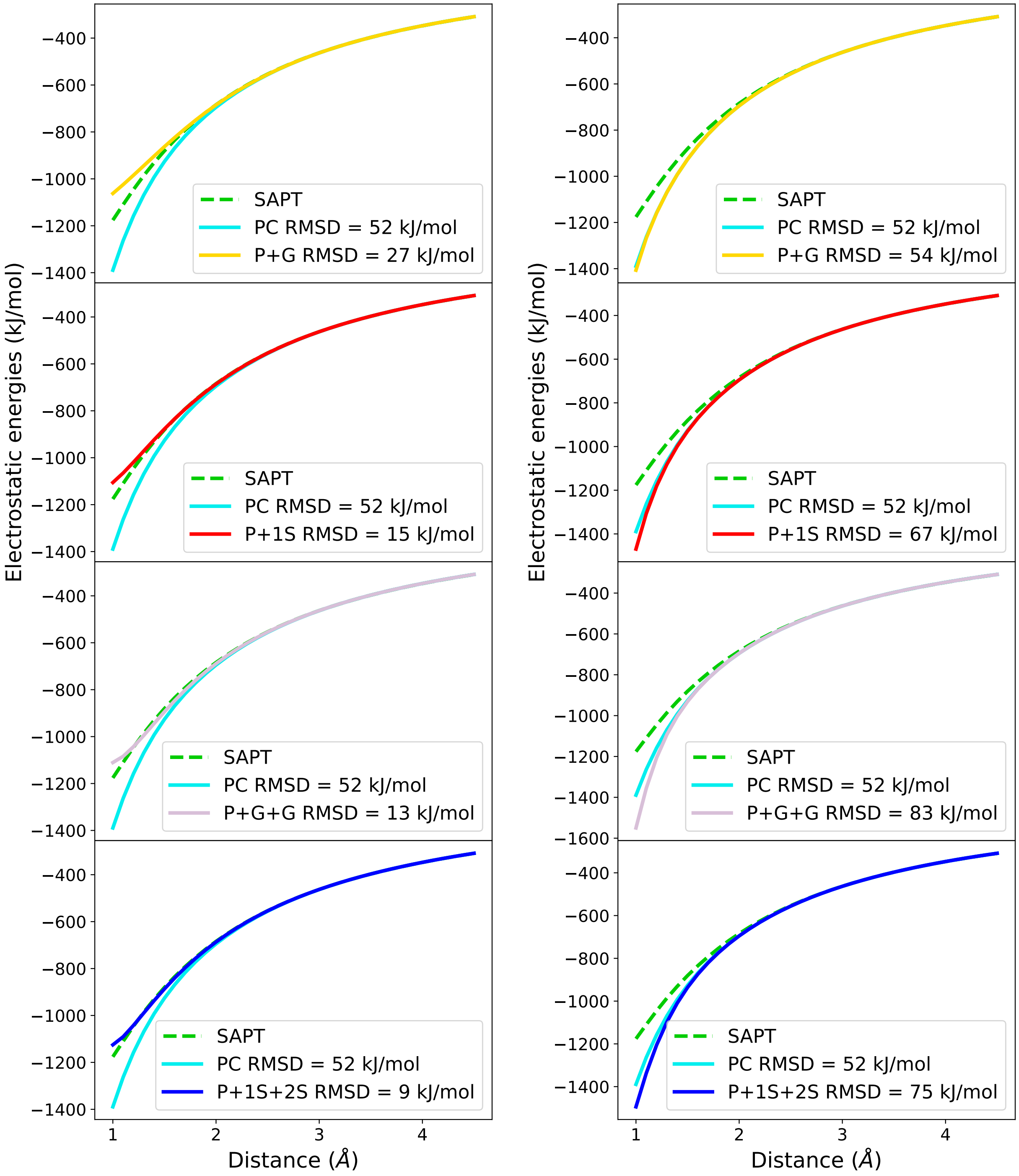

Figure S2: Electrostatic energies from SAPT0 with the aug-cc-pVTZ basis set and **four different charge models** based on fitting the ESP from 2.0 to 4.5 Å (left) and 0.0 to 4.5 Å (right) for LiF. Note that units on the y-axis may differ between plots. RMSD is computed for the points plotted, but it should be noted the range of points does not correspond to the range used for fitting, for those numbers please see Tables S1 and S2.

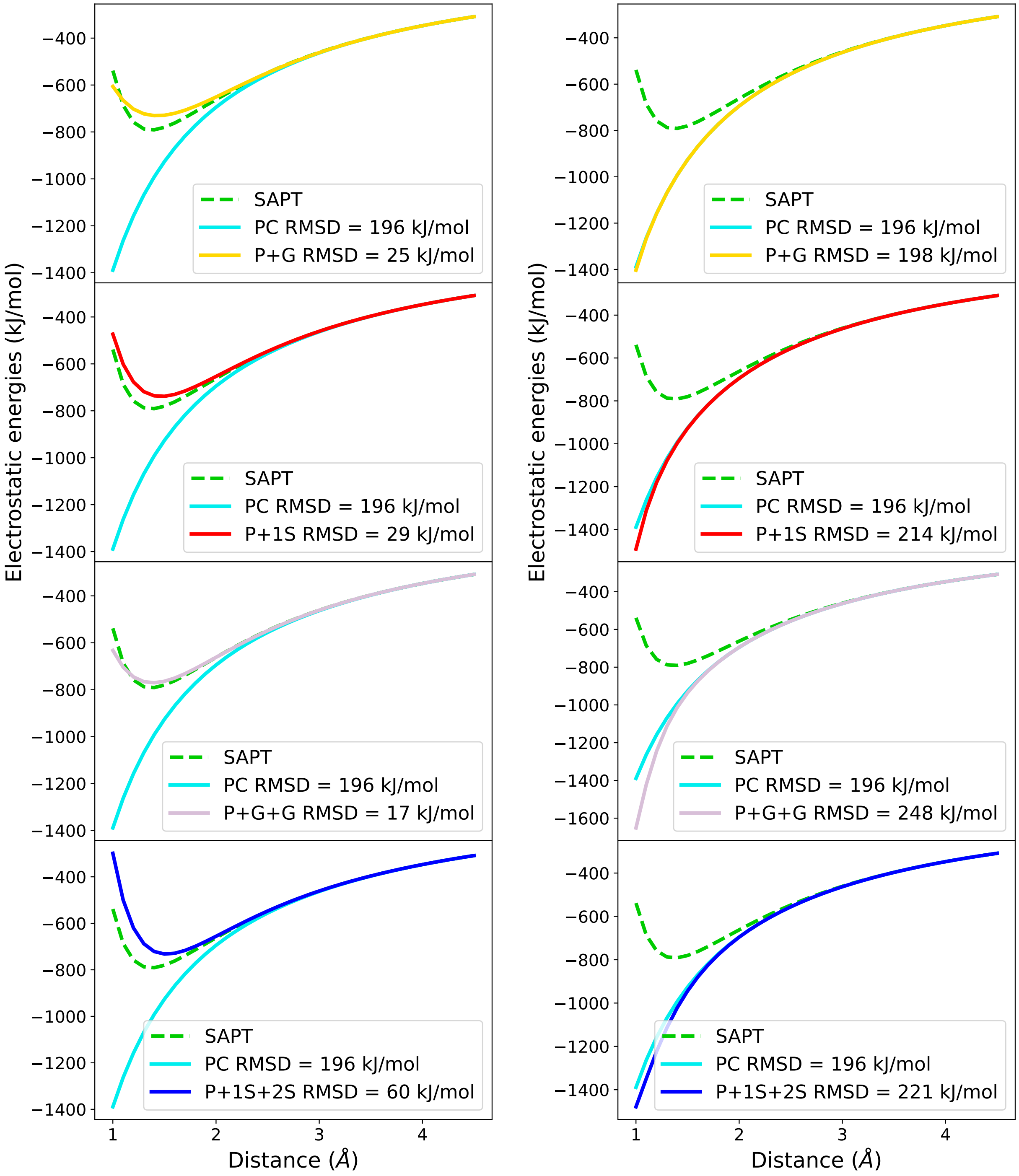

Figure S3: Electrostatic energies from SAPT0 with the aug-cc-pVTZ basis set and **four different charge models** based on fitting the ESP from 2.0 to 4.5 Å (left) and 0.0 to 4.5 Å (right) for LiCl. Note that units on the y-axis may differ between plots. RMSD is computed for the points plotted, but it should be noted the range of points does not correspond to the range used for fitting, for those numbers please see Tables S1 and S2.

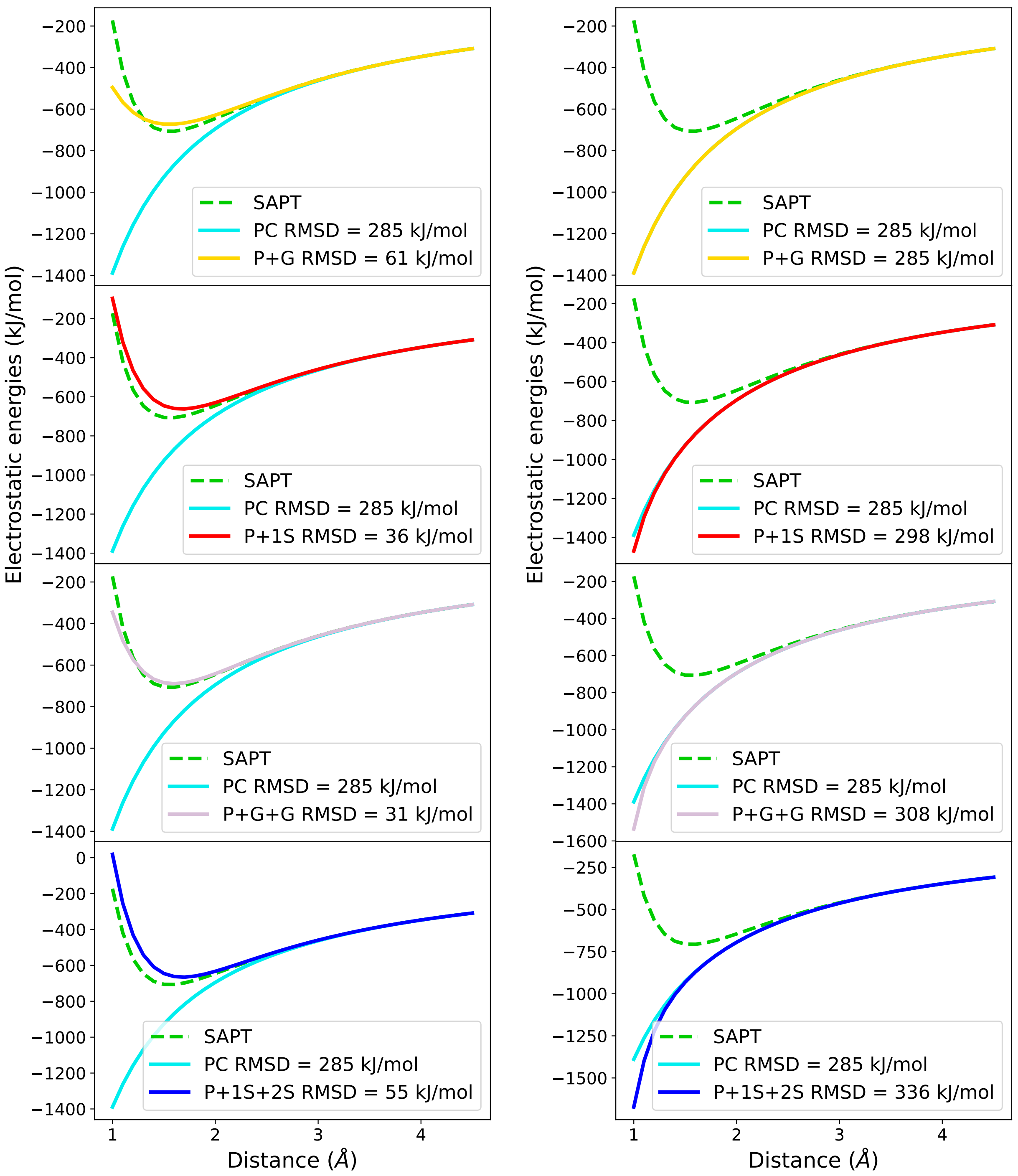

Figure S4: Electrostatic energies from SAPT0 with the aug-cc-pVTZ basis set and **four different charge models** based on fitting the ESP from 2.0 to 4.5 Å (left) and 0.0 to 4.5 Å (right) for LiBr. Note that units on the y-axis may differ between plots. RMSD is computed for the points plotted, but it should be noted the range of points does not correspond to the range used for fitting, for those numbers please see Tables S1 and S2.

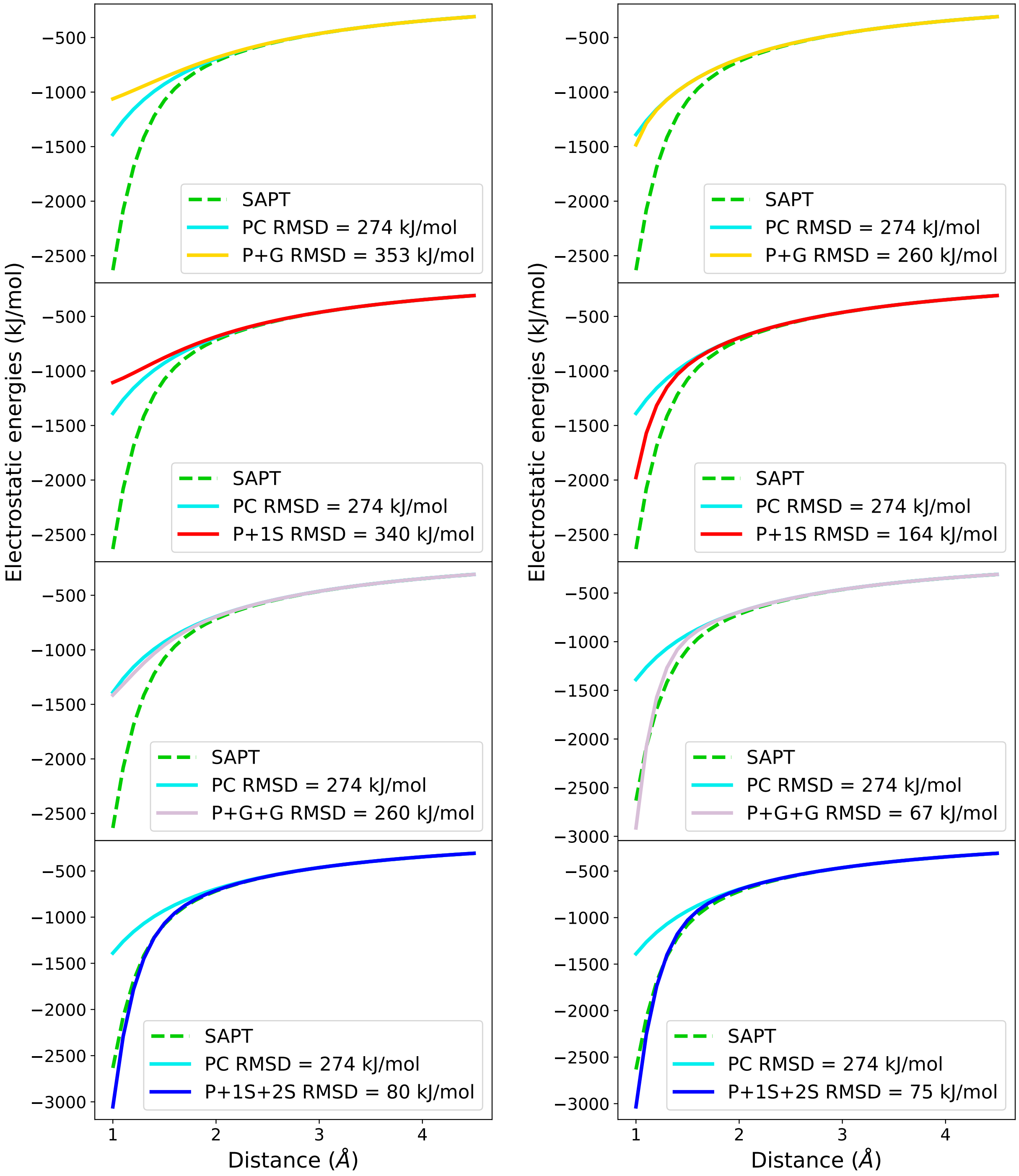

Figure S5: Electrostatic energies from SAPT0 with the aug-cc-pVTZ basis set and **four different charge models** based on fitting the ESP from 2.0 to 4.5 Å (left) and 0.0 to 4.5 Å (right) for NaF. Note that units on the y-axis may differ between plots. RMSD is computed for the points plotted, but it should be noted the range of points does not correspond to the range used for fitting, for those numbers please see Tables S1 and S2.

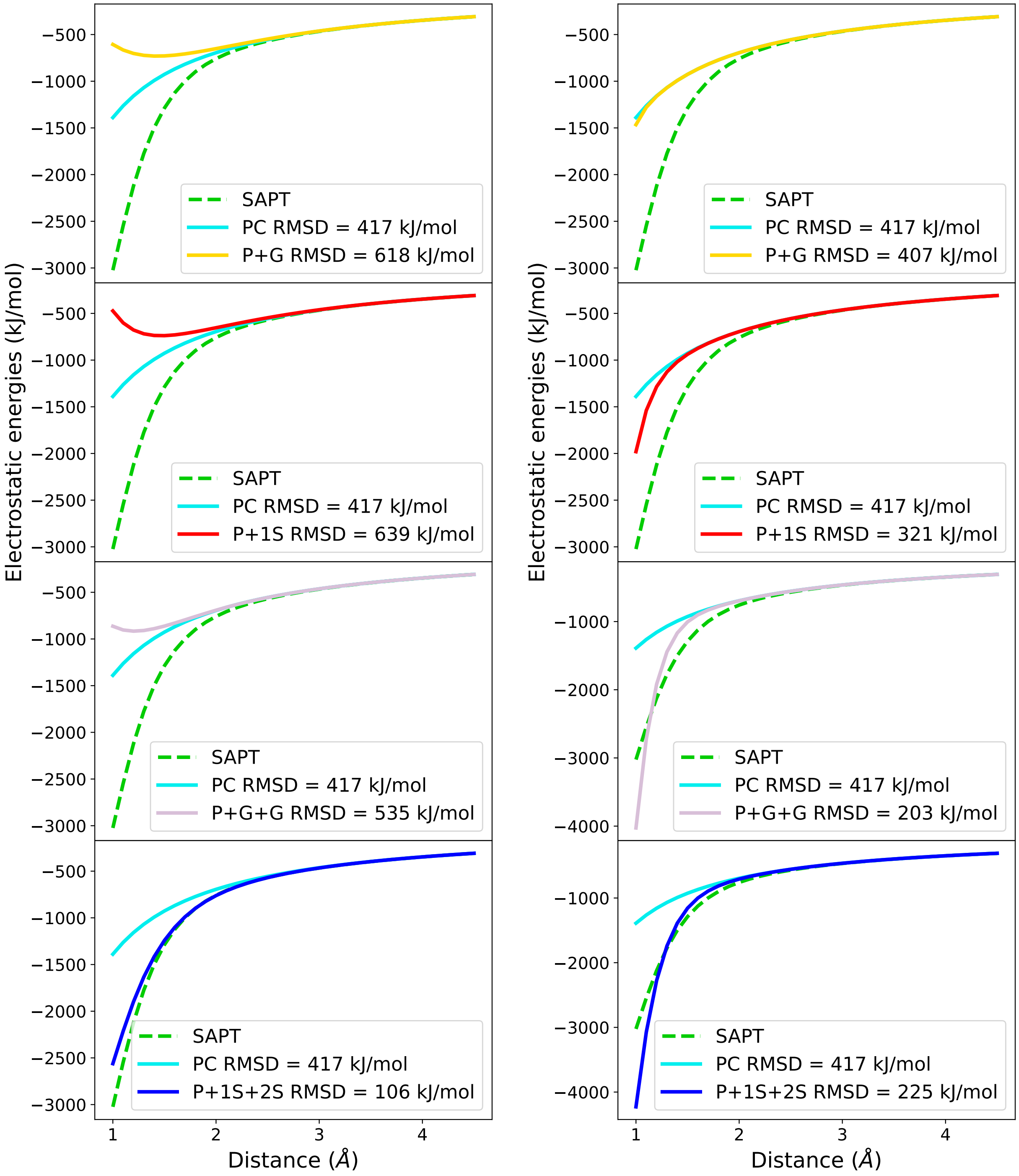

Figure S6: Electrostatic energies from SAPT0 with the aug-cc-pVTZ basis set and **four different charge models** based on fitting the ESP from 2.0 to 4.5 Å (left) and 0.0 to 4.5 Å (right) for NaCl. Note that units on the y-axis may differ between plots. RMSD is computed for the points plotted, but it should be noted the range of points does not correspond to the range used for fitting, for those numbers please see Tables S1 and S2.

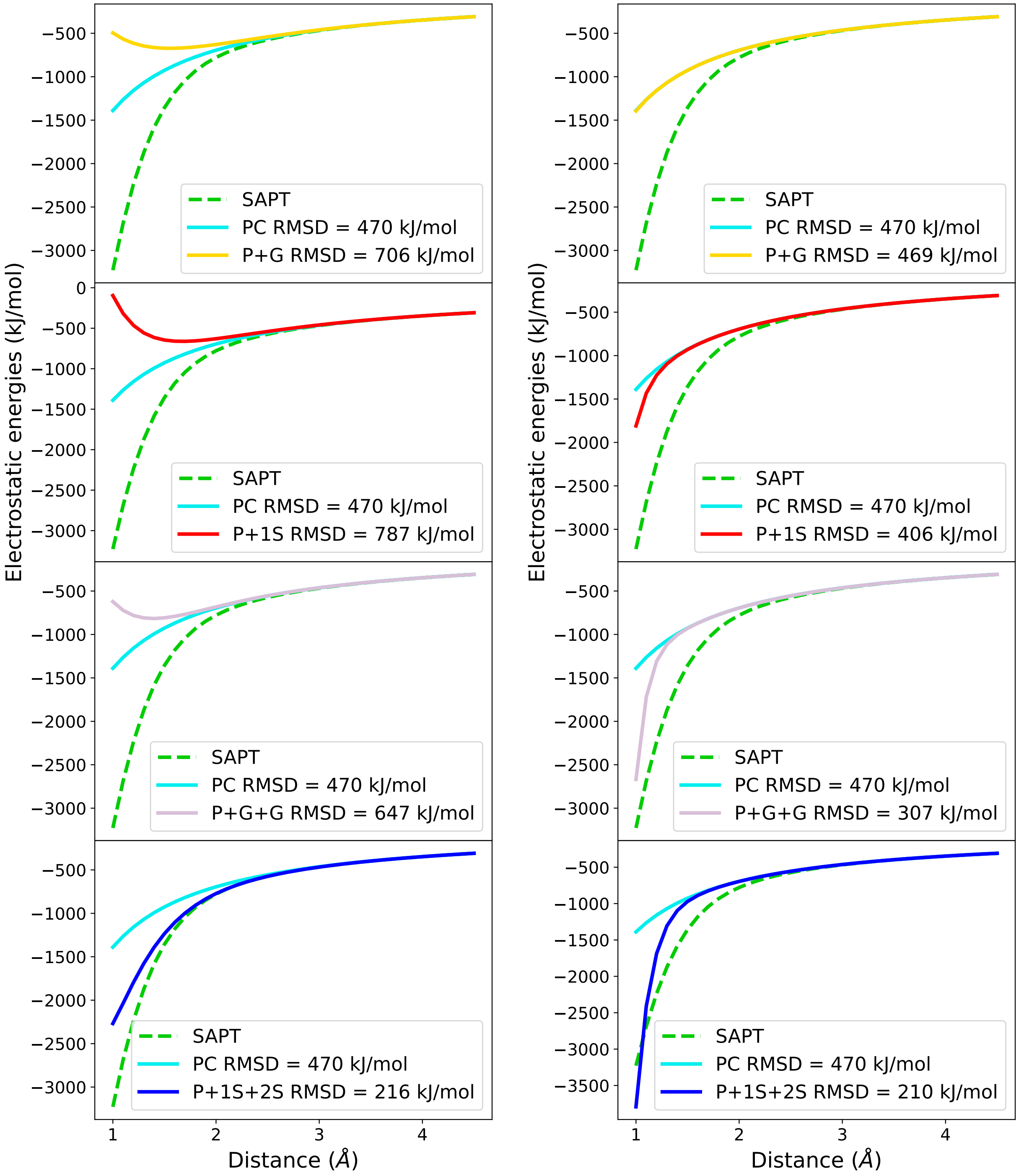

Figure S7: Electrostatic energies from SAPT0 with the aug-cc-pVTZ basis set and **four different charge models** based on fitting the ESP from 2.0 to 4.5 Å (left) and 0.0 to 4.5 Å (right) for NaBr. Note that units on the y-axis may differ between plots. RMSD is computed for the points plotted, but it should be noted the range of points does not correspond to the range used for fitting, for those numbers please see Tables S1 and S2.

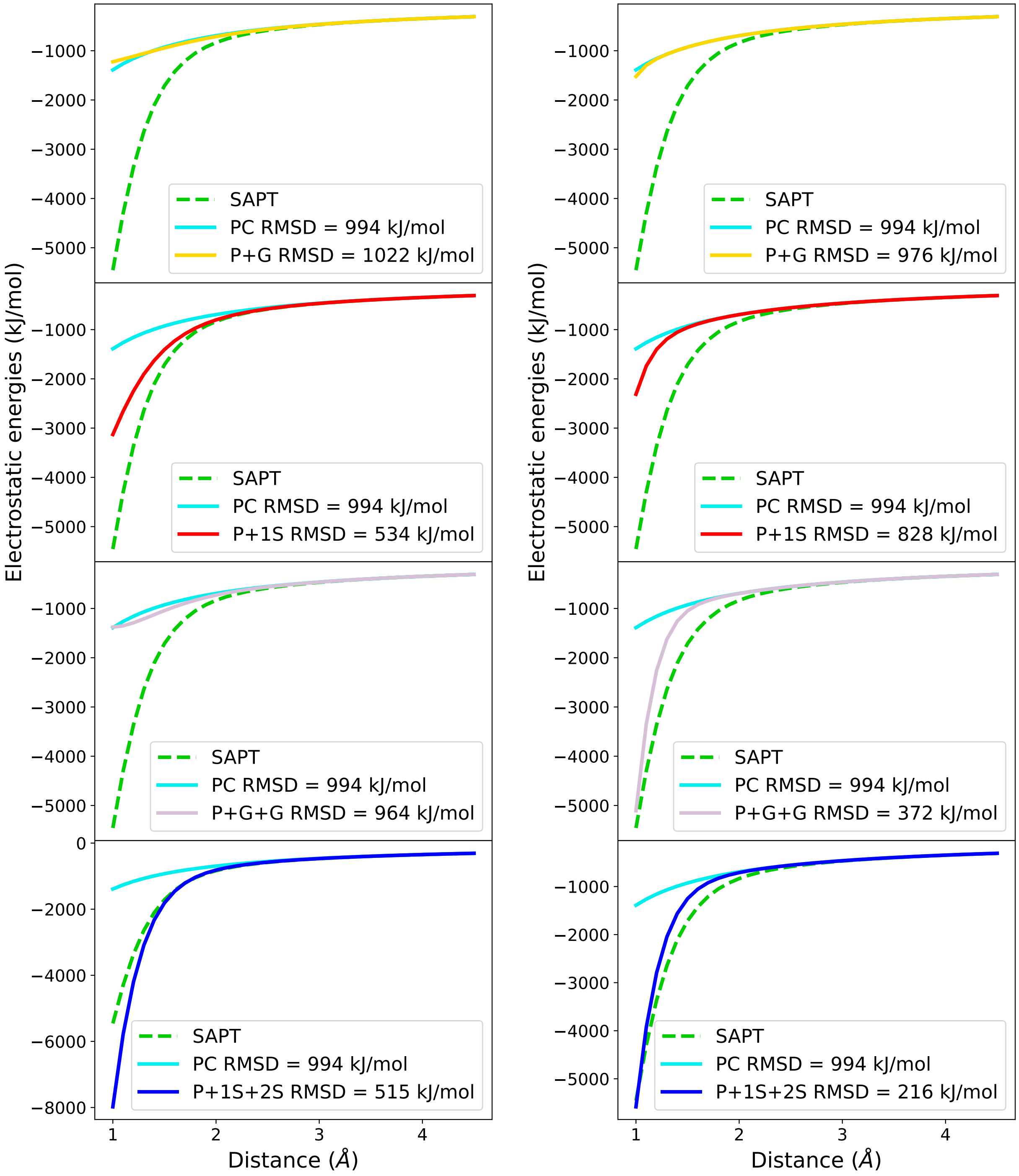

Figure S8: Electrostatic energies from SAPT0 with the aug-cc-pVTZ basis set and **four different charge models** based on fitting the ESP from 2.0 to 4.5 Å (left) and 0.0 to 4.5 Å (right) for KF. Note that units on the y-axis may differ between plots. RMSD is computed for the points plotted, but it should be noted the range of points does not correspond to the range used for fitting, for those numbers please see Tables S1 and S2.

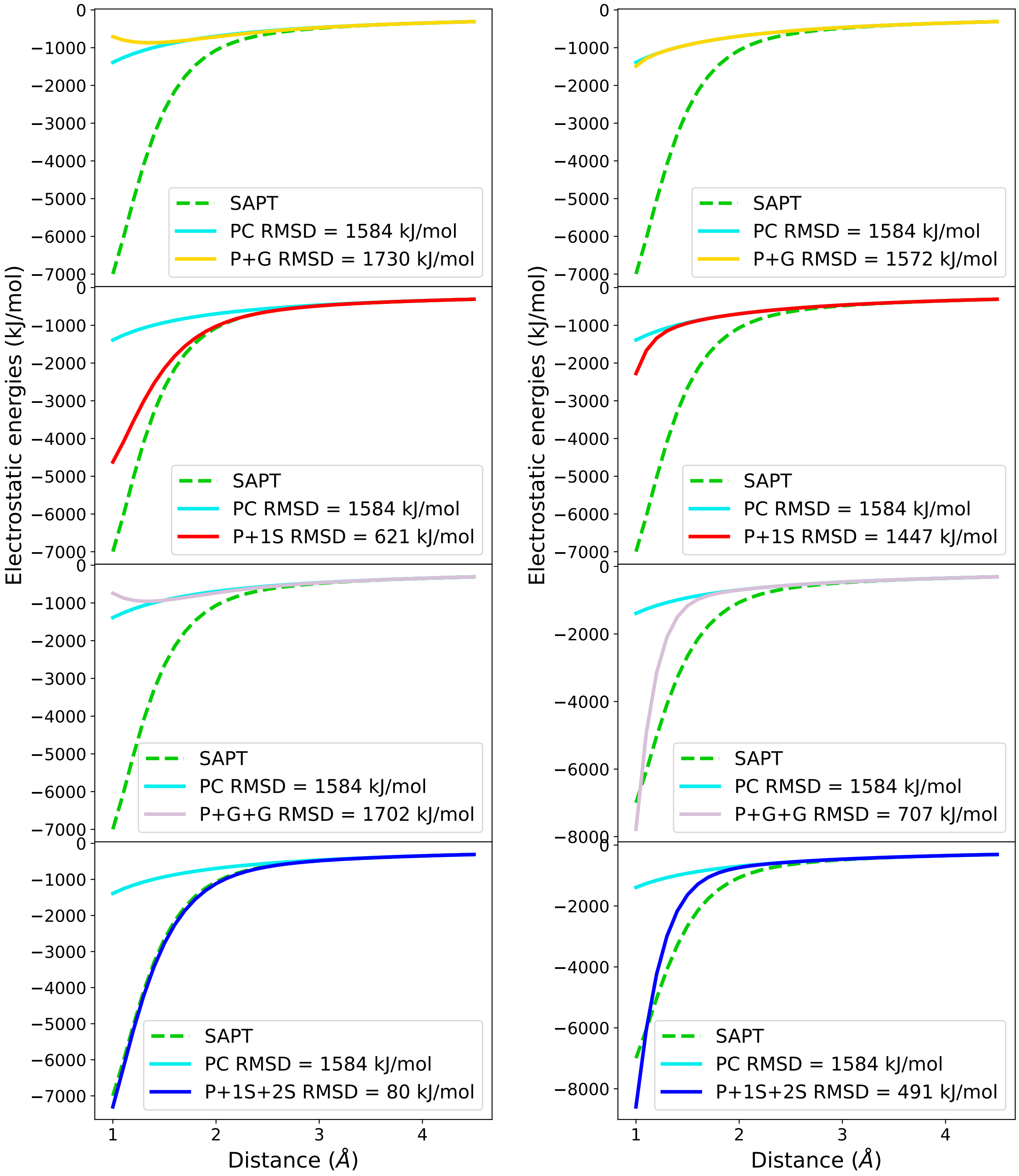

Figure S9: Electrostatic energies from SAPT0 with the aug-cc-pVTZ basis set and **four different charge models** based on fitting the ESP from 2.0 to 4.5 Å (left) and 0.0 to 4.5 Å (right) for KCl. Note that units on the y-axis may differ between plots. RMSD is computed for the points plotted, but it should be noted the range of points does not correspond to the range used for fitting, for those numbers please see Tables S1 and S2.

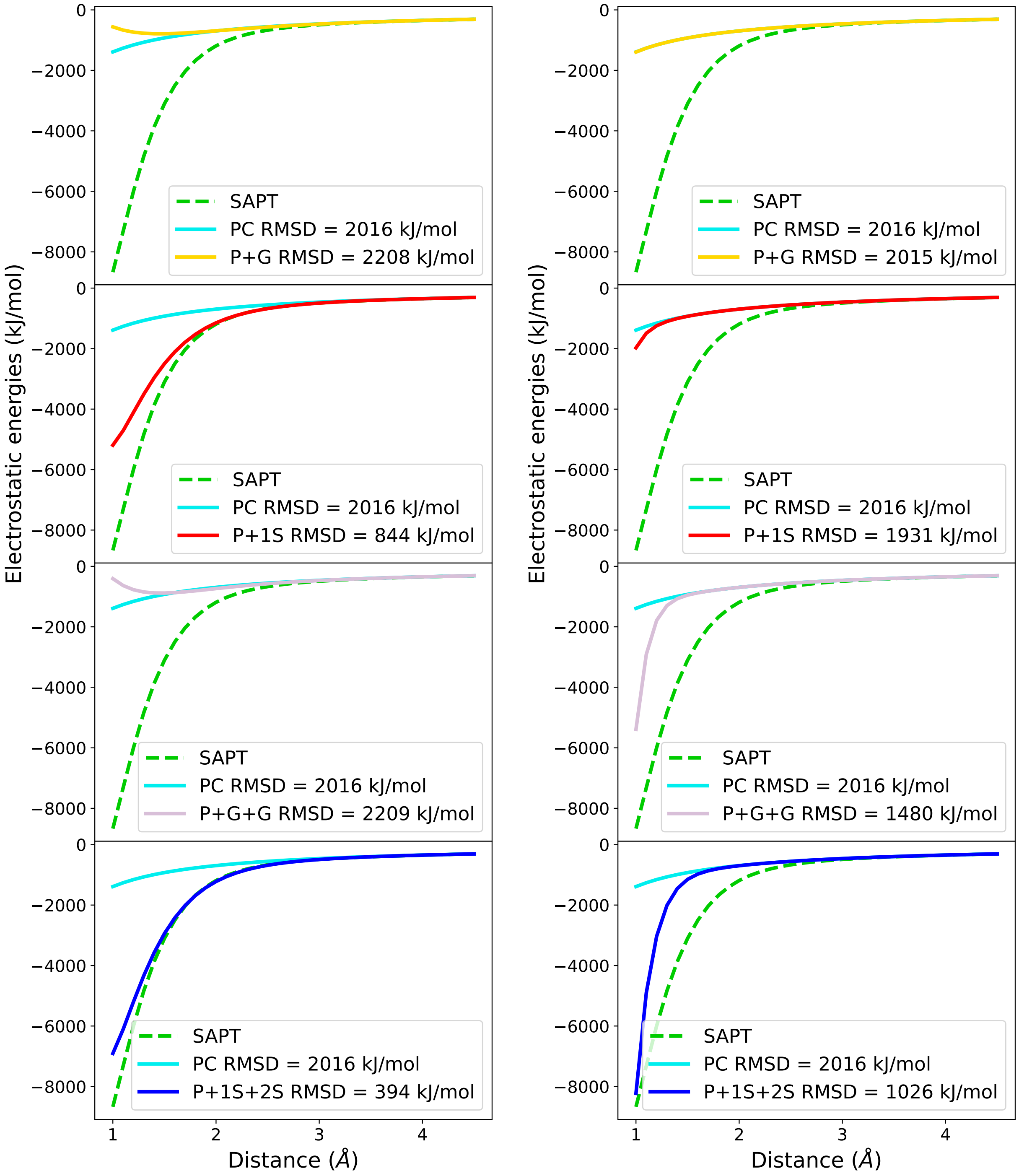

Figure S10: Electrostatic energies from SAPT0 with the aug-cc-pVTZ basis set and **four different charge models** based on fitting the ESP from 2.0 to 4.5 Å (left) and 0.0 to 4.5 Å (right) for KBr. Note that units on the y-axis may differ between plots. RMSD is computed for the points plotted, but it should be noted the range of points does not correspond to the range used for fitting, for those numbers please see Tables S1 and S2.

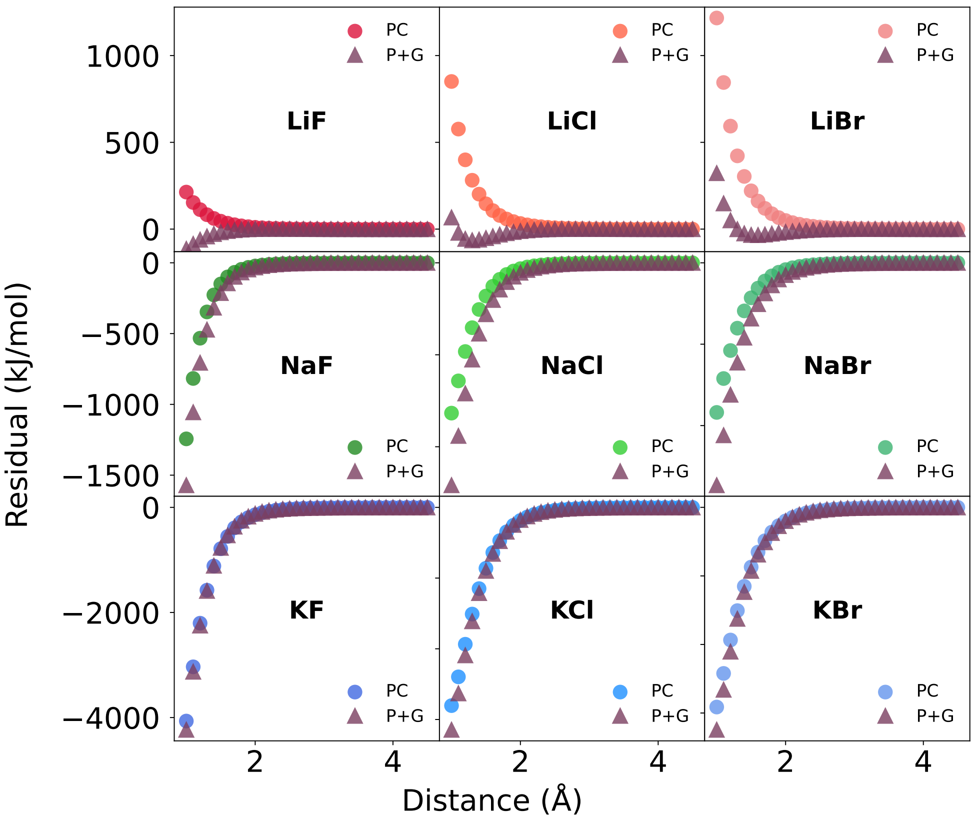

Figure S11: Residual electrostatic interactions computed using point charges and using the P+G model fitted to the ESP in the 2.0–4.5 Å range with respect to SAPT0 for nine ion-pairs.

Table S18: ESP parameters for ions and charge models (CM), charge on core q_c and shells q_s i and ii, respectively, distribution widths ζ in 1/Å. Charge models include a positive point charge with either one Gaussian (PC+G) or 1S Slater distributed charge (PC+1S), and a point charge with two Gaussian charges (PC+G+G), or a point charge with a 1S and a 2S Slater charge (PC+1S+2S). Root mean square error (kJ/mol e) after fitting from 0.0 to 4.5 Å.

Ion	CM	q_c	$q_s i$	$q_s ii$	$\zeta_s i$	$\zeta_s ii$	RMSE
F-	PC+G	8.745	-9.745	-	2.938	-	2187.14797
F-	PC+1S	8.794	-9.794	-	3.597	-	1749.34125
F-	PC+G+G	8.994	-1.894	-8.099	14.971	1.937	267.98172
F-	PC+1S+2S	8.998	-2.320	-7.678	15.187	4.029	136.76619
Cl-	PC+G	16.000	-17.000	-	3.240	-	5986.64891
Cl-	PC+1S	16.493	-17.493	-	4.393	-	4616.29399
Cl-	PC+G+G	16.968	-4.776	-13.193	15.738	1.884	1550.13258
Cl-	PC+1S+2S	16.977	-5.946	-12.032	15.605	3.704	1163.53933
Br-	PC+G	33.655	-34.655	-	4.780	-	13003.52706
Br-	PC+1S	33.933	-34.933	-	5.897	-	11063.17921
Br-	PC+G+G	35.167	-28.618	-7.549	2.898	26.565	3583.74071
Br-	PC+1S+2S	35.141	-9.389	-26.751	26.034	5.845	2828.25344
Li+	PC+G	2.988	-1.988	-	4.156	-	135.75859
Li+	PC+1S	2.999	-1.999	-	5.028	-	16.82031
Li+	PC+G+G	2.999	-0.584	-1.415	7.683	3.127	15.43725
Li+	PC+1S+2S	3.000	-1.627	-0.373	5.618	5.451	0.24157
Na+	PC+G	10.739	-9.739	-	4.075	-	2212.63447
Na+	PC+1S	10.801	-9.801	-	4.994	-	1700.13563
Na+	PC+G+G	11.000	-1.756	-8.244	19.532	2.859	222.93460
Na+	PC+1S+2S	11.000	-2.204	-7.796	19.498	5.963	69.58918
K+	PC+G	18.383	-17.383	-	4.306	-	5591.87720
K+	PC+1S	18.504	-17.504	-	5.287	-	4626.79400
K+	PC+G+G	19.000	-13.634	-4.367	2.468	19.400	1469.06094
K+	PC+1S+2S	19.001	-5.527	-12.474	18.973	4.903	1075.28380

Table S19: ESP parameters for ions and charge models (CM), charge on core q_c and shells q_s i and ii, respectively, distribution widths ζ in 1/Å. Charge models include a positive point charge with either one Gaussian (PC+G) or 1S Slater distributed charge (PC+1S), and a point charge with two Gaussian charges (PC+G+G), or a point charge with a 1S and a 2S Slater charge (PC+1S+2S). Root mean square error (kJ/mol e) after fitting from 2.0 to 4.5 Å.

Ion	CM	q_c	$q_s i$	$q_s ii$	$\zeta_s i$	$\zeta_s ii$	RMSE
F-	PC+G	0.100	-1.100	-	0.879	-	0.35831
F-	PC+1S	1.000	-2.000	-	1.623	-	0.08342
F-	PC+G+G	1.281	-2.097	-0.184	1.177	0.671	0.00100
F-	PC+1S+2S	10.000	-10.531	-0.469	2.978	1.810	0.01139
Cl-	PC+G	1.000	-2.000	-	0.761	-	0.84782
Cl-	PC+1S	3.786	-4.786	-	1.436	-	0.11662
Cl-	PC+G+G	1.408	-0.171	-2.237	0.529	0.848	0.00874
Cl-	PC+1S+2S	7.037	-7.933	-0.104	1.642	1.225	0.00954
Br-	PC+G	1.000	-2.000	-	0.701	-	0.73492
Br-	PC+1S	5.156	-6.156	-	1.378	-	0.12048
Br-	PC+G+G	2.065	-2.780	-0.285	0.823	0.528	0.02851
Br-	PC+1S+2S	7.234	-8.145	-0.089	1.496	1.155	0.00619
Li+	PC+G	1.000	-0.000	-	0.400	-	0.00003
Li+	PC+1S	1.000	-0.000	-	0.687	-	0.00002
Li+	PC+G+G	2.560	-1.475	-0.085	3.498	3.470	0.00011
Li+	PC+1S+2S	2.473	-1.463	-0.010	9.735	3.690	0.00006
Na+	PC+G	1.000	-0.000	-	0.621	-	0.00053
Na+	PC+1S	1.001	-0.001	-	1.231	-	0.00048
Na+	PC+G+G	8.723	-7.723	-0.000	3.692	0.621	0.00053
Na+	PC+1S+2S	11.000	-10.000	-0.000	3.985	0.753	0.00006
K+	PC+G	2.034	-1.034	-	1.130	-	0.00223
K+	PC+1S	20.000	-19.000	-	2.858	-	0.00894
K+	PC+G+G	2.342	-1.342	-0.000	1.159	0.568	0.00017
K+	PC+1S+2S	20.000	-19.000	-0.000	2.858	1.062	0.00894

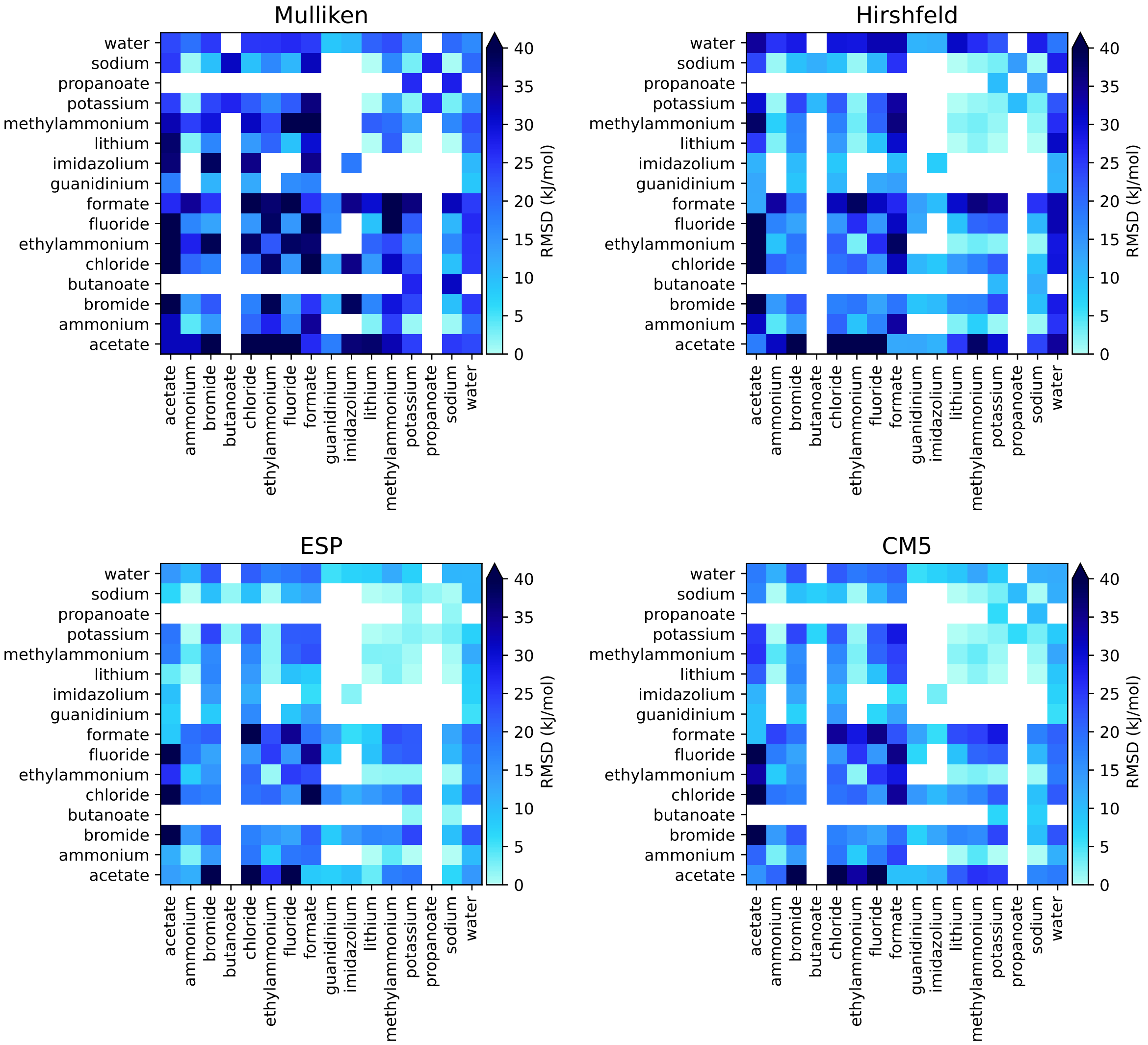

Figure S12: Heatmaps for root mean square deviation of electrostatic energies from SAPT results for existing models.

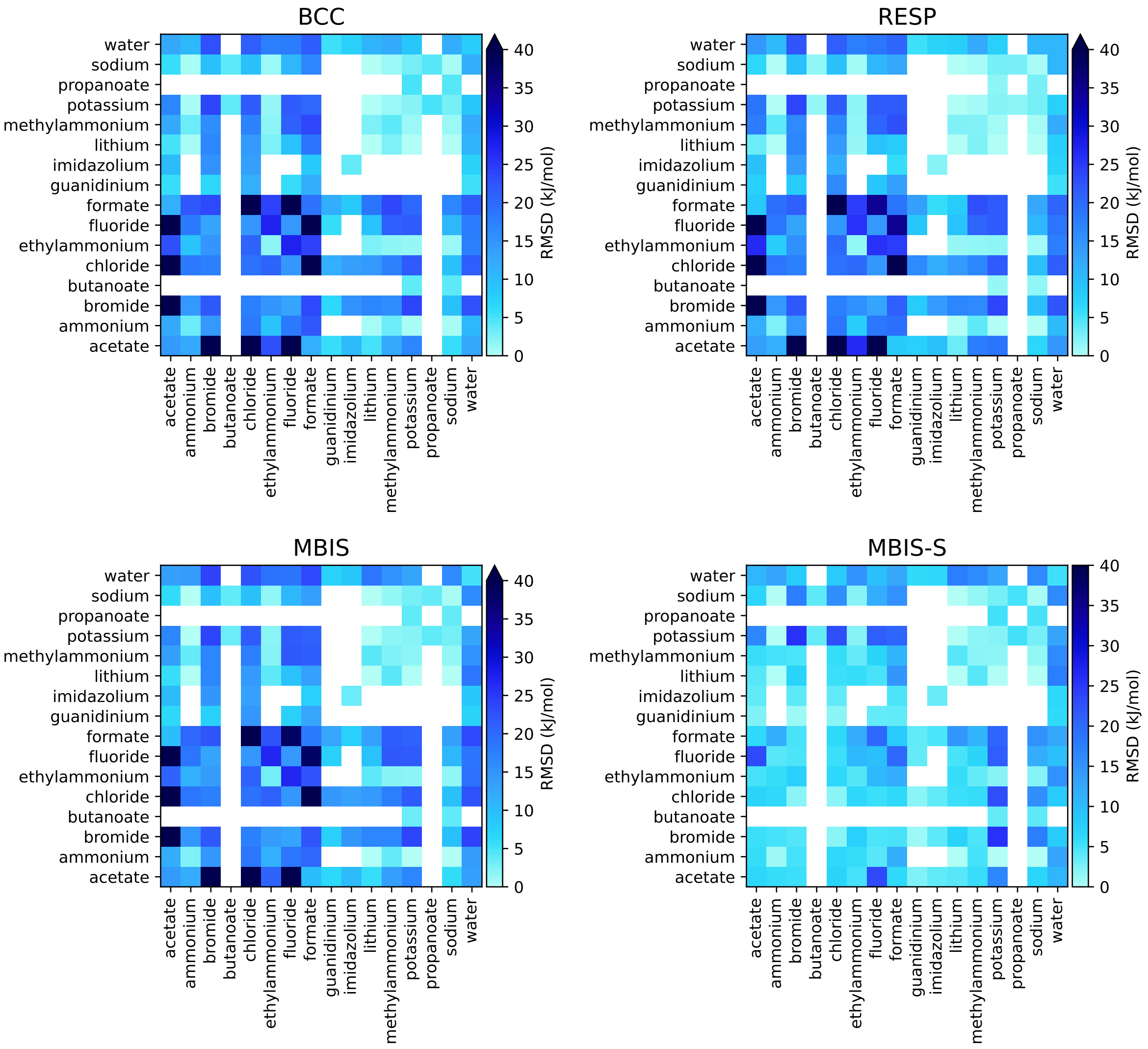

Figure S13: Heatmaps for root mean square deviation of electrostatic energies from SAPT results for existing models and ACT models.

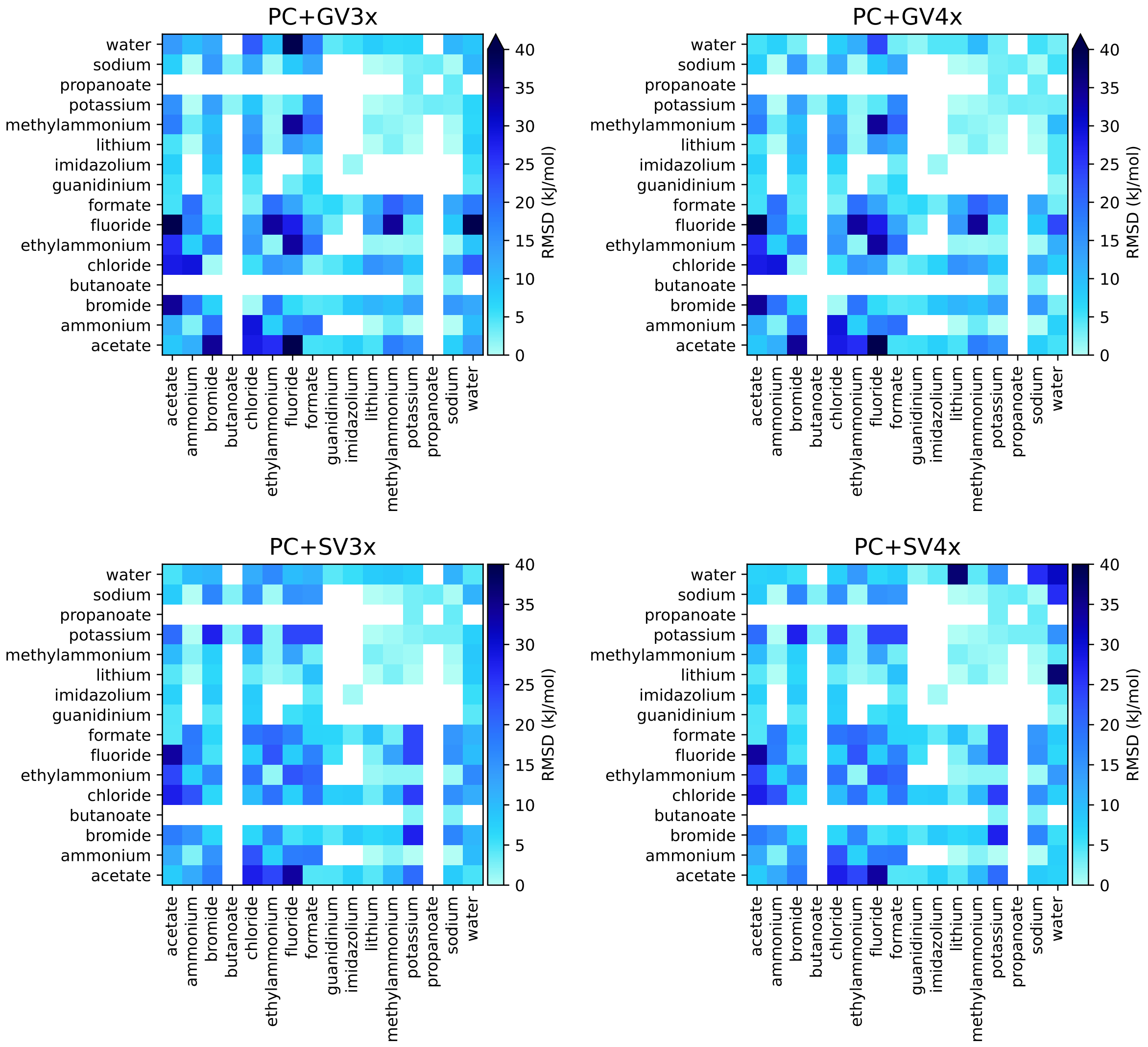

Figure S14: Heatmaps for root mean square deviation of electrostatic energies from SAPT results for ACT models derived from ESP fitting of charges and ζ.

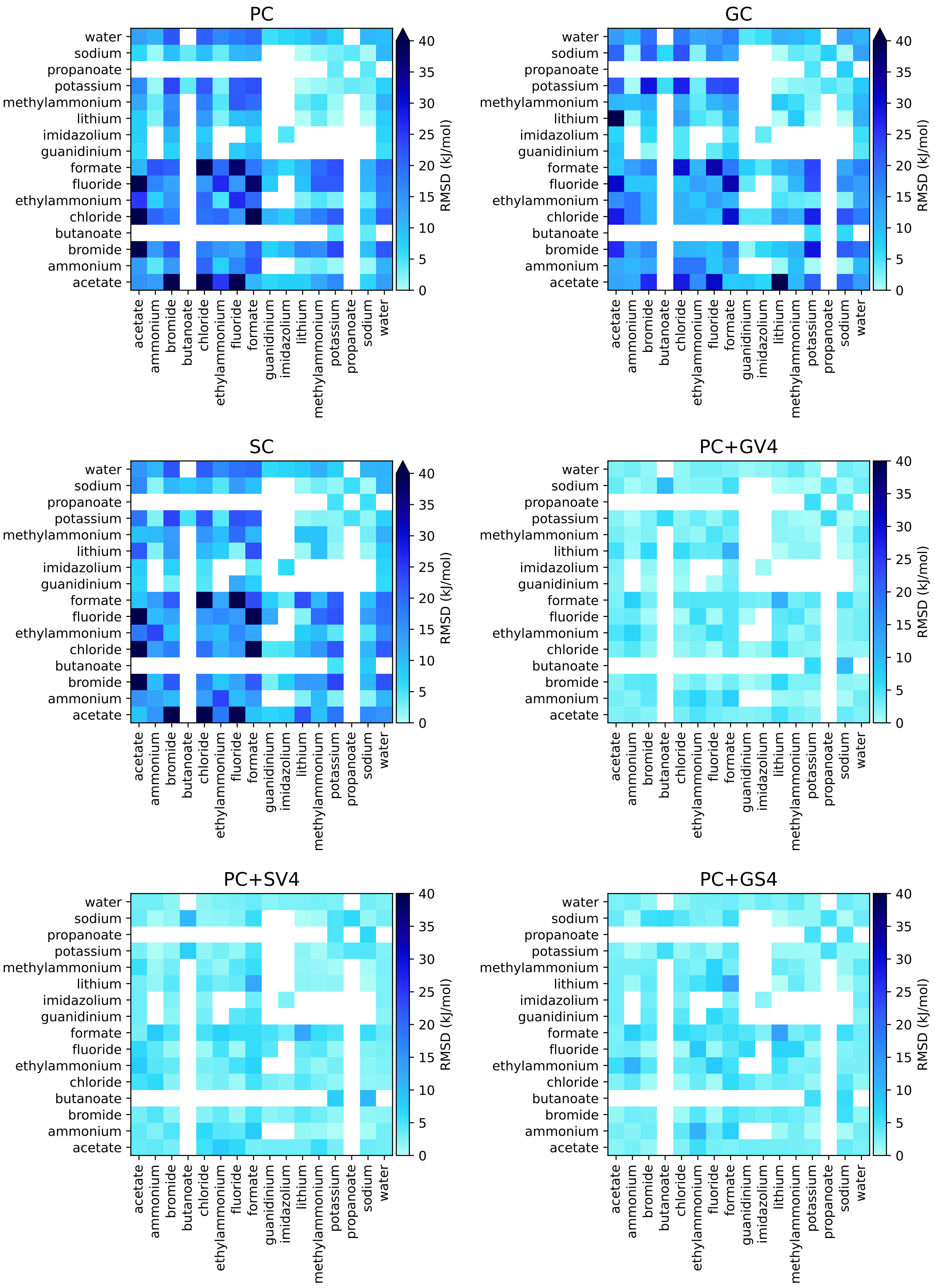

Figure S15: Heatmaps for root mean square deviation of electrostatic energies from SAPT results for ACT models derived by training on dimer energies.

S5 Interaction energies from ESP-based models

Table S20: Electrostatic energies at the experimental minimum energy distance[S1] based on the SAPT0[S2] level of theory. Point charge (PC) energy follows from Coulomb's law. ESP indicates model consisting of a point charge, combined with A: a Gaussian charge, B: a 1S Slater charge, C: two Gaussian charges or D: a 1S and a 2S Slater charge fitted to the Hartree-Fock electrostatic potential from 0.0 to 4.5 Å.

Ion pair	r_{min}	E_{elec} (kJ/mol)					
	(Å)	SAPT0	PC	ESP A	ESP B	ESP C	ESP D
LiF	1.564	-867.1	-888.3	-888.3	-890.4	-890.8	-895.0
LiCl	2.021	-661.4	-687.5	-687.5	-687.5	-687.5	-688.5
LiBr	2.17	-612.4	-640.3	-640.3	-640.3	-640.3	-640.3
NaF	1.926	-749.0	-721.4	-721.4	-722.4	-722.0	-728.2
NaCl	2.361	-607.7	-588.5	-588.5	-588.5	-588.5	-589.8
NaBr	2.502	-572.4	-555.3	-555.3	-555.3	-555.3	-555.3
KF	2.171	-704.6	-640.0	-640.0	-640.2	-640.2	-645.4
KCl	2.667	-566.1	-520.9	-520.9	-520.9	-520.9	-521.7
KBr	2.821	-535.3	-492.5	-492.5	-492.5	-492.5	-492.5
RMSD			35.6	35.6	35.6	35.6	34.4
MSE			15.7	15.7	15.3	15.3	13.3

Table S21: Electrostatic energies at the experimental minimum energy distance[S1] based on the SAPT0[S2] level of theory. Point charge (PC) energy follows from Coulomb's law. ESP indicates model consisting of a point charge, combined with A: a Gaussian charge, B: a 1S Slater charge, C: two Gaussian charges or D: a 1S and a 2S Slater charge fitted to the Hartree-Fock electrostatic potential from 2.0 to 4.5 Å.

Ion pair	r_{min}	E_{elec} (kJ/mol)					
	(Å)	SAPT0	PC	ESP A	ESP B	ESP C	ESP D
LiF	1.564	-867.1	-888.3	-837.6	-849.1	-863.2	-856.4
LiCl	2.021	-661.4	-687.5	-646.7	-648.8	-656.9	-651.0
LiBr	2.17	-612.4	-640.3	-599.9	-600.5	-608.3	-602.8
NaF	1.926	-749.0	-721.4	-708.1	-709.9	-725.6	-742.4
NaCl	2.361	-607.7	-588.5	-575.5	-574.5	-588.7	-609.7
NaBr	2.502	-572.4	-555.3	-540.7	-539.9	-553.8	-573.5
KF	2.171	-704.6	-640.0	-654.1	-703.7	-659.8	-705.0
KCl	2.667	-566.1	-520.9	-530.8	-573.3	-531.3	-576.3
KBr	2.821	-535.3	-492.5	-499.9	-543.9	-501.7	-544.5
RMSD			35.6	33.3	22.2	25.1	7.8
MSE			15.7	31.4	14.7	20.7	1.6

Table S22: RMSD (kJ/mol e) with respect to the electrostatic potential computed at the MP2/aug-cc-pvtz level of theory (see Methods) for the side chain analogs in Table 1 and water. For description of the different models and training, see Methods.

Model	Train	water	acetate	methylammonium	ammonium	ethylammonium	formate	propanoate	butanoate	guanidinium	imidazolium	Average
Mulliken	Other	21.7	50.3	38.5	7.0	35.8	43.4	56.1	57.5	34.5	59.7	40.5
Hirshfeld	Other	31.3	27.5	7.6	7.6	7.1	26.7	28.0	27.3	27.4	22.4	21.3
ESP	Other	10.7	6.0	4.4	1.0	3.9	7.7	6.0	5.2	2.6	4.4	5.2
CM5	Other	11.5	22.1	5.0	1.9	4.6	20.1	23.4	23.1	6.9	8.0	12.7
BCC	Other	13.4	10.1	5.9	1.4	5.7	15.1	12.4	9.8	11.8	7.6	9.3
RESP	Other	10.7	6.1	4.4	1.0	4.4	7.7	7.6	5.4	2.6	4.4	5.4
MBIS	Other	20.3	9.3	6.4	1.0	7.2	10.9	11.2	10.3	4.9	8.8	9.0
MBIS-S	Other	20.0	8.5	6.2	1.1	8.1	10.2	13.4	9.8	5.3	8.5	9.1
PC+GV3x	ESP	10.6	4.7	4.5	1.0	4.4	5.6	6.9	4.9	1.9	4.2	4.9
PC+SV3x	ESP	10.8	4.7	4.5	1.0	4.3	5.6	6.9	4.9	1.9	4.2	4.9
PC+GV4x	ESP	2.6	4.7	4.5	1.0	4.4	5.6	6.9	4.9	1.9	4.2	4.1
PC+SV4x	ESP	2.8	4.7	4.5	1.0	4.3	5.6	6.9	4.9	1.9	4.2	4.1
PC	Elec	10.9	13.7	7.5	7.2	11.6	10.5	12.5	13.4	18.6	15.6	12.1
GC	Elec	12.0	31.0	20.5	14.9	28.3	10.2	31.9	32.5	17.7	18.1	21.7
SC	Elec	10.9	26.2	15.5	14.9	30.2	20.4	33.7	31.4	34.9	18.9	23.7
PC+GV4	Elec	4.3	5.2	4.6	3.8	8.0	9.6	12.9	21.7	3.9	6.3	8.0
PC+SV4	Elec	4.8	6.9	6.0	5.3	7.5	10.2	15.8	28.1	9.6	6.9	10.1
PC+GS4	Elec	3.5	6.8	5.3	2.6	11.2	7.0	21.0	18.5	10.9	10.0	9.7

S6 Interaction energies from ACT-models

Table S23: Water-ion electrostatic energies at distances close to their energy minimum. Distance r (Å) between ions and water oxygen/hydrogen from Experiment (ref. S3), and minimized water dimer (ref. S4). Electrostatic energies are reported in kJ/mol from the SAPT2+(CCD)-δMP2 method with an aug-cc-pVTZ basis set, TIP4P-Ew[S5] with point charges representing ions, for MBIS-S,[S6] for the CHARMM drude model of water (SWM4-NDP[S7]) with ions due to Yu *et al.*,[S8] as well as point core+Gaussian vsite (PC+GV4), and point charge + Gaussian shell (PC+GS4) derived here using ACT.

Ion	r	SAPT	TIP4P-Ew	MBIS-S	CHARMM	PC+GV4	PC+GS4
water-lithium	2.04	-111.5	-131.0	-152.2	-143.3	-114.7	-113.0
water-sodium	2.39	-88.7	-99.4	-116.5	-108.7	-89.1	-89.0
water-potassium	2.78	-68.5	-75.4	-88.7	-82.5	-70.3	-70.0
water-fluoride	2.64	-146.7	-135.2	-165.6	-146.1	-140.5	-155.0
water-chloride	3.173	-76.8	-72.1	-89.4	-78.3	-81.4	-80.9
water-bromide	3.36	-60.5	-54.6	-69.4	-59.4	-63.4	-63.2
water-water	2.97	-31.6	-25.9	-35.1	-30.4	-29.9	-30.5
RMSD			10.4	22.1	15.2	3.4	3.8
MSE			-1.3	-18.9	-9.2	-0.7	-2.5

Table S24: Ion-pair electrostatic energies at distances close to their energy minimum. Distance r (Å) between ions and electrostatic energies from the SAPT2+(CCD)δMP2/aug-cc-pVTZ level of theory, for point charges (PC), for MBIS-S,[S6] for the Walz *et al.* model with a Gaussian charge distribution,[S9] and the ACT models PC+GV4 and PC+GS4 (see Methods). The RMSD and MSE were calculated with respect to the SAPT2+(CCD)δMP2 with the aug-cc-pVTZ basis set electrostatic energy. Note that this level of theory is different from Tables S18 and S19 and the results cannot be compared directly. In addition, the results in Tables S18 and S19 are from fitting models to the ESP, whereas in this table training was done on SAPT data as indicated above.

Ion	r	SAPT	PC	MBIS-S	Walz	PC+GV4	PC+GS4
lithium-fluoride	1.6	-834.4	-868.3	-851.4	-794.6	-837.4	-854.3
lithium-chloride	2.04	-652.1	-681.1	-643.4	-631.4	-667.6	-659.1
lithium-bromide	2.22	-599.1	-625.8	-589.9	-584.4	-615.6	-604.1
sodium-fluoride	1.95	-739.4	-712.5	-710.0	-683.1	-742.4	-742.0
sodium-chloride	2.4	-596.1	-578.9	-568.1	-556.6	-601.4	-593.8
sodium-bromide	2.55	-559.9	-544.8	-532.4	-524.1	-565.7	-557.4
potassium-fluoride	2.25	-679.6	-617.5	-618.6	-605.6	-684.7	-682.9
potassium-chloride	2.74	-546.9	-507.1	-505.1	-497.6	-551.5	-548.3
potassium-bromide	2.9	-516.0	-479.1	-476.2	-470.1	-520.4	-518.2
RMSD			34.6	33.2	45.0	8.6	7.5
MSE			12.0	25.4	41.8	-7.0	-4.1

Table S25: Water-ion induction energies at distances close to their energy minimum. Distance r (Å) between ions and water oxygen/hydrogen from Experiment (ref. S3), and minimized water dimer (ref. S4). Induction energies are reported in kJ/mol from the SAPT2+(CCD)-δMP2 method with an aug-cc-pVTZ basis set, for the CHARMM drude model of water (SWM4-NDP[S7]) with ions due to Yu *et al.*,[S8] as well the point charge + Gaussian shell (PC+GS4) derived here using ACT.

Ion	r	SAPT	CHARMM	PC+GS4
water-lithium	2.04	-42.6	-46.5	-37.2
water-sodium	2.39	-21.7	-23.7	-18.0
water-potassium	2.78	-14.7	-13.6	-10.9
water-fluoride	2.64	-85.1	-38.4	-46.8
water-chloride	3.173	-28.7	-13.9	-21.4
water-bromide	3.36	-20.3	-9.9	-15.0
water-water	2.97	-9.1	-2.7	-6.0
RMSD			19.1	15.2
MSE			10.5	9.5

Table S26: Electrostatic energy (kJ/mol) between alkali ions, halides or water (oxygen) and amino acid side chain analogs, formate (oxygen), acetate (oxygen), methylammonium (nitrogen), ethylammonium (nitrogen) from SAPT2+(CCD)δMP2/aug-cc-pVTZ, and charges determined using either RESP[S10] or BCC[S11] as well as two models generated using the ACT.

Ion	r	SAPT	RESP	BCC	MBIS-S	PC+GV4	PC+GS4
formate-lithium	1.848	-734.5	-770.5	-797.8	-771.9	-774.9	-772.2
formate-sodium	2.175	-674.0	-652.3	-673.5	-662.0	-686.1	-683.8
formate-potassium	2.521	-604.7	-555.0	-571.1	-565.4	-606.1	-605.2
formate-water	3.109	-136.6	-90.4	-109.0	-146.7	-132.8	-134.4
acetate-lithium	2.032	-624.7	-626.6	-642.3	-631.7	-638.4	-638.6
acetate-sodium	2.031	-646.2	-617.3	-632.6	-623.3	-650.5	-649.4
acetate-potassium	2.519	-574.5	-532.8	-544.9	-540.6	-576.6	-576.2
acetate-water	3.098	-142.2	-91.4	-108.8	-147.9	-139.5	-138.5
methylammonium-fluoride	2.551	-540.7	-513.9	-510.9	-536.0	-543.0	-547.2
methylammonium-chloride	2.853	-530.5	-501.9	-515.6	-542.0	-536.1	-535.6
methylammonium-bromide	2.915	-519.6	-488.7	-501.1	-528.0	-526.8	-523.8
methylammonium-water	2.65	-100.8	-85.5	-106.3	-143.6	-102.5	-107.9
ethylammonium-fluoride	2.41	-559.0	-523.5	-528.6	-569.9	-568.0	-572.7
ethylammonium-chloride	2.866	-518.1	-489.9	-502.1	-531.2	-520.3	-517.9
ethylammonium-bromide	2.987	-493.4	-466.5	-477.2	-503.8	-498.5	-493.6
ethylammonium-water	2.651	-99.5	-83.3	-102.9	-140.6	-99.2	-104.4
guanidinium-formate	2.594	-389.1	-356.0	-356.9	-361.3	-361.8	-354.6
guanidinium-acetate	2.403	-386.0	-346.4	-344.2	-358.3	-365.8	-359.4
guanidinium-fluoride	2.632	-395.2	-367.8	-359.2	-367.3	-371.6	-359.5
guanidinium-chloride	2.906	-367.4	-356.1	-370.9	-404.0	-398.9	-397.2
guanidinium-bromide	3.042	-368.1	-339.9	-340.4	-349.9	-351.7	-349.6
guanidinium-water	2.728	-36.8	-28.5	-30.7	-39.3	-31.4	-28.9
imidazolium-formate	2.588	-366.2	-342.7	-343.5	-355.4	-365.3	-360.0
imidazolium-acetate	3.043	-377.7	-345.1	-347.9	-366.4	-374.1	-366.6
imidazolium-chloride	2.885	-385.4	-356.8	-363.0	-374.2	-370.3	-369.3
imidazolium-bromide	2.989	-372.7	-343.7	-349.7	-361.3	-357.4	-357.4
imidazolium-water	2.462	-37.2	-31.6	-39.6	-46.0	-35.1	-35.2
RMSD			30.4	26.3	22.5	14.5	16.4
MSE			25.1	15.2	0.5	-0.1	1.6

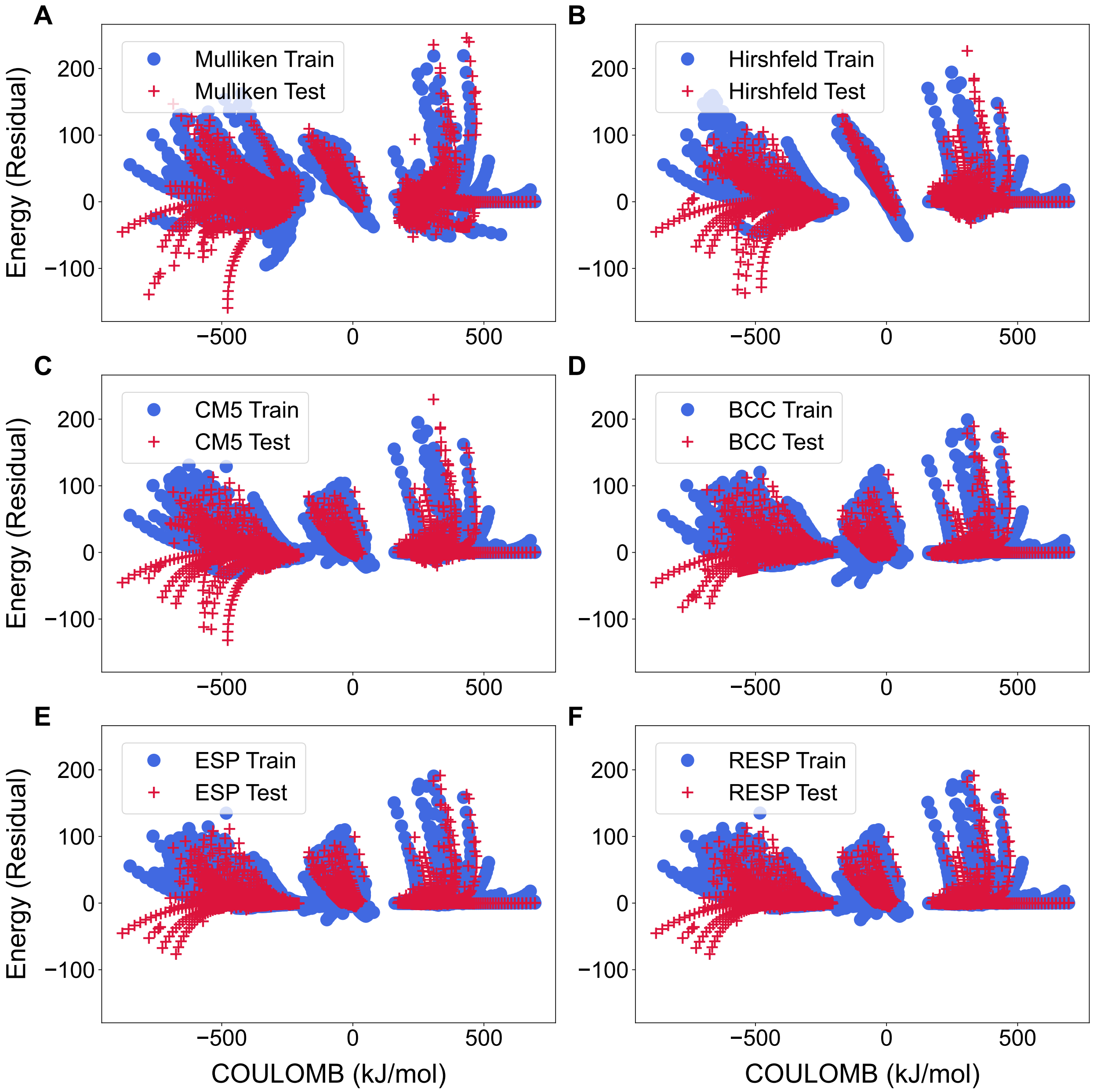

Figure S16: Residual plots for electrostatic interactions of existing models with respect to the SAPT2+(CCD)δMP2 for all compounds. A) Mulliken,[S27] B) Hirshfeld,[S28] C) CM5,[S29] D) BCC,[S11] E) ESP[S30] and F) RESP.[S10]

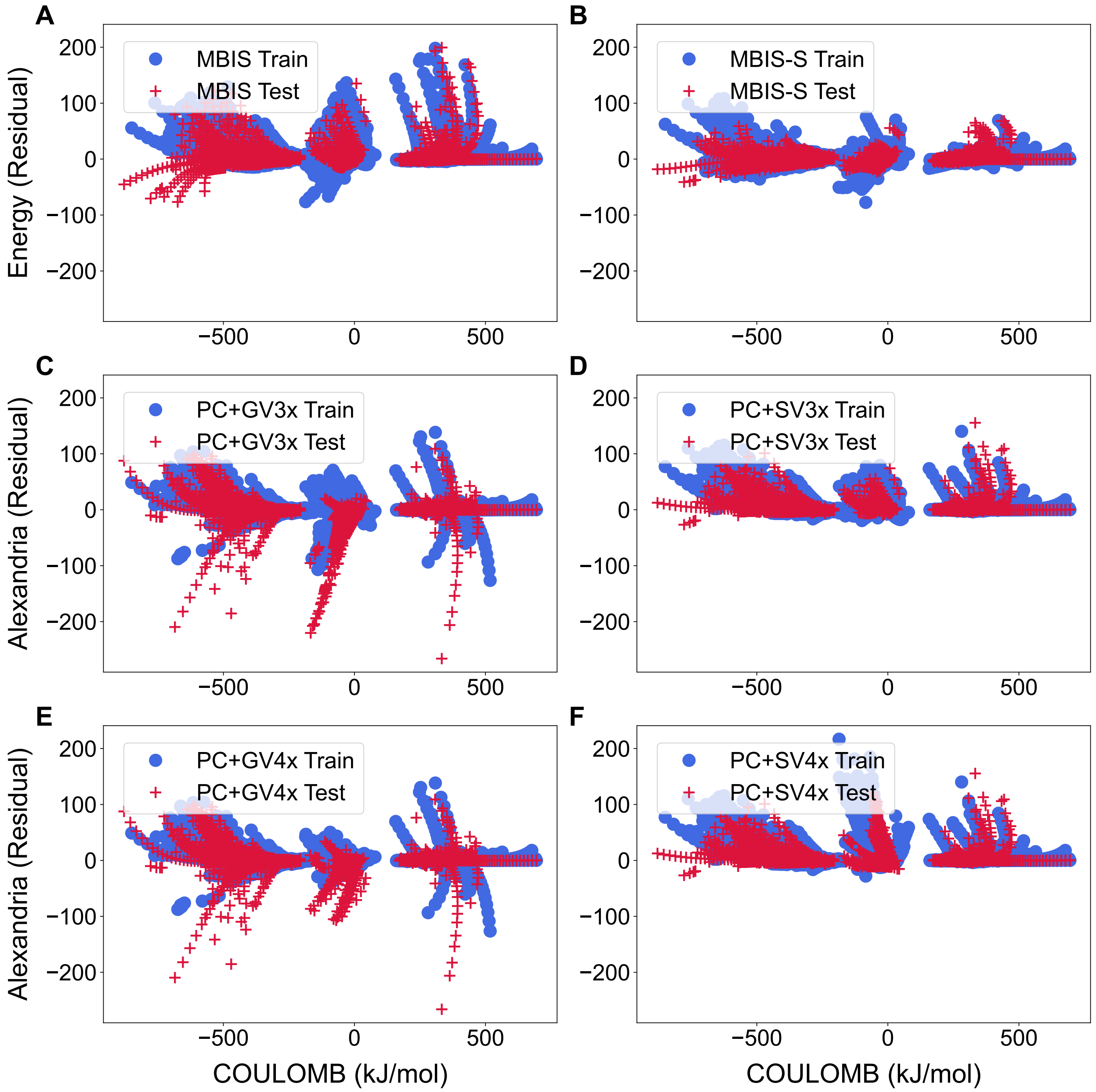

Figure S17: Residual plots for electrostatic interactions of MBIS and MBIS-S models as well as ACT-trained on ESP with respect to the SAPT2+(CCD)δMP2 for all compounds. A) MBIS,[S6] B) MBIS-S,[S6] C-F models derived in this work based on ESP fitting (see Table 2).

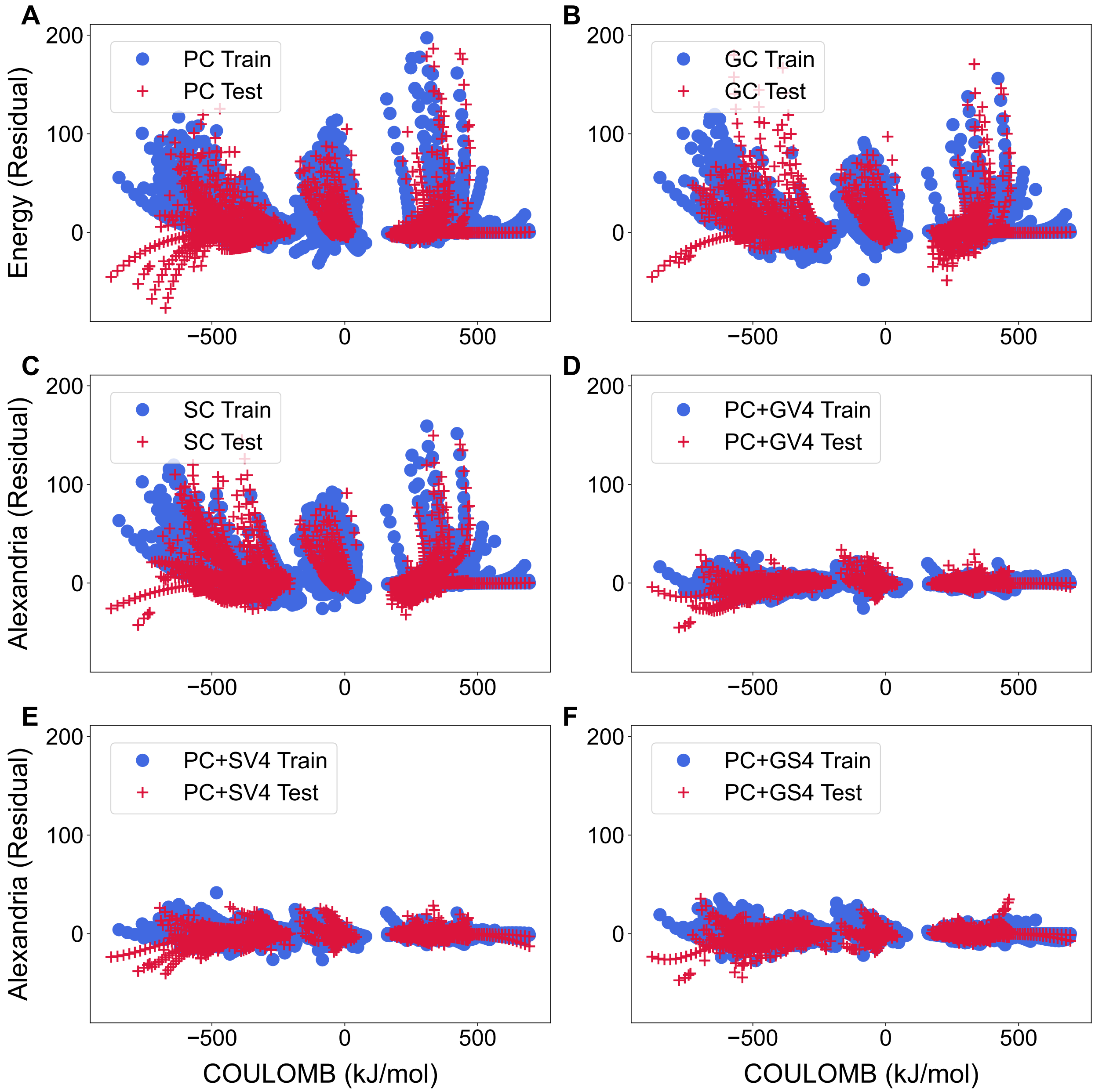

Figure S18: Residual plots for electrostatic interactions of ACT-trained models with respect to the SAPT2+(CCD)δMP2 for all compounds. A-F model derived in this work based on training the split-charge equilibration method[S16,S17] on dimer energies (see Table 2).

References

(S1) National Institute of Standards and Technology, Diatomic Spectral Database Holdings. http://physics.nist.gov/cgi-bin/MolSpec/diperiodic.pl, 2018.

(S2) Sherrill, C. D. Energy Component Analysis of π Interactions. *Accounts Chem. Res.* **2013**, *46*, 1020–1028.

(S3) Heyrovska, R. Dependence of ion–water distances on covalent radii, ionic radii in water and distances of oxygen and hydrogen of water from ion/water boundaries. *Chem. Phys. Lett.* **2006**, *429*, 600–605.

(S4) Temelso, B.; Archer, K. A.; Shields, G. C. Benchmark structures and binding energies of small water clusters with anharmonicity corrections. *J. Phys. Chem. A* **2011**, *115*, 12034–12046.

(S5) Horn, H. W.; Swope, W. C.; Pitera, J. W.; Madura, J. D.; Dick, T. J.; Hura, G. L.; Head-Gordon, T. Development of an improved four-site water model for biomolecular simulations: TIP4P-Ew. *J. Chem. Phys.* **2004**, *120*, 9665–9678.

(S6) Verstraelen, T.; Vandenbrande, S.; Heidar-Zadeh, F.; Vanduyfhuys, L.; Van Speybroeck, V.; Waroquier, M.; Ayers, P. W. Minimal Basis Iterative Stockholder: Atoms in Molecules for Force-Field Development. *J. Chem. Theory Comput.* **2016**, *12*, 3894–3912.

(S7) Lamoureux, G.; Harder, E.; Vorobyov, I. V.; Roux, B.; MacKerell, Jr., A. D. A polarizable model of water for molecular dynamics simulations of biomolecules. *Chem. Phys. Lett.* **2006**, *418*, 245–249.

(S8) Yu, H.; Whitfield, T. W.; Harder, E.; Lamoureux, G.; Vorobyov, I.; Anisimov, V. M.; MacKerell, Jr., A. D.; Roux, B. Simulating Monovalent and Divalent Ions in Aqueous

Solution Using a Drude Polarizable Force Field. *J. Chem. Theory Comput.* **2010**, *6*, 774–786.

(S9) Walz, M. M.; Ghahremanpour, M. M.; van Maaren, P. J.; van der Spoel, D. Phase-transferable force field for alkali halides. *J. Chem. Theory Comput.* **2018**, *14*, 5933–5948.

(S10) Bayly, C. I.; Cieplak, P.; Cornell, W. D.; Kollman, P. A. A Well-Behaved Electrostatic Potential Based Method Using Charge Restraints for Deriving Atomic Charges - the RESP Model. *J. Phys. Chem.* **1993**, *97*, 10269–10280.

(S11) Jakalian, A.; Bush, B. L.; Jack, D. B.; Bayly, C. I. Fast, efficient generation of high-quality atomic charges. AM1-BCC model: I. Method. *J. Comput. Chem.* **2000**, *21*, 132–146.

(S12) Itskowitz, P.; Berkowitz, M. L. Chemical potential equalization principle: direct approach from density functional theory. *J. Phys. Chem. A* **1997**, *101*, 5687–5691.

(S13) Mortier, W. J.; Ghosh, S. K.; Shankar, S. Electronegativity-equalization method for the calculation of atomic charges in molecules. *J. Amer. Chem. Soc.* **1986**, *108*, 4315–4320.

(S14) Rappé, A. K.; Goddard III, W. A. Charge Equillibration for Molecular Dynamics Simulations. *J. Phys. Chem.* **1991**, *95*, 3358–3363.

(S15) Chelli, R.; Procacci, P.; Righini, R.; Califano, S. Electrical response in chemical potential equalization schemes. *J. Chem. Phys.* **1999**, *111*, 8569–8575.

(S16) Nistor, R. A.; Polihronov, J. G.; Müser, M. H.; Mosey, N. J. A generalization of the charge equilibration method for nonmetallic materials. *J. Chem. Phys.* **2006**, *125*, 094108.

(S17) Verstraelen, T.; Van Speybroeck, V.; Waroquier, M. The electronegativity equalization method and the split charge equilibration applied to organic systems: Parametrization, validation, and comparison. *J. Chem. Phys.* **2009**, *131*, 044127.

(S18) Jensen, F. Unifying Charge-Flow Polarization Models. *J. Chem. Theory Comput.* **2023**, *19*, 4047–4073.

(S19) Chen, J.; Hundertmark, D.; Martínez, T. J. A unified theoretical framework for fluctuating-charge models in atom-space and in bond-space. *J. Chem. Phys.* **2008**, *129*, 214113.

(S20) van der Spoel, D.; Marrades, J.; Kříž, K.; Hosseini, A. N.; Nordman, A. T.; Martins, J. P. A.; Walz, M.-M.; van Maaren, P. J.; Ghahremanpour, M. M. Evolutionary Machine Learning of Physics-Based Force Fields in High-Dimensional Parameter-Space. *Digit. Discovery* **2025**, *4*, 1925–1935.

(S21) Verstraelen, T.; Ayers, P. W.; Van Speybroeck, V.; Waroquier, M. ACKS2: Atom-condensed Kohn-Sham DFT approximated to second order. *J. Chem. Phys.* **2013**, *138*, 074108.

(S22) Chałasiński, G.; Szcześniak, M. M. State of the art and challenges of the ab initio theory of intermolecular interactions. *Chem. Rev.* **2000**, *100*, 4227–4252.

(S23) Jeziorski, B.; Moszynski, R.; Szalewicz, K. Perturbation Theory Approach to Intermolecular Potential Energy Surfaces of van der Waals Complexes. *Chem. Rev.* **1994**, *94*, 1887–1930.

(S24) McDaniel, J. G.; Schmidt, J. Physically-Motivated Force Fields from Symmetry-Adapted Perturbation Theory. *J. Phys. Chem. A.* **2013**, *117*, 2053–2066.

(S25) Van Vleet, M. J.; Misquitta, A. J.; Stone, A. J.; Schmidt, J. R. Beyond Born-Mayer:

Improved Models for Short-Range Repulsion in ab Initio Force Fields. *J. Chem. Theory Comput.* **2016**, *12*, 3851–3870.

(S26) Van Vleet, M. J.; Misquitta, A. J.; Schmidt, J. R. New Angles on Standard Force Fields: Toward a General Approach for Treating Atomic-Level Anisotropy. *J. Chem. Theory Comput.* **2018**, *14*, 739–758, PMID: 29266931.

(S27) Mulliken, R. S. Electronic population analysis on LCAO-MO molecular wave functions. I. *J. Chem. Phys.* **1955**, *23*, 1833–1840.

(S28) Hirshfeld, F. L. Bonded-atom fragments for describing molecular charge densities. *Theor. Chem. Acc.* **1977**, *44*, 129–138.

(S29) Marenich, A. V.; Jerome, S. V.; Cramer, C. J.; Truhlar, D. G. Charge Model 5: An Extension of Hirshfeld Population Analysis for the Accurate Description of Molecular Interactions in Gaseous and Condensed Phases. *J. Chem. Theory Comput.* **2012**, *8*, 527–541.

(S30) Besler, B. H.; Merz Jr., K. M.; Kollman, P. A. Atomic Charges Derived from Semiempirical Methods. *J. Comput. Chem.* **1990**, *11*, 431–439.
